# Spaceborne Cloud and Precipitation Radars: Status, Challenges, and Ways Forward

**DOI:** 10.1029/2019RG000686

**Published:** 2020-07-13

**Authors:** Alessandro Battaglia, Pavlos Kollias, Ranvir Dhillon, Richard Roy, Simone Tanelli, Katia Lamer, Mircea Grecu, Matthew Lebsock, Daniel Watters, Kamil Mroz, Gerald Heymsfield, Lihua Li, Kinji Furukawa

**Affiliations:** ^1^ National Centre for Earth Observation University of Leicester Leicester UK; ^2^ Earth Observation Science, Department of Physics and Astronomy University of Leicester Leicester UK; ^3^ DIATI Politecnico di Torino Turin Italy; ^4^ School of Marine and Atmospheric Sciences State University of New York at Stony Brook New York NY USA; ^5^ Institute for Geophysics and Meteorology University of Cologne Cologne Germany; ^6^ National Observatory of Athens Athens Greece; ^7^ Jet Propulsion Laboratory California Institute of Technology Pasadena CA USA; ^8^ Environmental and Climate Sciences Department Brookhaven National Laboratory Upton NY USA; ^9^ GESTAR, Morgan State University Laboratory for Atmospheres NASA Goddard Space Flight Center Greenbelt MD USA; ^10^ NASA Goddard Space Flight Center Greenbelt MD USA; ^11^ Space Technology Directorate I Japan Aerospace Exploration Agency Tokyo Japan

**Keywords:** radar, precipitation, convection, Doppler, cloud microphysics

## Abstract

Spaceborne radars offer a unique three‐dimensional view of the atmospheric components of the Earth's hydrological cycle. Existing and planned spaceborne radar missions provide cloud and precipitation information over the oceans and land difficult to access in remote areas. A careful look into their measurement capabilities indicates considerable gaps that hinder our ability to detect and probe key cloud and precipitation processes. The international community is currently debating how the next generation of spaceborne radars shall enhance current capabilities and address remaining gaps. Part of the discussion is focused on how to best take advantage of recent advancements in radar and space platform technologies while addressing outstanding limitations. First, the observing capabilities and measurement highlights of existing and planned spaceborne radar missions including TRMM, CloudSat, GPM, RainCube, and EarthCARE are reviewed. Then, the limitations of current spaceborne observing systems, with respect to observations of low‐level clouds, midlatitude and high‐latitude precipitation, and convective motions, are thoroughly analyzed. Finally, the review proposes potential solutions and future research avenues to be explored. Promising paths forward include collecting observations across a gamut of frequency bands tailored to specific scientific objectives, collecting observations using mixtures of pulse lengths to overcome trade‐offs in sensitivity and resolution, and flying constellations of miniaturized radars to capture rapidly evolving weather phenomena. This work aims to increase the awareness about existing limitations and gaps in spaceborne radar measurements and to increase the level of engagement of the international community in the discussions for the next generation of spaceborne radar systems.

## Key Points

- Current spaceborne radars offer a unique three‐dimensional view of the atmospheric component of the Earth's hydrological cycle
- Current spaceborne radar have limitations with respect to observations of low‐level clouds, midlatitude/high‐latitude precipitation, and convection
- Recent advances in radar technology will enable filling current science gaps

## 1 Introduction

The *holistic understanding of the Earth's water and energy cycles* remains one of the grand challenges that the international scientific community needs to address in the next decade. Three (out of seven) of the grand challenges posed by the World Climate Research Program (http://wcrp-climate.org/grand-challenges) are in fact centered around this theme: (1) Clouds, Circulation and Climate Sensitivity, (2) Understanding and Predicting Weather and Climate Extremes, and (3) Water for the Food Baskets of the World. These challenges require improving our skill in observing and ultimately predicting when, where, and why clouds form, whether they precipitate or not, and, if they do, how much precipitation they generate in the current climate and how this might evolve in a warming climate; this is not an easy task as specific hurdles exist in all these areas.

**Figure 1:**
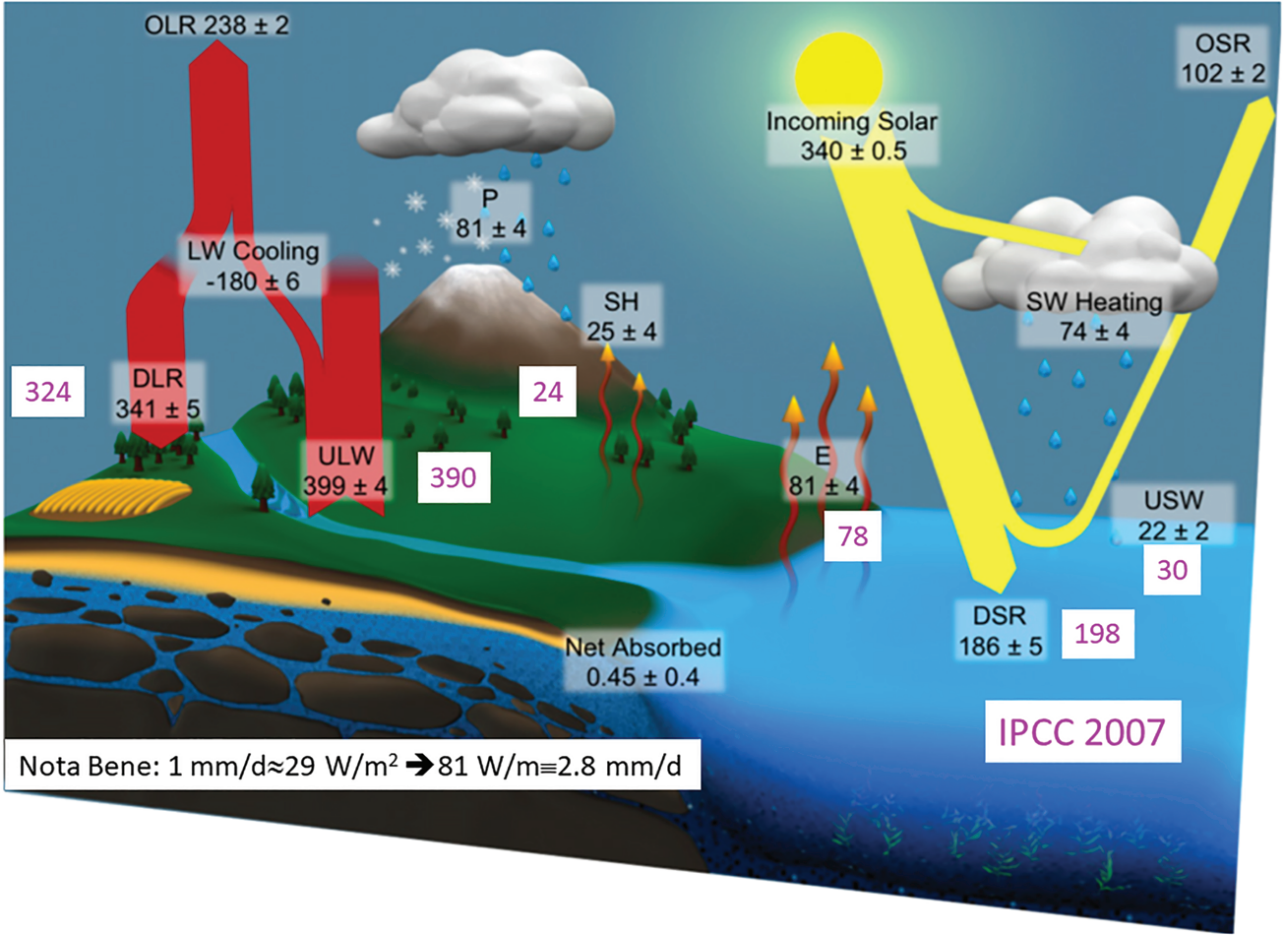
Global mean energy budget for the first decade of the 21st century based on independent flux measurements. The numbers state magnitudes of the individual energy fluxes in W/m^2^, adjusted within their uncertainty ranges to close the energy and water budgets. The surface fluxes from IPCC 2007 are also included in pink for reference. Adapted from (L'Ecuyer et al., 2015). ©American Meteorological Society. Used with permission.

Clouds play a critical role in the Earth's radiative budget through the reflection, absorption and emission of radiation, and through the vertical release of latent heat (Figure 1). Clouds are equally fundamental in the hydrological cycle by redistributing moisture and generating precipitation. Because of their central role in the energy and water cycle, uncertainties related to the representation of cloud microphysical properties and cloud feedbacks contribute the largest source of uncertainty in climate sensitivity estimates (Stephens, 2005; International Panel for Climate Change, 2013). In order to reduce uncertainty, microphysical variables such as the amount of condensed water, particle shape/size and dispersion of the particle size distribution (PSD) need to be known with reasonable accuracy at the cloud‐scale.

Precipitation, which encompasses both rain and snow, is also a central element of both the global water and energy budgets with a global annual mean equivalence between precipitation and evaporation which leads to a latent heat release in the atmosphere of 29 W/m^2^ for each 1 mm/day of liquid precipitation (Figure 1). On a regional scale, precipitation also affects ecological systems and freshwater resources and impacts urban and coastal areas especially during high‐impact weather events including hurricanes, floods, droughts and landslides. Observations have revealed that, while some climate models may be able to represent global land precipitation accumulation, they do so through an incorrect combination of frequency (too many) and intensity (not intense enough), thus generating regional biases (Dai, 2006; Stephens et al., 2010). Dai and Trenberth (2004) attributed their challenges in representing the diurnal cycle of surface precipitation both over land and over ocean to the production of cloud types that differ from those observed. It is particularly difficult to accurately simulate light and shallow precipitation processes that are increasingly important toward the poles. Improvements in precipitation predictability are likely to require adjustments to both cloud dynamics and microphysical processes parameterizations since those are responsible for hydrometeor growth and decay. In this regards, additional observations are needed to improve our understanding of clouds and precipitation and their formation and evolution in relation to the environment conditions and to constrain and improve numerical simulations.

Since the early days of outer space deployments, satellites have been the foremost tools for accurately estimating the global distribution of clouds and precipitation. This is especially true over the oceans and in land remote areas where it is difficult to deploy ground‐based sensors. From the beginning, the lion's share of satellite‐based cloud and precipitation measurements have been focused on the top of the atmosphere by conducting passive measurements of the solar radiation reflected by the Earth's atmosphere (also known as short‐wave radiation) as well as the thermal radiation emitted by the Earth's atmosphere (also known as longwave radiation). However, such measurements provide limited information about the vertical distribution of hydrometeors in the atmospheric column.

Active sensors in the microwave spectrum were deployed to meet this need since, contrary to infrared and visible, microwave radiation can penetrate clouds and precipitation and detail their vertical structure. The development of microwave radars for the study of clouds and precipitation started almost 70 years ago. Early efforts focused on the development of centimeter‐wavelength radars (3–10 cm) capable of probing large areas with precipitation while experiencing limited attenuation. Since then, a large body of theoretical and experimental work has been accomplished to the point that most of the measurable properties of radar signals—amplitude, phase, polarization, and frequency—can be interpreted in terms of the sizes, shapes, motions, or thermodynamic phase of the precipitation particles (Atlas, 1990). Furthermore, significant advancements in radar technology and digital signal processing have led to the development of sophisticated weather radar systems that provide high quality radar observables for operational and research weather and mesoscale meteorology application (Wakimoto & Srivastava, 2003). Recent developments, with emphasis on Doppler and polarimetric techniques and applications can be found in Doviak and Zrnić (1993), Bringi and Chandrasekar (2001), Lhermitte (2002), and Fabry (2015).

A bit later (1970s and 1980s), a smaller but energetic research community focused in the development of radar operating at millimeter wavelengths (8.6 and 3.2 mm) often called “cloud radars.” Their sensitivity to cloud droplets and small ice crystals arises from their short wavelength and the fact that in the Rayleigh scattering regime the hydrometeor cross section depends on the 1/*λ* ^4^. Today, millimeter‐wavelength radars along with lidar systems are the principal tools for the study of clouds (Clothiaux et al., 1995; Krofli & Kelly, 1996; Lhermitte, 1990). In the mid‐1990s the U.S. Department of Energy (DOE) Atmospheric Radiation Measurement (ARM) program deployed vertically pointing 35 GHz Doppler radars as the centerpiece of their observing instruments (Kollias et al., 2005; Kollias et al., 2007). The early version of the millimeter‐wavelength cloud radars (MMCRs) were designed, developed, and placed at the ARM sites by the National Oceanic Atmospheric Administration Environmental Technology Laboratory (Moran et al., 1998). Kollias et al. (2016) provides a detail historic progression of the DOE ARM MMCR program.

One pivotal moment on the use of centimeter‐ and millimeter‐wavelength radars for cloud and precipitation studies was the launch of the CloudSat Cloud Profiling Radar (CPR) mission in 2006 which featured the first 94 GHz radar in space. From the first moment the CPR transmitted in space (see Figure 1 Tanelli et al., 2008) it was clear that millimeter‐wavelength radars bridge an observational gap in Earth's hydrological cycle by adequately detecting clouds and precipitation, thus offering a unique and more holistic view of the water cycle in action (Kollias et al., 2007). From space, the millimeter‐wavelength signal needs to propagate only 12–20 km into the troposphere to detect the vertical structure of clouds and precipitation. It was the first time that a spaceborne radar system was able to capture both clouds and associated precipitation, thus breaking the dichotomy between “precipitation” and “cloud” radars. Soon after, ground‐based facilities were equipped with multiwavelength capabilities (centimeter‐ and millimeter‐wavelength radars; see Table 1 for band nomenclature) to capture both clouds and precipitation. Examples of such facilities include those operated by the DOE ARM program, the JOYCE facility, the Chilbolton Observatory and the Barbados Cloud Observatory (Brown & Lewis, 2005; Kollias et al., 2016, 2020; Löhnert et al., 2015; Stevens et al., 2016).

**Table 1:** Radar Frequency Bands Relevant for This Review

Frequency band nomenclature	Frequency range (GHz)	Wavelength range (mm)
X	8 – 12	25.0 – 37.5
Ku	12 – 18	16.7 – 25.0
K	18 – 27	11.1 – 16.7
Ka	27 – 40	7.5 – 11.1
W	75 – 110	2.7 – 4.0
G	110 – 300	1.0 – 2.7

Short wavelengths also enable narrow beamwidths with small antennas: The portability and compact size of centimeter‐ and millimeter‐wavelength radars make them a powerful research tool that can be deployed on various platforms, including ships, aircraft, and satellites. However, the deployment of radars in space was preceded by the development of extensive airborne cloud and precipitation radar platforms that collected critical data in support to mission concepts such as TRMM, CloudSat and then GPM. The first ground‐breaking airborne dual‐frequency rain radar‐radiometer system, developed in the 1980s by the Communications Research Laboratory (CRL) of Japan, paved the way toward the design of the first spaceborne rain radar (Okamoto et al., 1982). Afterward several data sets were acquired by single‐band or dual‐band radar systems such as ARMAR, ELDORA/ASTRAIA, EDOP, CRS, ACR, and others (e.g., Durden et al., 1994; Hildebrand et al., 1993; Heymsfield et al., 1996). In the Wakasa Bay experiment the APR‐2 (Ku and Ka band Sadowy et al., 2003) and the ACR (W band;  Sadowy et al., 1997) were installed in the forward and aft sections of the NASA P‐3, thus providing the first triple‐frequency airborne observations (Kulie et al., 2014). While small airborne data sets of three or even four radar frequencies were acquired during other field experiments (e.g., TC4, Costa Rica 2007; see  Jensen et al., 2009; Toon et al., 2010) by flying in formation two aircrafts carrying radars operating at different bands, the community had to wait until IPHEX/RADEX'14 (https://pmm.nasa.gov/iphex) for the first ever four‐frequency airborne data set of multifrequency radar data with frequencies ranging from X to W band on board the NASA ER‐2 (e.g.,  Battaglia et al., 2016). Later, the OLYMPEx/RADEx campaign in 2015 (Houze et al., 2017) with the NASA DC‐8 carrying the APR‐3 triple‐frequency scanning radar (Ku, Ka, and W band), the NASA ER‐2 carrying the EXRAD, HIWRAP and CRS, and the University of North Dakota Citation flying under them with cloud probes has been recognized by the scientific community as an unparalleled trove of information on cold‐season and orographic precipitating events (Chase et al., 2018a; Heymsfield et al., 2018; Tridon et al., 2019). Multifrequency radars have been irreplaceable assets in all NASA suborbital field campaigns ever since.

The deployment of such system in space to cover global scale did not start until the late 1990s. Space operating requirements and the cost associated with developing space‐qualified components with high technology readiness level (TRL) explain some of the latency in developing the first spaceborne missions and the slow rate at which subsequent missions have been being launched. The launches and the extended missions of the first two radars, the Tropical Rainfall Measuring Mission (TRMM) precipitation radar and the CloudSat cloud profiling radar (see sections 2.1–2.3), have been paramount for two aspects. First, they have demonstrated the reliability and longevity of active spaceborne systems in space that were previously deemed to have short lifetimes. Second, they have been instrumental in refining our understanding of the energy and water budget (International Panel for Climate Change, 2013; L'Ecuyer et al., 2015; Stephens et al., 2012), for example, as highlighted by the substantial change in the past decade of the surface energy fluxes (compare pink and black values in Figure 1). The first notable modification, confirmed by an improved knowledge of cloud base heights from CloudSat observations, relates to the estimated down‐welling longwave flux which falls in the range 336–346 W/m^2^, 17 W/m^2^ larger than previous estimates mostly based on model outputs that have a known negative bias in low cloud coverage. The increased downward surface longwave flux is accompanied by a small decrease of the shortwave absorbed flux (164 down from 168 W/m^2^) and by an increase of 9 W/m^2^ in the up‐welling longwave flux. The surplus of 4 W/m^2^ of radiative flux into the surface of the new budget is mostly compensated by an increase of the latent heat flux. Since the annual global mean evaporation is balanced by the annual global precipitation amount, the increase in the latent heat flux of 4 W/m^2^ corresponds to an increase of precipitation of 0.14 mm/day (i.e., up 4%). Again, CloudSat measurements hinted in this direction by pinpointing at underestimation of light precipitation in the midlatitudes and a significative contribution (up to 4 W/m^2^) from snowfall at high latitudes. An overall increase in global mean precipitation by 5–8% have been now reported but not yet published by GEWEX.

Today, the need for complimentary, synergistic multiwavelength spaceborne radar observations to monitor Earths hydrological cycle is well understood and highlighted in this review article (see later Figures 11 and 24 and related discussion). Thus, international partnerships between the space agencies are as critical as ever if we aim to develop a concept/mission with sufficient measurement capabilities to satisfy the diverse requirements of the scientific community. Despite several advantages, spaceborne radars have limitations. One of the them is the detection of low‐level clouds (marine boundary layer clouds and high‐latitudes mixed‐phase clouds;  Lamer et al., 2020). To obtain the full structure of such clouds is a daunting challenge for all remote sensing approaches based on a single measurement principle (be it lidar, radiometer, visible imager, etc.). For radar observations the challenge is primarily related to discriminating two closely spaced targets in range of which one is very reflective (the surface) and one is very weak (the cloud). In addition, current precipitation estimates (especially snowfall) from spaceborne radars are poorly constrained. The Group on Earth Observations Global Earth Observation System of Systems stated that of 146 essential climate variables, precipitation has the highest priority (Shiermeier, 2010).

In section 2 the existing and planned spaceborne radar systems, their capabilities, products and highlights of their contributions are discussed. Section 3 focuses on identifying gaps in cloud and precipitation measurements from spaceborne radars. Finally, section 4 discusses future pathways based on technological advancements and new sampling strategies for addressing these gaps. Appendix A1 provides a primer in multiwavelength Doppler radar remote sensing where the main radar quantities are introduced and the physical principles underpinning multiwavelength Doppler cloud and precipitation radars are discussed.

## 2 Current Spaceborne Radar Systems

**Figure 2:**
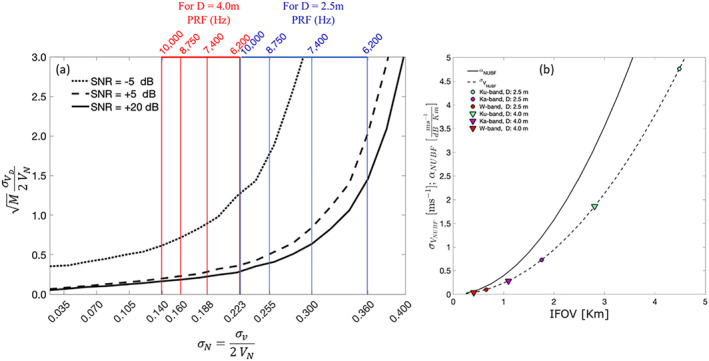
Mission timelines of TRMM, GPM, CloudSat, RainCube, and EarthCARE, together with the relevance of their radar operating bands to the detection of clouds and precipitation. ©Royal Meteorological Society and American Meteorological Society. Used with permission.

For more than two decades existing and planned spaceborne radar missions have provided and will continue to provide monitoring of the 3‐D structure of clouds and precipitation globally (Ackerman et al., 2018). A timeline of the launch and operational period of these missions is shown in Figure 2 and the technical specifications of the spaceborne radars are listed in Table 2. Exhaustive reviews of the missions can be found in the literature (Braun, 2011; Houze et al., 2015; Illingworth et al., 2015; Skofronick‐Jackson et al., 2018; Stephens et al., 2018a, and references therein); here we provide only few highlights to make the reader aware of the context of spaceborne system research. The expert reader can skip to section 3.

**Table 2:** Technical Specifications of Existing and Planned Spaceborne Radars

	Radar name					
	TRMM	GPM‐DPR		CloudSat	RainCube	EarthCARE
Specifications	PR	KuPR	KaPR	CPR	Radar	CPR
Mission duration	1997–2015	2014–		2006–	2018–2019	2022–
Frequency (GHz)	13.799 ± 0.003	13.60 ± 0.003	35.55 ± 0.003	94.05	35.75	94.05
Altitude (km)	350/403^a^	407	407	705	400	400
Orbit inclination (°)	35	65	65	98.2	51.6	97
Antenna (type)	Phased‐array	Phased‐array	Phased‐array	Reflector	Deployable	Reflector
Antenna (m)	2.0× 2.0	2.1 × 2.1	0.8 × 0.8	1.85	0.5	2.5
Transmitter type	Solid State	Solid State	Solid State	EIK	Solid State	EIK
Peak power (W)	500	446	344	1,500	N/A^b^	1,500
Frequency agility	Yes	Yes	Yes	No	No	No
Hor. res. at nadir (km)	5	5	5	1.2	8–10	500
Vert. resolution (m)	250	250	250/500^c^	500	250	500
Swath (km)	220	245	120/245^d^	N/A^e^	N/A	N/A
Polarization	Single	Single	Single	Single	Single	Single
Clutter height (m)	1,000	1,000	1,000	1,000	1,000	1,000
Doppler capability	N/A	N/A	N/A	N/A	No	Yes
Sensitivity (dBZ)	+17	+14	+13–18	−30	+11	−35

a The altitude of TRMM was increased from 350 to 403 km in 2001 in order to extend the lifetime of the mission.

b This radar employs only a 10 W peak power amplifier with 10% duty cycle to achieve an average power comparable with that of the Ka band channel of DPR.

c The KaPR operates in two modes: a high sensitivity HS‐mode (pulse length of 1,000 m) and a matched scan MS‐mode (pulse length of 500 m that matches the KuPR pulse).

d During the first phase of the GPM mission the KaPR has been scanning on a 120 km narrower swath. Since 21 May 2018 the KaPR scan pattern has been changed to match the KuPR swath, with the matched scan region centered on the ground track as before and the high sensitivity scans replaced to the outer swath region (i.e., no longer interlaced within the matched scan region).

e Not applicable.

### 2.1 TRMM

The Tropical Rainfall Measuring Mission (TRMM) satellite, launched in November 1997 was a joint mission by the National Aeronautics and Space Administration (NASA) and the Japanese Aerospace Exploration Agency (JAXA) successfully operated from 1997 to 2015, was the first weather radar in space (Okamoto, 2003). The TRMM Precipitation Radar (PR) was deployed on the satellite together with passive microwave, visible, infrared, and lightning sensors, for example (Kummerow et al., 1998; Simpson et al., 1996). Thanks to a phased‐array antenna, the PR was able to scan the atmosphere with a cross‐track field of view of 220 km, giving the first three‐dimensional measurements of weather systems in remote places. The TRMM PR operated at a single frequency (13.8 GHz) had no dual polarimetric or Doppler capabilities and was characterized by a sensitivity threshold of approximately 17 dBZ. TRMM, which terminated operations in 2015, has provided a legacy data set of 17 years of nearly uninterrupted measurements covering the tropics and subtropics. The satellite's primary goal was to provide the climate science community with comprehensive precipitation measurement in high spatial resolution over the entire ±35° low latitude belt where the majority of Earth's rainfall occurs. Measurements were actually available from slightly higher latitudes (±37°) because of the TRMM scan swath. Because most of the low latitudes are ocean, where surface precipitation gauges and ground‐based radars are absent, the tropical rain pattern was only approximately known prior to TRMM. TRMM sensor data have been combined with other satellite data to produce detailed rain maps and real‐time prediction products. TRMM data products, together with documentation, are available at https://pmm.nasa.gov/data-access/downloads/trmm.

#### 2.1.1 Highlights of TRMM Applications

**Figure 3:**
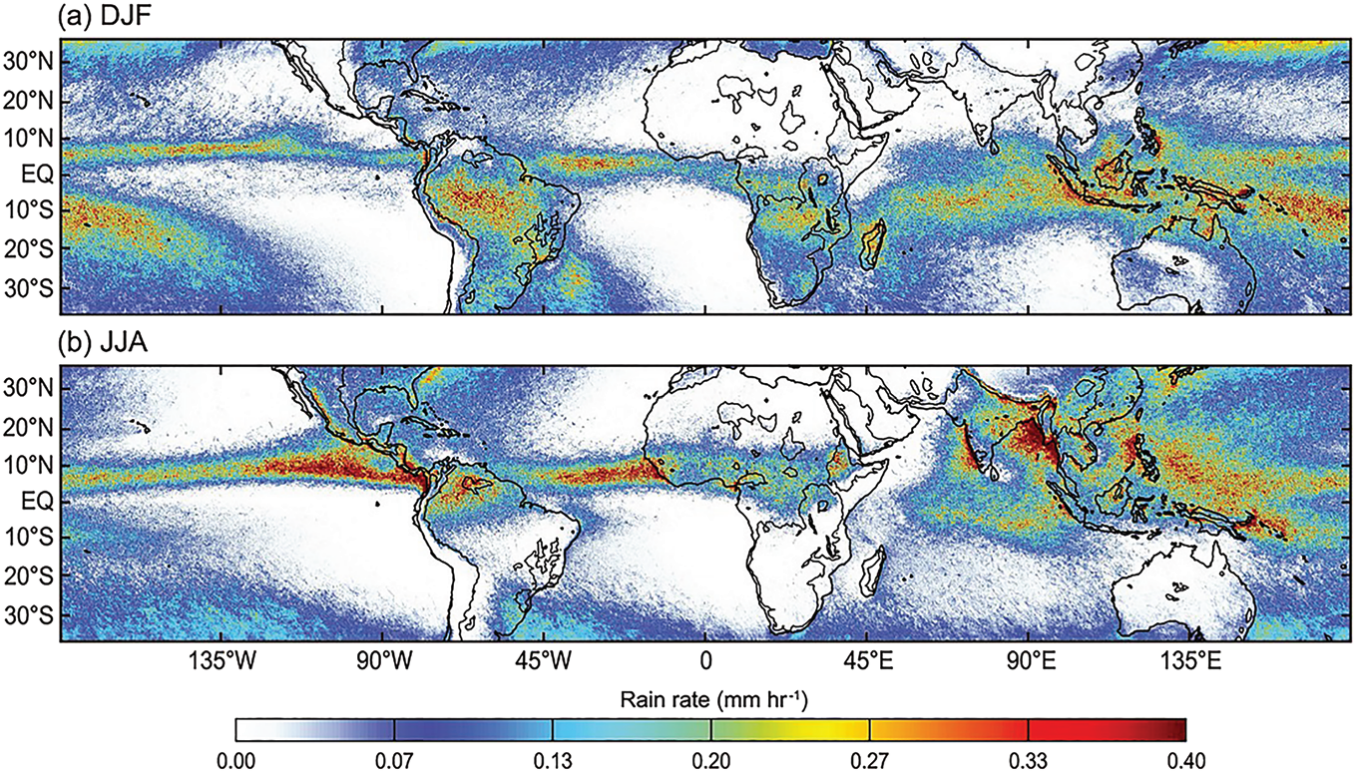
Precipitation climatology expressed in average rain rates (mm/hr) during the months of (a) December, January, and February (DJF) and (b) June, July, and August (JJA) from 1998 to 2013. The black contour inside the continental regions represents the 700 m elevation. Extracted from Houze et al. (2015) © American Geophysical Union. Used with permission.

*Maps of Precipitation in the Tropical Regions*. TRMM provided the first global characterization of tropical rainfall. The rain maps in Figure 3 (from Houze et al., 2015) show the average rain rate across the tropics and subtropics as seen by the PR for two seasons: boreal winter, December‐January‐February (DJF), and boreal summer, June‐July‐August (JJA), averaged over 16 years of the TRMM 2A25 near‐surface rain product (Iguchi et al., 2000). The rainfall is clearly most prevalent in the equatorial band, with a clear seasonal pattern associated with the movement of the intertropical convergence zone toward North during the boreal summer. The pattern associated to the South Pacific Convergence Zone extending from the maritime continent southeastward toward Polynesia is also quite prominent in the boreal winter. There are also notable enhancements in precipitation, particularly clear in JJA, near ocean/continent boundaries and associated with orography corresponding to the wettest places on Earth (e.g., the west coast of India, Guinea, Sierra Leone, Myanmar, Panama and Colombia).

**Figure 4:**
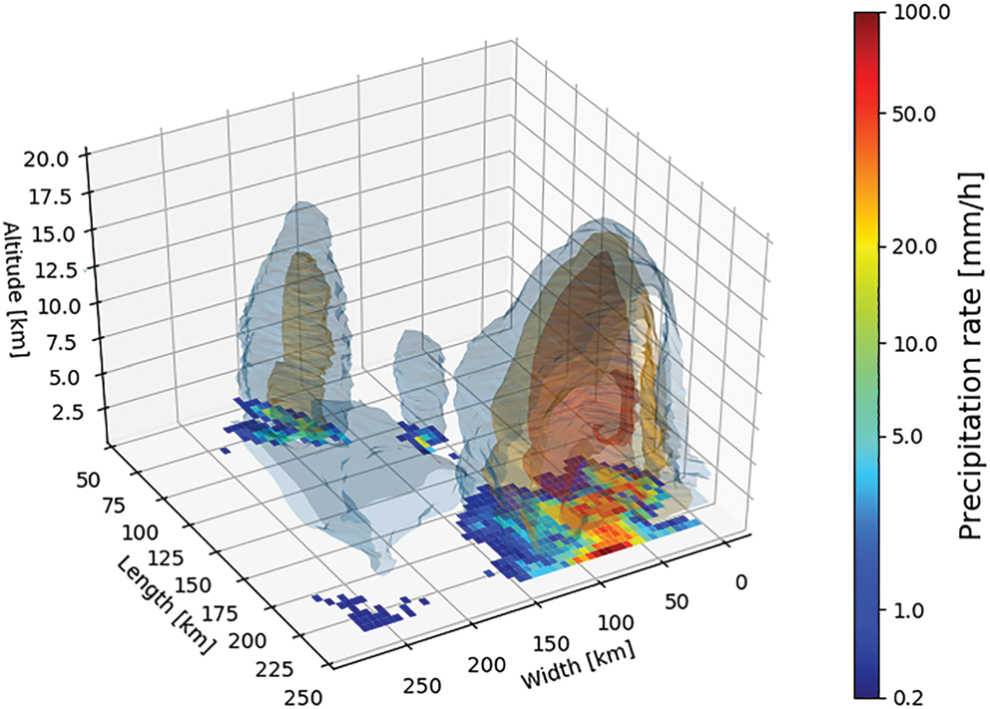
The “champion storm” studied by Zipser et al. (2006), which occurred in northern Argentina during December 1997. The detailed three‐dimensional structure of this storm is shown using the blue, orange, and red surfaces that represent 20, 30, and 40 dBZ radar reflectivity isosurfaces, respectively. The surface precipitation rate is also shown (color scale).

*Three‐Dimensional Views of Storm Systems*. The ranging capability of the PR combined with the cross‐track scanning strategy has enabled TRMM to resolve the three‐dimensional structure of storms at an unprecedented vertical resolution of 250 m. In pulsed radar systems the vertical resolution, Δ*r*, is related to the pulse length, *τ*_*p*_, via Δ*r* = *cτ*_*p*_/2. The received power is typically oversampled, for example, for GPM power samples are collected at 1.2 MHz corresponding to a 125 m sampling. Figure 4 depicts three‐dimensional structure of the “champion storm” described by Zipser et al. (2006), the most intense precipitation event in the TRMM PR record. This was a deep storm system that occurred on 30 Dec 1997 in northern Argentina. The radar cross section included a 40 dBZ echo reaching 19.5 km, with one location reaching 34 dBZ in the uppermost PR altitude level (20 km). These exceptional values suggest the presence of very large hail.

*Characterization of Storms*. This new capability led to the retrieval of the latent heat release in the atmosphere (see Figure 9 in  Tao et al., 2010 and  Tao et al., 2016), provided insight into structure of tropical cyclones (e.g., Hence & Houze, 2012; Tao & Jiang, 2013), quantified the occurrences of shallow convective systems (Houze et al., 2015) and intense storms (Zipser et al., 2006). Such systems occur almost exclusively over land mainly over the southeastern United States, Argentina (especially close to the Andes in summer), near‐equatorial Africa and America, and northwest India and Pakistan (close to the intersection of the Himalayas and mountains of Afghanistan). In contrast, shallow isolated cells occur mainly over the warm oceans and practically never over land. Furthermore, the deep, intense convection tends to occur in more arid regions, especially near, but not over, the high mountains of the Himalayas and Andes.

### 2.2 GPM

The joint NASA/JAXA Global Precipitation Measurement (GPM) mission aims at further understanding global precipitation and its spatio‐temporal variability as a core component of the Earth's weather, climate, and hydrological systems (Ackerman et al., 2018; Peters‐Lidard et al., 2018; Skofronick‐Jackson et al., 2018).

The GPM mission is the follow‐up mission to TRMM. The critical component of its observing system is the GPM Core Observatory (GPM‐CO) spacecraft (Hou et al., 2014), which mirrors the TRMM payload by including a radar and a radiometer on the same platform. However, both instruments are technologically ahead of their predecessors. The JAXA active sensor is upgraded to Dual‐frequency Precipitation Radar (DPR) that operates at Ku (13.6 GHz) and Ka (35.5 GHz) bands. The NASA contribution, that is, the GPM Microwave Imager (GMI), gained additional five high‐frequency bands ranging from 166 to 190 GHz and the channels of the GMI that duplicate those of the TMI have better spatial resolution. The performance of the GPM radars is slightly better than TRMM's with a minimum detectable signal of approximately 14 dBZ for both bands (see Table 2, Toyoshima et al. 2015). There are, however, three key novelties in the GPM mission compared to TRMM.

1. Thanks to its higher‐inclination orbit (65°), the GPM‐CO expands the TRMM measurements to higher latitudes and more overland regions. For the first time the 3‐D structure of precipitating systems, including moderate to intense snow events and extratropical cyclones, can be observed globally in the 35–65° latitudinal band.
2. The DPR is the first ever dual‐frequency atmospheric radar in space with increased potential compared to its TRMM‐PR predecessor in narrowing down uncertainties in rainfall estimates and in providing insight in rain and ice microphysics.
3. The GPM‐CO acts as the reference calibrator of a constellation of passive microwave radiometers (Berg et al., 2016). Such a constellation provides cross‐calibrated precipitation data with a much shorter revisit time than is possible with the GPM‐CO only. This cross‐calibrated precipitation data from microwave sources is a key input to NASA's IMERG product (Huffman et al., 2017), a gridded product at unprecedented temporal (30 min) and spatial (0.1° × 0.1°) resolutions that uses both GPM‐CO and partner satellite precipitation estimates along with geostationary infrared (IR) precipitation estimates to fill in gaps between the microwave satellite overpasses. The IMERG product is then adjusted by monthly gauge precipitation estimates from the Deutscher Wetterdienst Global Precipitation Climatology Centre (GPCC). JAXA produces an analogous product, the Global Satellite Mapping of Precipitation (GSMaP).

All GPM data are freely available at PPS (https://pmm.nasa.gov/data-access/downloads/gpm) while JAXA's GPM products can be obtained from https://gportal.jaxa.jp/gpr/ or http://sharaku.eorc.jaxa.jp/.

#### 2.2.1 Highlights of GPM Applications

**Figure 5:**
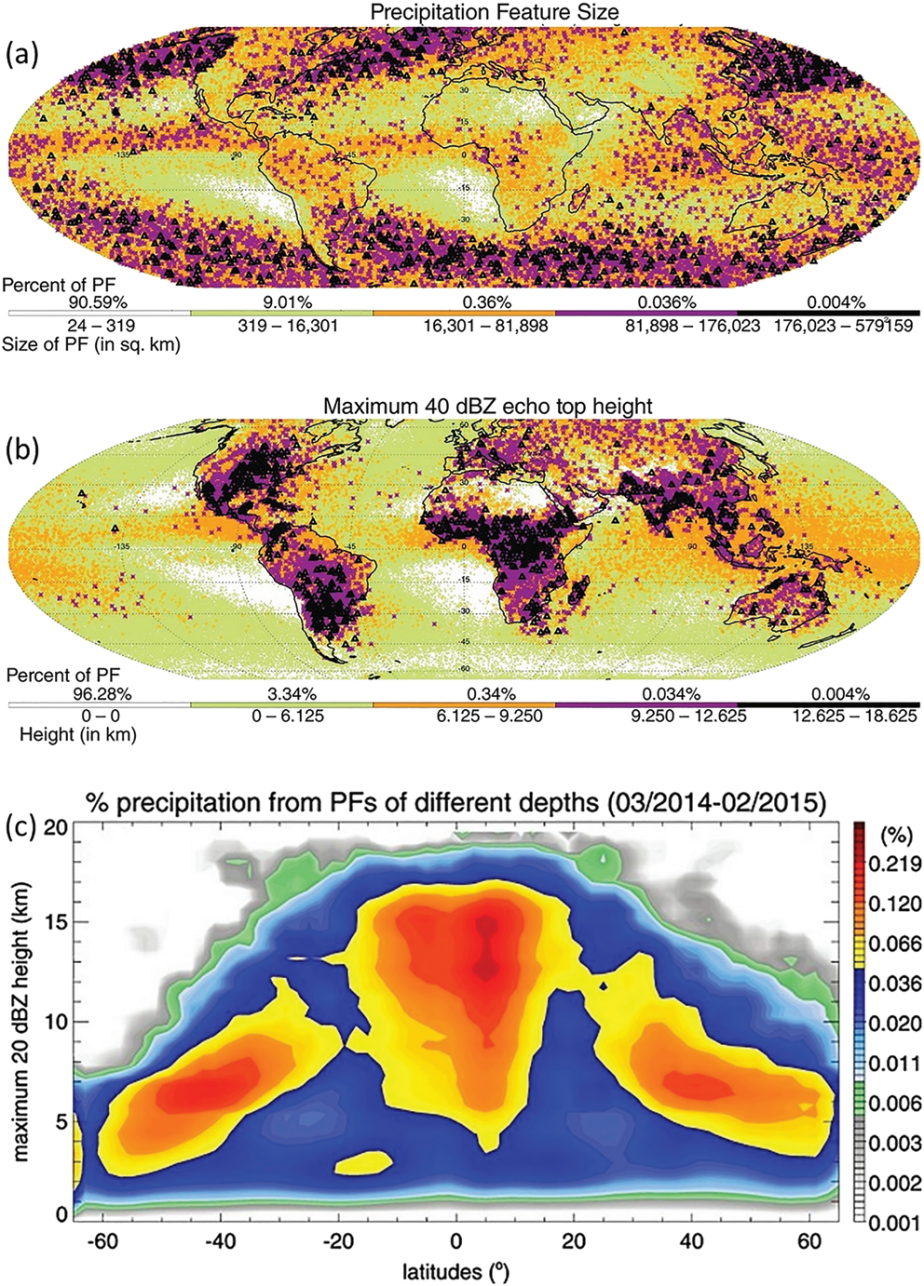
Locations of precipitation features (PFs) according to (a) their size, (b) 40 dBZ maximum echo top height as derived from the GPM‐CO Ku band data (adapted from Skofronick‐Jackson et al. (2018) ©Royal Meteorological Society. Used with permission). (c) Contributions to global precipitation from PFs of different maximum heights of the 20 dBZ echo (adapted from Liu and Zipser (2015) ©American Geophysical Union. Used with permission). The statistics are computed in 2° latitude bins, and total values add up to 100% per zonal bin. Note the logarithmic color scale.

*Extending TRMM's Legacy to Outside the Tropics*. The GPM‐DPR systems with its Ku component continues the record collected with the TRMM‐PR instrument and extends it to the extratropics. Therefore, scientific studies done in the tropics during the TRMM‐PR era can now be extended to the extratropics in the GPM‐DPR era. For example, DPR‐Ku data have been used to classify the largest, deepest and strongest precipitation systems on Earth (Liu & Zipser, 2015, see Figures 5a and 5b). The DPR confirms TRMM results, that precipitating storm systems in the Great Plains of the United States and the Pampas in Argentina are among the most intense on Earth (with similar findings reported in (Mroz et al., 2017) for hail‐bearing systems). Furthermore, GPM has quantified the contributions to precipitation at different latitudes from precipitation features (PFs) of different sizes and different heights (Liu & Zipser, 2015). The largest precipitation systems are found in the midlatitude extratropical storm tracks, highlighting the role of fronts in the organization of these systems.

Over the tropics deep PFs with 20 dBZ echo reaching above 12 km contribute a big portion of global precipitation, although they are relatively rare (Figure 5c). Despite the large number of occurrences of PFs with low echo tops, their precipitation contribution is not as significant. In the southern subtropics there is a high fraction of (weak and) shallow precipitation in regions with large‐scale descent (e.g., stratocumulus regions in the west coast of Chile and Africa). Shallow precipitation systems are also of significance over northern midlatitudes and high‐latitudes but with smaller precipitation contributions than their southern counterparts.

*Toward Microphysical Process Studies and Societal Applications* The GPM‐DPR provides novel measurements of the Ku‐Ka dual‐frequency ratio (DFR, for definition see section A0.2) that are enabling ice and rain microphysical process studies. By focusing on tropical cyclone precipitation over the western North Pacific, Huang and Chen (2019) analyzed the relationship between the concentrations of droplets in the sampling volume (associated with *N*_*w*_, the “generalized intercept parameter”) and the mean mass‐weighted raindrop size (*D*_*m*_) with respect to the rain type and precipitation efficiency index (i.e., the ratio of the near‐surface rain rate to the liquid water path). Compared with stratiform precipitation, convective systems tend to produce larger mean raindrop sizes (>2 mm) for precipitating clouds with the same precipitation efficiency index, and larger *N*_*w*_ for the same *D*_*m*_, indicating different rain processes below and/or different ice microphysics above the melting layer.

DFR has also potential to obtain microphysical properties associated with ice. Based on thresholds in Z and DFR, Iguchi et al. (2018) introduced a flag to identify profiles containing intense ice precipitation above the height of −10°C. The flag successfully detects intense ice precipitations not only in strong tropical convections but also in high‐latitude winter storms (see their Figure 4 for a climatology of the frequency of occurrences of such events). Ni et al. (2019) studied deep convective cores, defined as GPM precipitation features with maximum 20 dBZ echo‐top heights above 10 km. The DFR near the top of deep convective cores (e.g., 12 km) is positively related to the convective intensity and tends to be higher over subtropical land than over tropical land and over ocean. The highest values of DFR are found over the hot spots of the most intense convection (see Figure 5). Retrieved ice microphysical properties show a larger mean diameter, more ice water content, and lower ice particle number concentration over land than over ocean near the top of deep convective cores above 10 km. Note that caution must be used in microphysical studies, especially in convection where attenuation correction is still very uncertain and where second‐order effects (e.g., multiple scattering [MS], and nonuniform beam filling [NUBF]) are relevant (see section A0.1).

As well as providing valuable contributions to science, GPM near real time products, IMERG in primis, are now used to provide applications like hazard assessment for floods, landslides and droughts and to shed light upon societal issues like agricultural productivity, famine, and public health (Skofronick‐Jackson et al., 2018). Both microphysical process studies and societal applications are currently topics of active research.

### 2.3 CloudSat

On the spaceborne cloud observations front, NASA and the Canadian Space Agency (CSA) launched the first satellite‐borne (CloudSat) W band (94 GHz) cloud profiling radar (CPR; (Tanelli et al., 2008)) as part of NASA's A‐Train constellation (Stephens et al., 2008). CloudSat has been successfully collecting data since 2006. Though the primary objective of CloudSat‐CPR was to observe cloud geometric boundaries in order to improve estimates of radiative heating within the atmosphere and at the Earth's surface, the CPR also detects a wide range of precipitation, from the lightest drizzle and snow to moderate rainfall. These unique capabilities have shed new light onto several components of the Earth energy budget (Figure 1 and discussion therein) and have demonstrated the foundational role played by such missions for an holistic view of the water cycle. At the time of writing, CloudSat is still operational: The radar is still transmitting on its primary amplifier and is operated in the so‐called Daylight Only mode since 2011 due to the aged battery on the spacecraft; the spacecraft is maintaining the new C‐Train with Calipso since 2018.

The CloudSat data products, together with their descriptions and data product levels, are currently available from the CloudSat Data Processing Center (http://www.cloudsat.cira.colostate.edu/data-products).

#### 2.3.1 Highlights of CloudSat Applications

**Figure 6:**
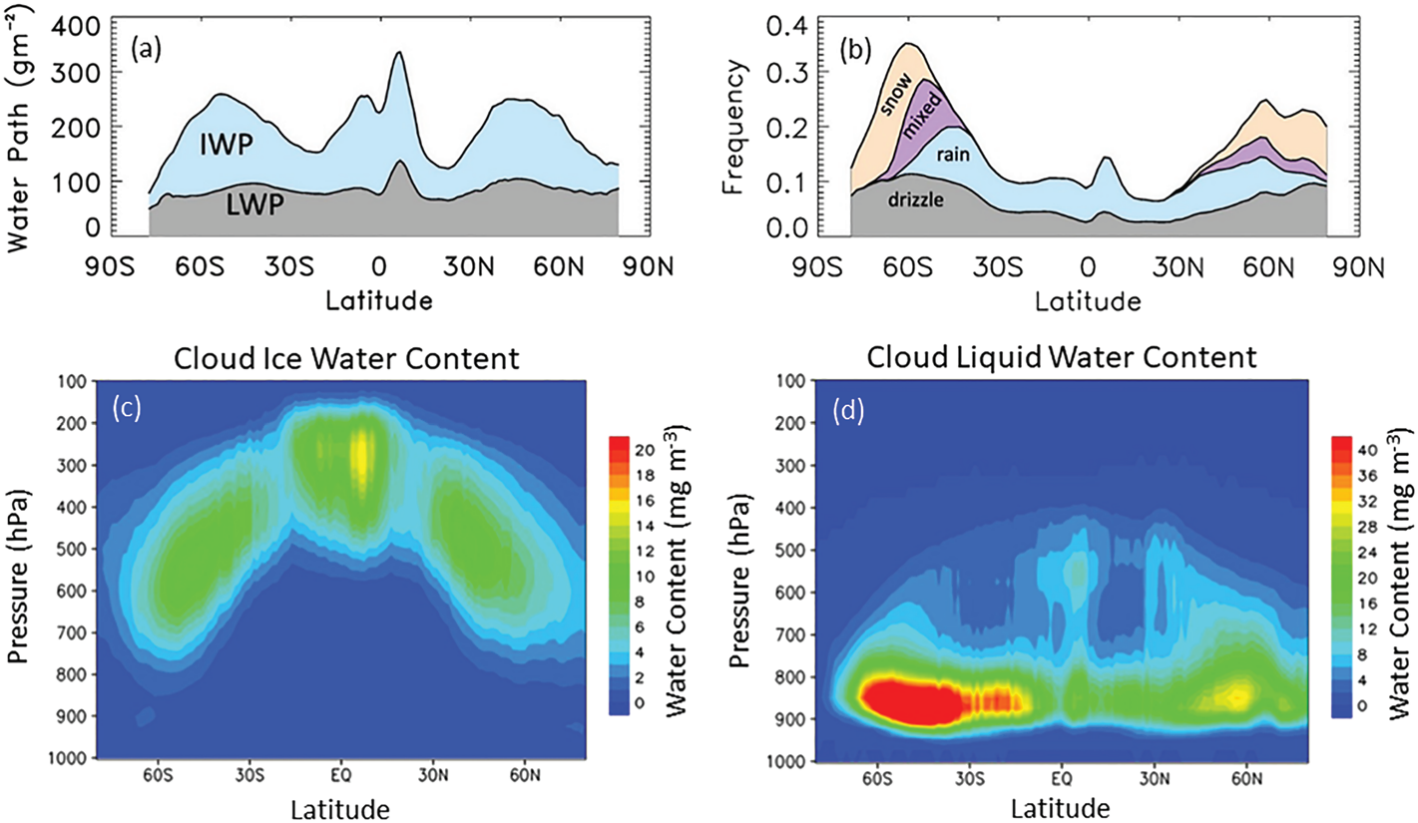
(a) A multiyear annual zonal‐mean liquid water path (gray shading, ocean only O'Dell et al., 2008) and ice water path (blue shading, from CloudSat 2C‐ICE data set for 2006–2010). (b) A multiyear annual‐mean precipitation fractional occurrence from CloudSat 2C‐COLUMNPRECIP. (c and d) Latitude‐pressure sections of annual mean cloud ice water and liquid water content (Lee et al., 2014). Adapted from Stephens et al. (2018b) and Lee et al. (2014) ©American Meteorological Society and Springer Nature. Used with permission.

*Global Distribution of Clouds and Precipitation*. Figure 6 shows cloud and precipitation zonal average profiles derived from combined CloudSat radar and CALIPSO lidar observations (Stephens et al., 2018b). Cloud liquid and ice water paths are presented in Panel a, the frequency of occurrence of precipitation by type in Panel b whereas vertical profiles of cloud ice and liquid are shown in Panels c‐d. Precipitation systems are much deeper on average in the tropics (Figure 6d), and the frequency of precipitation is strongly enhanced in middle and higher latitudes, with snowfall becoming the prevalent mode of precipitation poleward of about 60°N and 60°S (Figure 6b). These observations provides important constraints for evaluating global models (e.g., Chen et al., 2011).

**Figure 7:**
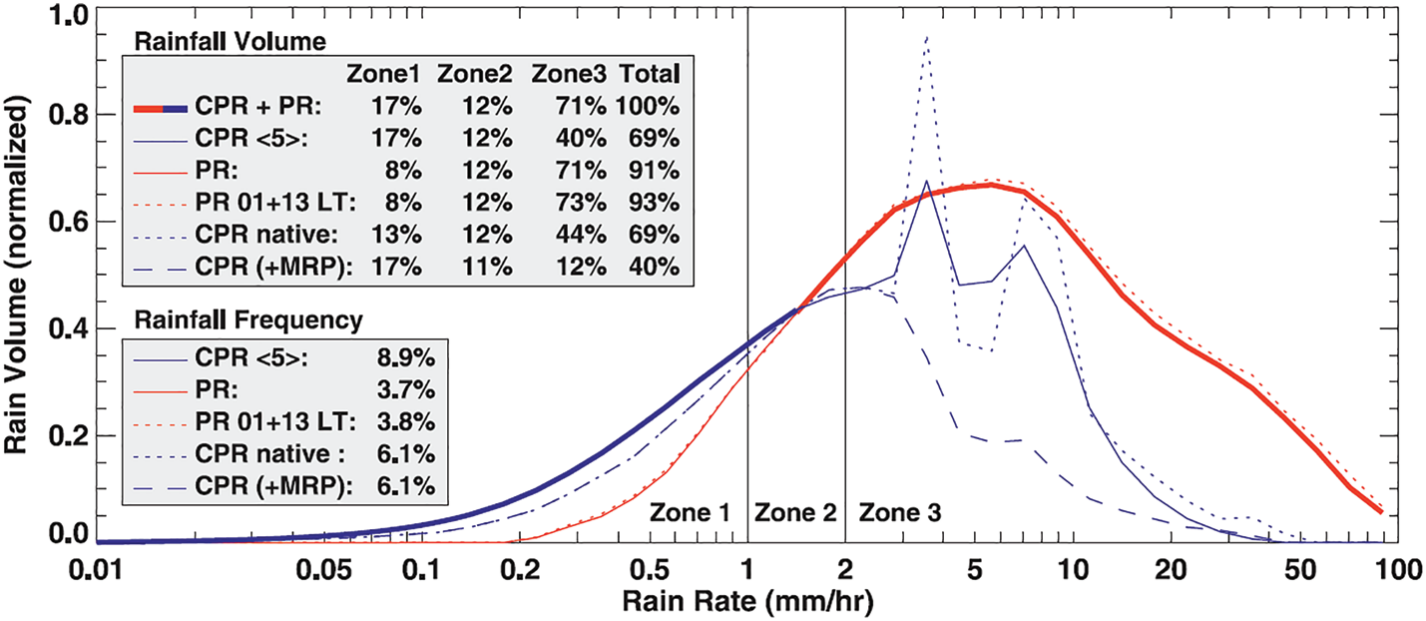
The distribution of the total rain volume from the CPR and PR as a function of rainfall intensity over tropical and subtropical oceans (35°S to 35°N) for the period from June 2006 through February 2009. Extracted from Berg et al. (2010) ©American Meteorological Society. Used with permission.

*First Statistics of Drizzle and Snow*. CloudSat has complemented the precipitation radars flown in TRMM and GPM by providing the first ever statistics of drizzle, light rain and snow (Stephens et al., 2018b). The 13.8 GHz TRMM Precipitation Radar (PR) and the 94 GHz CloudSat Cloud Profiling Radar (CPR) provide highly complementary information: The PR provides the best information on the total rain volume because of its ability to estimate the intensity of all but the lightest rain rates (the sensitivity of the PR is about 17 dBZ), while the CPR's higher sensitivity provides superior rainfall detection as well as estimates of drizzle and light rain (Berg et al., 2010, and Figure 7). Haynes et al. (2009) demonstrated that CPR data is able to deliver meaningful estimates of surface rain rate to about 3 mm/hr and perhaps up to about 5–8 mm/hr by including path integrated attenuation information. However, the CPR substantially underestimates rain from intense convective storms because of large attenuation and multiple scattering effects (Battaglia et al., 2007; Battaglia et al., 2008) while the PR misses very little of the total rain volume because of a lower relative contribution from light rain. Over the oceanic portions of the TRMM region between 35°S and 35°N, rainfall frequency from the CPR is around 9%, approximately 2.5 times that detected by the PR, and the CPR estimates indicate a contribution by light rain that is undetected by the PR of around 10% of the total (Berg et al., 2010). Later, Behrangi et al. (2012) combined CloudSat and TRMM PR observations to estimate that the Global Precipitation Climatology Project had underestimated oceanic rainfall by between 4 and 8%.

The CloudSat mission has also provided the first ever global climatology of snow (Hiley et al., 2011; Palerme et al., 2014). However, single‐frequency radar algorithms rely on empirical relations between the equivalent radar reflectivity factor *Z*_*e*_ and snowfall rate, S, which are in turn a function of particle fall velocity, particle habit and PSD and can lead to uncertainties in snowfall estimate over 100% (Hiley et al., 2011).

**Figure 8:**
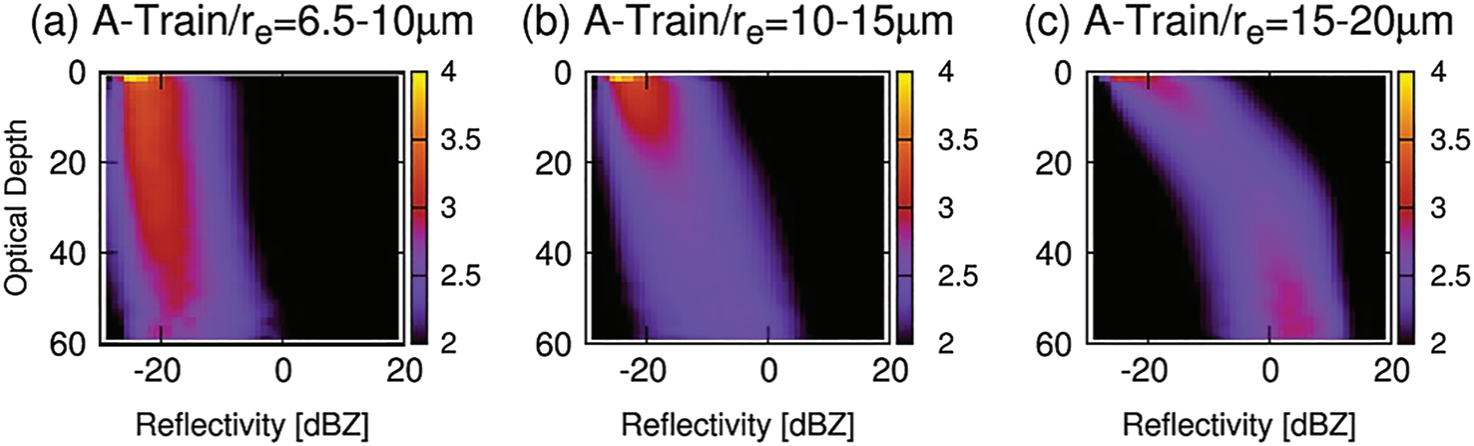
Vertical profiles of CloudSat radar reflectivity as a function of in‐cloud MODIS optical depth in the form of contoured frequency diagram classified according to cloudtop effective radius (*r*_*e*_) of (a) 6.5–10 (nonprecipitating clouds), (b) 10–15 (drizzling clouds), and (c) 15–20 μm (precipitating clouds). The probability distribution function of radar reflectivity normalized at each optical depth is shown using the color scale (%dBZ^−1^). Adapted from Suzuki et al. (2013) ©American Geophysical Union. Used with permission.

*Warm Rain Processes*. Cloudsat CPR in synergy with other A‐Train instruments has been instrumental in advancing our understanding of warm‐cloud physics (Stephens et al., 2019). The conversion rate of liquid water from cloud to rain is typically parameterized as a function of liquid water mixing ratio and number concentration, and its formulation involves a set of parameters determining how the conversion rate depends on the cloud properties. One of the most influential parameters contained in some parameterization schemes is the threshold particle radius above which the precipitation is assumed to form. This threshold radius serves as a switch triggering rain formation, thereby largely controlling the cloud water content after the precipitation occurs: A larger threshold radius inhibits rain formation and increases cloudiness. Vertical profiles of CloudSat radar reflectivity combined with MODIS cloud properties are presented in Figure 8 (Suzuki et al., 2013). The profiles of radar reflectivity are rescaled as a function of in‐cloud optical depth (ICOD). The probability density function of the radar reflectivity normalized at each ICOD bin is shown in the form of contoured frequency diagrams and classified according to different ranges of cloud‐top effective particle radius (*r*_*e*_), which is obtained from the MODIS level 2 MYD06 cloud product. The statistics derived in this manner are shown in Figures 8a–8c. The figure depicts the transition from nonprecipitating clouds (Figure 8a) through drizzling clouds (Figure 8b) to precipitating clouds (Figure 8c). The results show that the assumption of a threshold larger than 10 μm is most plausible in terms of reproducibility of microphysical transitions from nonprecipitating to precipitating clouds. Further studies extending this approach have shown that modern climate models have a common deficiency of initiating the rain formation process too quickly compared with observations (Suzuki et al., 2015). Similarly current climate models are unable to simultaneously simulate a realistic cloud‐rain transition and a physically plausible aerosol indirect effect, suggesting systematic biases exist in the representation of aerosol interactions with clouds in models (Jing et al., 2019).

### 2.4 RainCube

**Figure 9:**
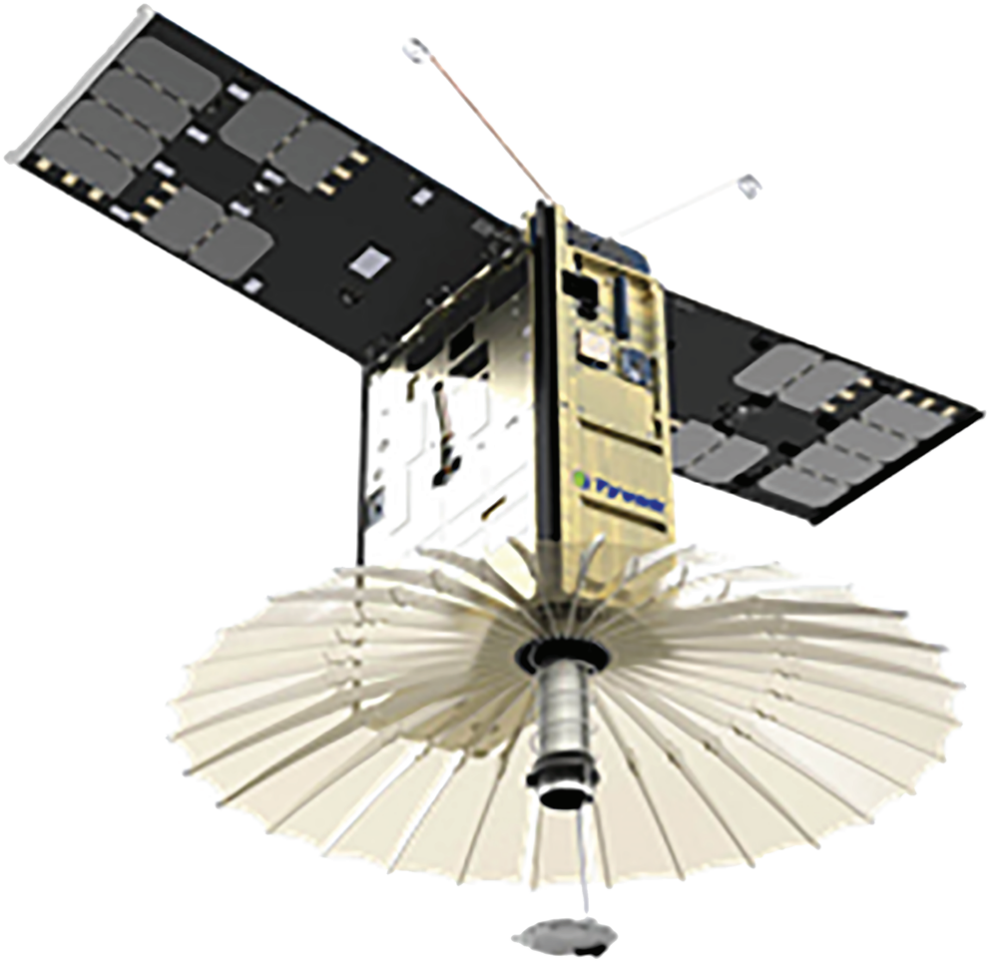
Image of RainCube in its fully deployed state.

When attempting to improve the temporal sampling frequency to time scales of minutes or tens of minutes, a single‐radar in LEO solution is insufficient. One possible solution that has been explored is that of deploying a radar in GEO. Such solution is ideal for systematic monitoring of low latitude regions of particular interest (e.g., the North Atlantic basin for Hurricane genesis and evolution), but it cannot be extended to truly global sampling and it does require significant resources in terms of size, mass and power because of the large distance from Earth. The alternative to deploying precipitation radars in GEO is to deploy several radars in LEO (as a convoy or constellation). This has not been realistically affordable for decades until the arrival of the smallsat and CubeSat platforms. At this point the challenge has moved to simultaneously miniaturizing, reducing costs and preserving fundamental performance requirements for this type of radar. Following these aims, a novel architecture compatible with the 6U class (or larger) has been developed at the NASA Jet Propulsion Laboratory (JPL). The Radar in a CubeSat (RainCube) architecture reduces the number of components, power consumption and mass by over one order of magnitude with respect to existing spaceborne radars. The RainCube mission (Peral et al., 2019; Figure 9 and Table 3) is a technology demonstration mission, launched on 21 May 2018, to enable Ka band (35.75 GHz) cloud and precipitation radar technologies on a low‐cost, quick‐turnaround platform. A 6U cubesat includes the radar electronics, the compact lightweight deployable 0.5 m antenna and the bus avionics systems, the radar itself occupies a volume of about 10 × 20 × 22 cm including the stowed antenna.

**Table 3:** RainCube High‐Level System Parameters

Requirement name	Requirement	Measured
Sensitivity at 400 km	20 dBZ	11.0 dBZ
Horizontal resolution at 400 km	10 km	7.9 km
Nadir data window	0–18 km	−3 to 20 km
Vertical resolution	250 m	250 m
Downlink data rate (in transit)	50 kbps	49.57 kbps
Payload power consumption	
(AntDeploy/STDBY/RXOnly/TXScience)	10/8/15/35 W	5/3/10/22 W
Mass	6 kg	5.5 kg
Range sidelobe suppression	> 60 dB at 5 km	> 65 dB at 1 km
Transmit power and transmit loss	10 W/1.1 dB	> 39 dBm
Antenna gain	42 dB	42.6 dB
Antenna beamwidth	1.2°	1.13°

**Figure 10:**
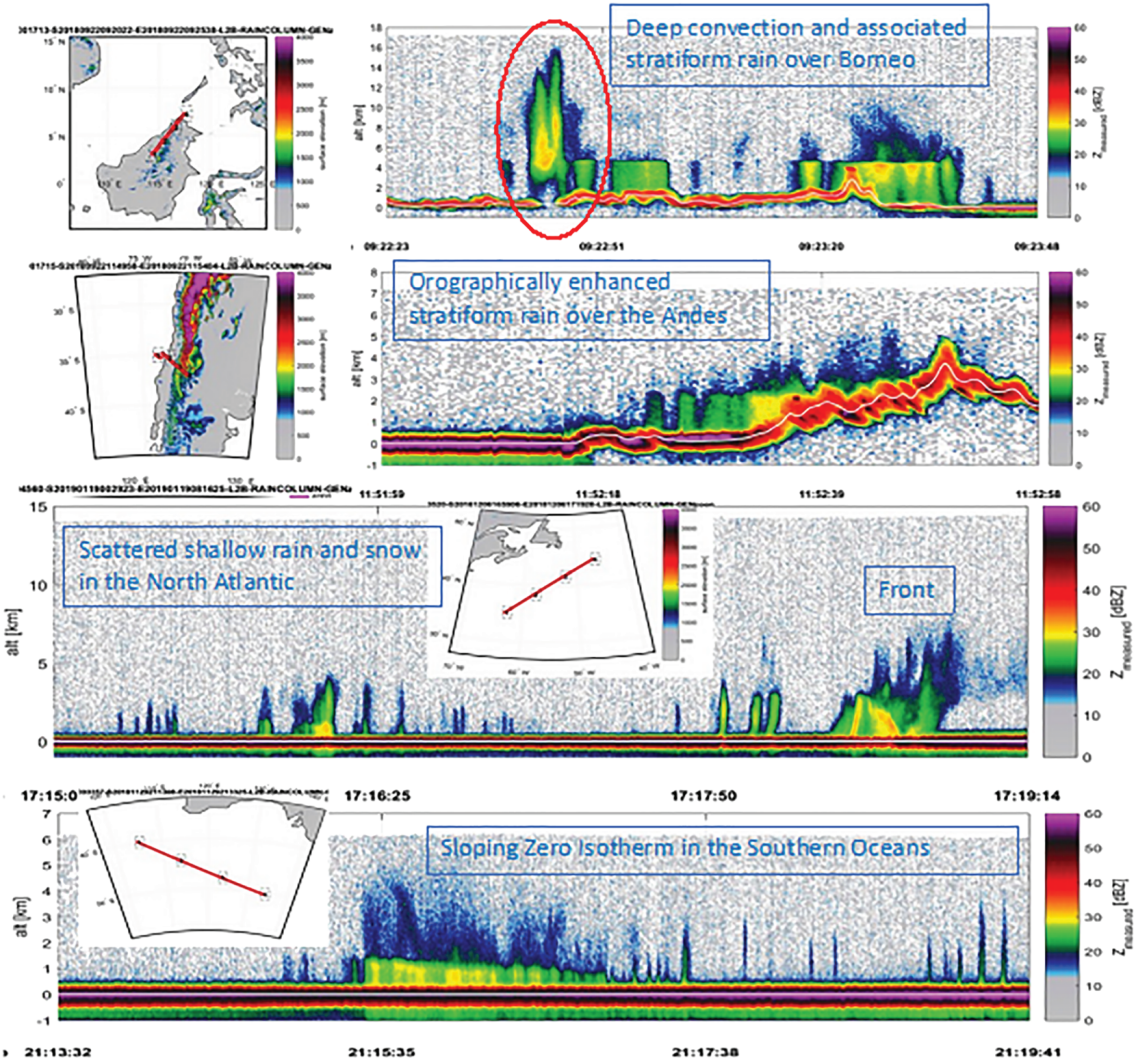
Examples of vertical sections of precipitating systems around the globe observed by RainCube. In all panels the *x* axis represents the along‐track coordinate (see map insets for the specific ground track), and the *y* axis is the altitude above the reference ellipsoid in kilometers. Surface clutter is observed at the expected altitudes, and no range sidelobes contaminate the scene.

A key focus of RainCube is to evaluate the detection of convective systems, which represent a major source of error for forecasts and climate models as they cannot be characterized accurately. In service of the goals of the mission, RainCube has observed various types of precipitation and associated cloud systems. Such targets of interest include stratiform and convective systems, and cover both solid and liquid precipitation. Four examples of RainCube observations (radar reflectivity in dBZ) are shown in Figure 10. The data shown in all panels are consistent with the sensitivity threshold, which has been determined to be 11 dBZ, and the capacity to identify relevant features such as the melting layer of precipitation, or the vertical and horizontal extent of deep convection, are well illustrated by these samples. The effect of attenuation at Ka band is particularly visible in deep convective core shown in the top panel (inside the red ellipse): Contrary to W band, Ka band can profile the entire portion of the deep convective tower above the melting layer; however, it is affected by heavy attenuation in the rain layer below, to the point of losing completely the signal near the surface. The RainCube observations have been verified through direct comparisons to the Ka band observations of GPM DPR: All aspects of performance including sensitivity, resolution, and calibration have been validated. Notably, the quality of these images is testimony to the end‐to‐end quality of the RainCube aggressive pulse compression scheme: The 10 W peak power is transmitted through pulses of 0.166 ms (i.e., equivalent to almost 25 km in range); sensitivity and range resolution are consistent with the compression scheme. In adopting a pulse compression scheme, careful consideration has been placed to reduce range sidelobes to enable rain detection at a minimum altitude comparable to current state of the art spaceborne short pulse radars. While a number of factors including the nature of the surface as well as the instantaneous observing geometry affect greatly the magnitude and extent of the surface clutter signature, in the majority of profiles RainCube can detect rain above the 20 dBZ requirement above 500 m from the surface, and above its effective minimum detectable reflectivity above 1,000 m.

Furthermore, direct application of deconvolution approaches has demonstrated potential to improve the along‐track resolution from the native 8 to 4 km (Tanelli et al., 2019).

### 2.5 EarthCARE

The European Space Agency (ESA), as part of its Earth Explorer Core mission, selected the Earth, Clouds, Aerosols, and Radiation Explorer (EarthCARE) mission with a spaceborne 94 GHz radar and this is intended for launch in 2022 (see Figure 2). The EarthCARE mission is a joint ESA/JAXA venture that aims to better understand the interactions between cloud, radiative and aerosol processes that play a role in climate regulation (Illingworth et al., 2015).

The EarthCARE CPR represents a step forward compared to the CloudSat CPR for the following reasons.

1. It will be the first spaceborne Doppler radar. This will pave the way toward a variety of new observations in climate research ranging from measuring Doppler velocities in the upper part of deep convective cores (Battaglia et al., 2013) to sedimentation velocities in cirrus clouds and stratiform precipitation (Kollias et al., 2014).
2. Thanks to a larger antenna and a lower orbiting altitude, the EarthCARE radar will have an additional 7 dB of sensitivity compared with CloudSat CPR; this will enable the detection of more thin ice clouds and low‐level stratus and stratocumulus clouds (Burns et al., 2016; Lamer et al., 2020). In addition the decrease of the footprint size will reduce the impact of MS (Battaglia & Simmer, 2008) and NUBF effects.

### 2.6 Summary of Characteristics of Current Spaceborne Radars

The aforementioned existing or planned spaceborne radar missions have and will continue to enhance our ability to monitor and study cloud and precipitation processes on a global scale. Different frequencies, antenna types and transceiver technologies have been used in these systems (Table 2). Some notable features of these systems are as follows:

- The Ku, Ka, and W band have each been selected twice as the spaceborne radar frequency.
- The Ku band frequency is currently the lowest radar frequency used in space and provides full‐column characterization of deep precipitating cloud systems.
- The W band frequency is the preferred cloud‐sensing frequency with sufficient sensitivity to detect most radiatively and hydrologically important clouds.
- Higher radar frequencies (e.g., G band and submillimeter wave) have not been used yet but offer an attractive solution for dual‐wavelength measurements at small particle sizes and for water vapor profiling.
- The CloudSat and EarthCARE CPRs achieve high sensitivity and high horizontal resolution by using high‐power Extended Interaction Klystron (EIK) and nadir‐only views of cloud systems.
- Cross‐track scanning is only possible using active‐element phased‐array systems, and mechanical scanning is not practical due to the fast platform motion.
- Vertical resolution of 250 and 500 m has been implemented; however, the surface echo severely limits our ability to detect the lowest kilometer in the atmosphere.
- The EarthCARE CPR is the only Dopplerized radar but is expected to have limited performance in deep convective cloud systems due to strong hydrometeor attenuation.
- RainCube offers several technological breakthroughs (size, deployable antenna, and pulse compression) that should be considered in future spaceborne radar missions.

## 3 Existing Gaps in Cloud and Precipitation Observations

**Figure 11:**
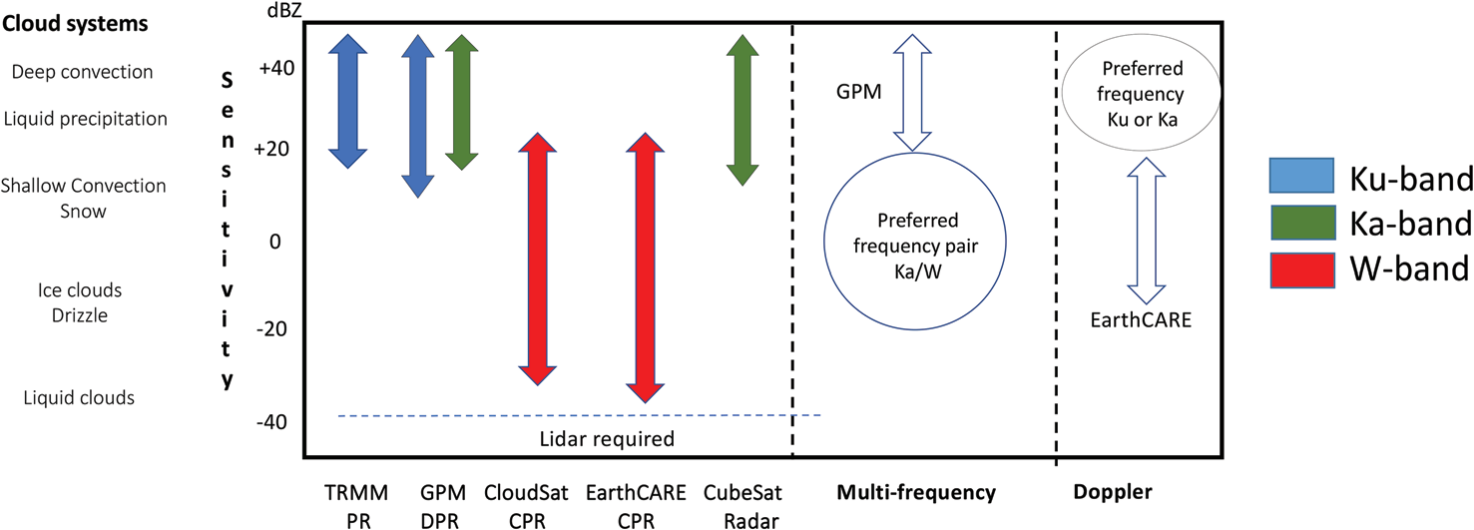
Existing gaps driven by the limited range in sensitivities of the existing and planned radar systems for single‐frequency, multifrequency, and Doppler measurements.

The existing and planned spaceborne radar systems described in section 2 offer a holistic view of the hydrological cycle in action, provide an excellent and stable benchmark for calibration of ground‐based radars and have produced remarkable progress in clouds and precipitation science. Although not on the same satellite, existing and planned spaceborne radars cover the detection of all radiatively and hydrologically important clouds (along with complementary information from spaceborne lidar systems). However, several gaps in the detection and characterization of cloud and precipitation conditions remain, especially when our current limitations in multifrequency and Doppler measurements are considered (as illustrated in Figure 11). The analysis of such gaps started more than a decade ago, in 2007, when the Aerosol/Cloud/Ecosystems (ACE) mission with a payload of a dual‐frequency Ka/W band (35/94 GHz) radar with limited cross‐track scanning at Ka band was recommended as part of the NASA Decadal Survey (The Decadal Survey, 2007). In 2010, another dual‐frequency (35/94 GHz) spaceborne radar concept, the Polar Precipitation Measurement (PPM) mission was proposed to ESA as part of the Earth Explorer Opportunity Mission EE‐8 call (Joe et al., 2010). Although the ACE and PPM missions were not eventually funded, they fostered several conceptual studies and subsystem prototypes that advanced the science and technological readiness. This process culminated in 2017 with the recommendation in the Earth Science Decadal Survey Report (The Decadal Survey, 2017) of a dedicated multifrequency radar‐based mission targeting Aerosols Cloud Convection and Precipitation (ACCP). Besides NASA's new designated ACCP mission, JAXA is currently evaluating different options for a follow‐up to GPM DPR mission and ESA is considering possible synergies between active sensors and their second‐generation polar orbiting meteorological satellites (MetOp‐SG). Furthermore, the explosive growth in the use of CubeSats in Earth Sciences offers ample opportunities for creative approaches on how to best monitor and investigate cloud and precipitation processes (Stephens et al., 2020).

### 3.1 Where Are the Gaps?

The aforementioned efforts to increase our spaceborne radar capabilities aims at a thorough understanding of aerosol‐cloud‐precipitation interactions by shedding light on the “processes” operating within the atmosphere which produce cloud and precipitation systems. Such “processes” include both “microphysical processes” like nucleation and growth mechanisms controlling the evolution of embryonic cloud droplets and ice crystals to precipitation sized particles and involving one (e.g., coalescence) or multiple phases (e.g., melting, riming, evaporation) of the water substance and “dynamical processes” linking 3‐D air motion and cloud structure. These mechanisms should be valid no matter what changes the Earth's circulation will undergo. For instance, accurate measurements of PSDs are fundamental for understanding the processes governing cloud microphysics and improving liquid precipitation representation in numerical models. Especially at small scales, microphysical processes such as condensation of water vapor, collision and coalescence between the droplets, evaporation in unsaturated air, and droplet breakup all contribute to the precipitation that falls at the ground (e.g., Hu & Srivastava, 1995; Seifert, 2008; Tridon et al., 2019). The representation of these processes in Cloud Resolving Models (CRM) ‐which explicitly simulate convective transports with global versions providing simulations at resolutions of about 1 to 5 km, for example, via two‐moment schemes like in Seifert and Beheng (2001); Morrison and Grabowski (2007); Kumjian and Prat (2014); Igel et al. (2015) must be properly assessed. Another critical issue is the coupling between cloud microphysics and storm dynamics, for example, processes like breakup and evaporation can affect the cold pool intensity (Li et al., 2015; Morrison et al., 2012). Feedbacks between dynamic and microphysical processes have significant implications for (1) precipitation rates and their probability distribution functions (including convective‐stratiform partitioning); (2) the horizontal and vertical distribution of clouds (including partitioning between the liquid water and ice phases); and (3) the location and amount of latent energy release associated with phase changes.

These premises underpin a paradigm shift away from our current observing system that largely observe “states” to future observing systems that can capture snapshots of both states and “processes.” Such evolution is critical if advances in the description of cloud/precipitation processes in CRMs and in weather and climate models are sought after. Climate model resolutions are evolving from the order of 100 km (and coarser as in the IPCC, AR5) to 25 km today. Current global operational weather forecasts are performed at or near 5 km, thus approaching convection‐permitting scales. The understanding of these processes is also beneficial for remote sensing retrieval techniques since a better insight of the cloud and precipitation microphysics will automatically better constrain the “atmospheric states” and therefore reduce the uncertainties in the retrieved parameters.

It is important to acknowledge that making progress in our understanding is not straightforward and requires symbiotic use of theory, models and observations and ability to disentangle the role of large scale meteorology and aerosols in cloud‐scale properties and processes. Here, we are concerned only with the advancement needed in spaceborne radar observations to maximize our ability to make progress. There are several areas (processes) of interest for the science community where radars are expected to provide a significant contribution:

1. High‐latitude/midlatitude precipitation,including snowfall (riming, aggregation, and sedimentation);
2. Convective clouds (convective transport and detrainment);
3. Boundary layer clouds (autoconversion and accretion);
4. Mixed phase clouds (Wegener‐Bergeron‐Findeisen process and phase transition);
5. Water vapor in ice clouds and in the column (sublimation and ice nucleation).

In addition to the aforementioned areas where improved measurements are desirable, it is important to state that capturing the diurnal cycle of clouds, convection and precipitation is also a very important issue. The low inclination of the TRMM and GPM missions, combined with their wide swath and their integration with existing passive spaceborne radiometers have allowed the sampling of the diurnal cycle of tropical and midlatitudes convection. On the other hand, CloudSat and EarthCARE follow a sun‐synchronous orbit. Future missions will have to consider architectures that address the need for advanced measurements while providing near‐global coverage and sampling the diurnal cycle in the tropics and midlatitudes. One possible solution of address this requirement is the use of constellations of CubeSats and SmallSats on different orbits. In light of this novel approach we now review some of the gaps in the current cloud and precipitation observing system that could be filled by the new cutting‐edge multifrequency radar systems.

### 3.2 Ice and Snow Microphysics and Ice Processes

High clouds and their properties are often tuned in models to adjust top‐of‐the‐atmosphere energy fluxes; this results in more than an order of magnitude range (from 0.01 to 0.2 kg m^−2^) between global average ice water paths of different climate models (International Panel for Climate Change, 2013). A similar spread exists among remote sensing satellite estimates (Eliasson et al., 2011), which makes it difficult to improve parametrization development for global models. Ice particle fall speed is a very effective tuning parameter for climate models, allowing the top‐of‐atmosphere radiation balance to be adjusted (but not necessarily in a physically consistent way). The sedimentation rate is determined by the PSD. Thus, improved retrievals of the ice and snow PSD and their sedimentation velocity are needed to constrain this parameter. These measurements can lead to improved estimates of different moments of the PSD such as IWC, characteristic size and total number concentration that are critical for evaluating multimoment microphysical schemes (Gettelman et al., 2010). Representing middle‐ to upper‐level ice amounts correctly has significant impacts on cloud‐radiative forcing and, hence, climate implications, with Global Circulation Models typically having biased vertical distributions of ice water contents and producing too much snow over the Arctic and Antartic as well (Palerme et al., 2016). Similarly CRMs tend to incorrectly represent ice microphysical processes; this causes significant inaccuracies in the partitioning between the liquid and ice water species, the depth of the mixed phase cloud region, the vertical redistribution and location of ice and liquid water, and upper‐level detrainment of water vapor.

**Figure 12:**
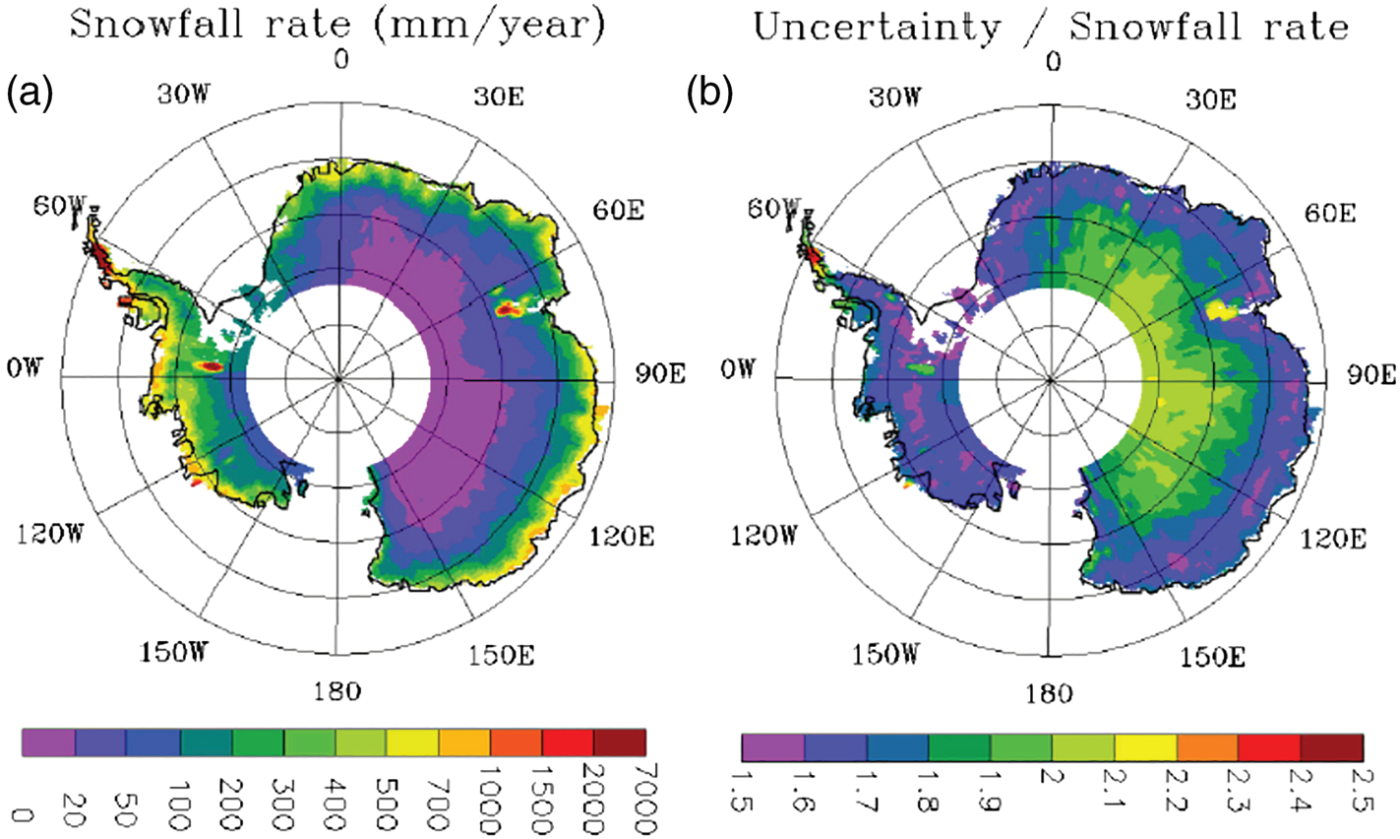
(a) Mean annual snow rate (mm water equivalent/year) derived from the 2C‐SNOW‐PROFILE CloudSat product for the period August 2006 to April 2011. (b) The ratio of the single retrieval uncertainty over the snowfall rate for the same period. Data are accumulated on 1° × 2° grid boxes. Uncertainties include both random and systematic errors. Adapted from Palerme et al. (2014) ©European Geosciences Union. Used with permission.

Detailed quantification of precipitation at the high latitudes is problematic due to the remoteness of these regions and the inability of current spaceborne observations to adequately quantify frozen precipitation. Snowfall is microphysically much more complex to characterize than rainfall. Snowflakes have different shapes, densities (affecting fall velocities) and habits which add complexities to the retrieval problem (Szyrmer et al., 2012; Skofronick‐Jackson et al., 2013). In order to understand the precipitation process, vertical profiling is required to properly quantify the changes from ice formation up in the cloud, to precipitation of snow down to the surface. The CloudSat CPR remains the only active sensor capable of mapping precipitation at high latitudes. However, the snow climatology built by CloudSat has still large uncertainties (Hiley et al., 2011; Palerme et al., 2014): for instance for Antarctica relative uncertainties range between 150 and 250% for 4.7 years accumulated on 1° × 2° grid boxes (see Figure 12). Both CloudSat and EarthCARE offer excellent sensitivity and latitudinal coverage up to 82°N to 82°S but their design is optimized for cloud detection not for precipitation estimates (single‐frequency approach) and their coverage is very limited (nonscanning radars). Furthermore, long pulse durations and lack of pulse compression prohibit these radars from sensing hydrometeors within the lowest km of the atmosphere due to surface clutter. The GPM DPR provides dual‐frequency radar observations only between approximately 66.3°N to 66.3°S. Thus, there is no dual‐wavelength, precipitation‐oriented mission over the high‐latitude regions where solid precipitation dominates. Furthermore, the DPR is insensitive to the light, shallow precipitation that dominates the middle and higher latitudes (Skofronick‐Jackson et al., 2019).

**Observation Gap I: Ability to reliably measure solid precipitation at high‐latitude locations. Accurate measurements of snowfall, snow depth, and rain water equivalent are needed at all time scales and at least daily.**

### 3.3 Liquid Precipitation

**Figure 13:**
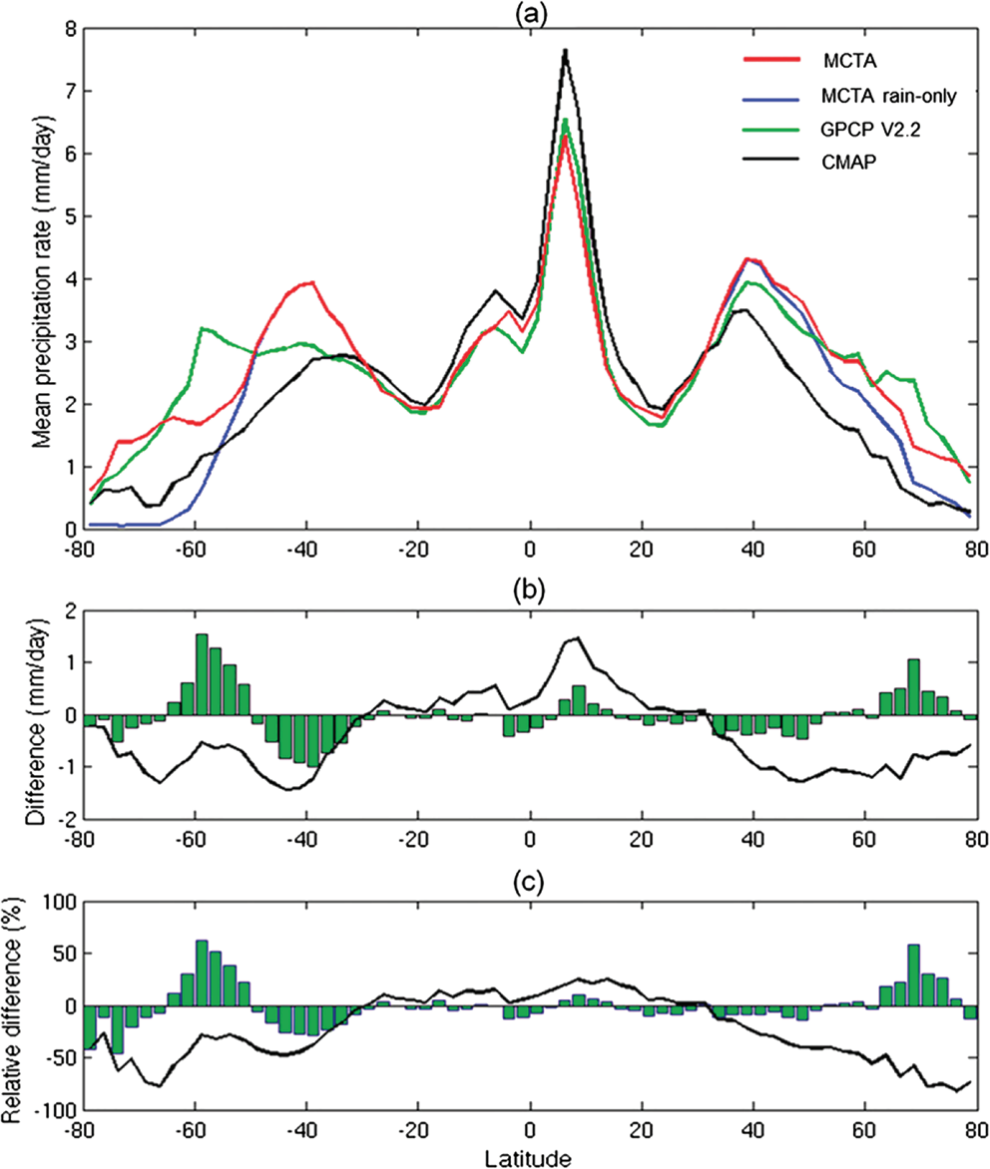
Discrepancies between state‐of‐the‐art global precipitation products and differences compared to MCTA (the merged CloudSat‐TRMM‐Aqua precipitation) estimate. (a) Zonal distribution of mean precipitation rates from the MCTA, Global Precipitation Climatology Project (GPCP), and CMAP. (b) Zonal difference between GPCP and MCTA (green bars) and CMAP and MCTA (solid black line). (c) As in (b) but for zonal relative differences calculated by dividing the zonal precipitation differences of each pair by the mean of the two. Calculations were performed for each 2.5° zonal bin. Extracted from Behrangi et al. (2014) ©American Meteorological Society. Used with permission.

As highlighted by the large uncertainties in the latent heat values (see Figure 1), global precipitation remains poorly quantified. Despite the improvement of spaceborne radar precipitation products via the synergy between the CloudSat and the TRMM radars (Berg et al., 2010; Behrangi et al., 2014), significant differences (Figure 13) still exist when comparing the zonal climatology with state‐of‐the‐art precipitation products derived by combining gauges and geostationary and polar orbiting infrared and MW imagers and sounders with gaps filled in by precipitation forecasts from reanalysis like the Global Precipitation Climatology Project (GPCP,  Huffman et al., 2009) and the Climate Prediction Center (CPC) Merged Analysis of Precipitation (CMAP;  Xie & Arkin, 1997)). Discrepancies are more pronounced in the midlatitudes and high‐latitudes and are particularly profound over the Southern Oceans. The GCOS requirements (total amount at an accuracy of <10% of actual values on monthly time scales on spatial scales of 100 km) are clearly far from reach at the moment. GPM results are promising, but they still show uncertainties similar to those present in Figure 13 (NASA GPM Science Team, 2017) because the GPM‐DPR remains insensitive to the light, shallow precipitation that dominates the middle and higher latitudes. In fact the 35 GHz radar has poorer sensitivity than the Ku itself (see Table 2).

**Observation Gap II: Accurate quantification of precipitation over the midlatitude and high‐latitude regions where large biases between different products exist.**

While TRMM and GPM have provided a great insight into the global distribution of the largest, the deepest and the most intense precipitating systems (Liu & Zipser, 2015; Zipser et al., 2006), there are still large uncertainties in the quantification of extreme precipitation. Based on TRMM products, Hamada et al. (2015) pinpointed at a weak linkage between heaviest rainfall and tallest storms by highlighting the importance of warm‐rain processes and of orographic enhancements in producing extreme rainfall rates. However, caution must be exerted when working with very large precipitation rates (≥50 mm/hr) as derived by current algorithms because in such conditions not only large‐sized particles like raindrops and hailstones are producing non‐Rayleigh effects, but also attenuation, even at Ku (see Figure A6), which can be hard to correct, especially in presence of NUBF effects (Durden & Tanelli, 2008; Kozu & Iguchi, 1999; Short et al., 2015).

**Figure 14:**
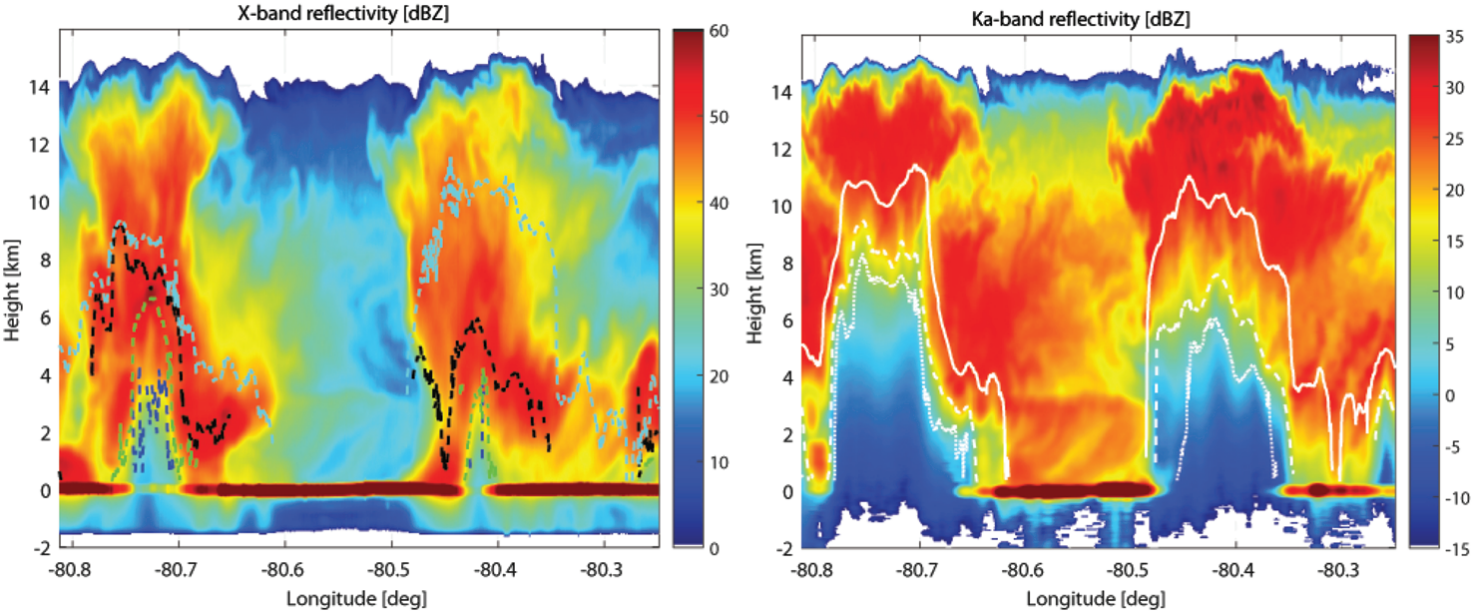
Time‐height reflectivity profiles for an IPHEx overpass over a convective cell at X (left) and Ka (right) bands. In the left panel the different lines (blue, green, black, and cyan) correspond to the level below which the MS contribution becomes predominant at X, Ku, Ka, and W bands, respectively. In the right panel the continuous, dashed, and dotted white lines correspond to the levels at which the top‐down optical thickness exceeds 1, 3, and 5. Extracted from Battaglia, Mroz, Lang, et al. (2016) ©American Geophysical Union. Used with permission.

In addition, when attenuation is caused by highly scattering particles like hailstones in deep convection, MS effects can partially compensate for attenuation and reduce the radar ranging capabilities. When observing storms bearing high‐density frozen hydrometeors such a phenomenon has been proven relevant not only for spaceborne radars (Battaglia & Simmer, 2008; Battaglia et al., 2016, and references therein) but also for air‐borne radar systems (Battaglia et al., 2014; Heymsfield et al., 2013). This is demonstrated in Figure 14, which presents a case study of a convective cell observed by a four‐frequency airborne radar system. Here only X and Ka band radar reflectivities are shown (see Battaglia, Mroz, Lang, et al., 2016, for details), with the latter clearly affected by attenuation but also by MS (e.g., the black dashed line on left panel indicates the level below which MS dominates the radar signal at Ka band). The DPR has several drawbacks when looking at such systems as thoroughly described in Mroz et al. (2018).

**Observation Gap III: Monitoring and quantifying extreme convective events with better estimates of rainfall and detection of hail close to the ground.**

### 3.4 Boundary Layer Clouds and Precipitation Systems

Because of their ubiquitous nature and the way they interact with solar and longwave radiation, boundary layer clouds play a crucial role in the global energy budget (Klein & Hartmann, 1993). Unfortunately, numerical models still struggle to properly represent their coverage, vertical distribution and brightness, for example, Nam et al. (2012), and this uncertainty ultimately affects our confidence in future climate projections (Bony et al., 2015; Sherwood et al., 2014). Improvements to climate simulations could emerge from additional observations of the microphysical and macrophysical properties of clouds as well as from progress in our understanding of the relationships between low‐level clouds and the environment where they develop. In this regard, observations collected by spaceborne sensors are often preferred because they provide global information. The CloudSat‐CPR continues to provide valuable information on high and midlevel clouds (Stephens et al., 2008) but comparisons of various satellite‐based cloud products reveal that it only detects roughly 30–50% of marine stratocumulus clouds globally (Christensen et al., 2013; Liu et al., 2018; Rapp et al., 2013). Although it appears as if spaceborne lidar and infrared sensors could fill this gap, they collect little to no information on the vertical structure of clouds and, as such, cannot provide a comprehensive picture of the radiative properties of low‐level clouds, which also implies that they cannot be used to assess the CloudSat‐CPR's ability to detect cloud base and precipitation.

Ground‐based millimeter radars, on the other hand, are highly sensitive to details in the vertical structure of clouds. Direct comparison between ground‐based and spaceborne observations is challenging because of their differences in coverage; nonetheless, information collected by ground‐based sensors can be used to identify shallow cloud regimes whose properties cannot be captured by spaceborne sensors capabilities. Ground‐based observations indicate that in most regions low‐level clouds form beneath the height at which the CloudSat‐CPR is expected to achieve its nominal sensitivity with minimal contamination from surface clutter (about 1.2 km as reported by Marchand et al. (2008)). For instance, low‐level clouds have been reported to form between 700 and 1,200 m in the Southeastern Pacific region (Bretherton et al., 2010), around 765 m in the central U.S. region (Sengupta et al., 2004) and as low as 400 m in Barrow, Alaska (Dong & Mace, 2003). Thus, in these regimes, only those clouds with considerable thickness, effectively rising above the clutter region, could be detected by the CloudSat‐CPR (Rapp et al., 2013). This also leaves the CloudSat‐CPR with overall little to no ability to record the properties of cloud base. For similar reasons, spaceborne sensors would struggle to assess if low‐level clouds precipitate and if the precipitation they produce evaporates before reaching the surface. According to a study by Yang et al. (2019) precipitation evaporation is not uncommon and as much as 69% of drizzle from single‐layer marine stratocumulus never reaches the surface.

Remote sensors also generally struggle to detect tenuous clouds (Turner et al., 2007). Given the CloudSat‐CPR's minimum detectable signal of −28 dBZ, it should be expected that the CloudSat‐CPR only detects about 5% of cumulus clouds forming over the U.S. continent and only roughly 80% of low‐level clouds forming above 1 km in the eastern North Atlantic (Lamer & Kollias, 2015; Lamer et al., 2020). Also challenging to detect because of their limited vertical and horizontal extent and due to ubiquituous ground clutter and sidelobe return are (1) the lofted stratiform layers identified by Nuijens et al. (2014) as contributing to one third of the low‐level cloud cover in the trade wind region; (2) the forced continental cumulus clouds estimated by Kassianov et al. (2011) to generate net surface cooling over land; (3) shallow, high‐latitude ice/snow cloud systems contributing the majority of precipitation accumulation in the Polar regions (Joe et al., 2010); and (4) orographic precipitation in mountainous regions (Nesbitt & Anders, 2009).

**Figure 15:**
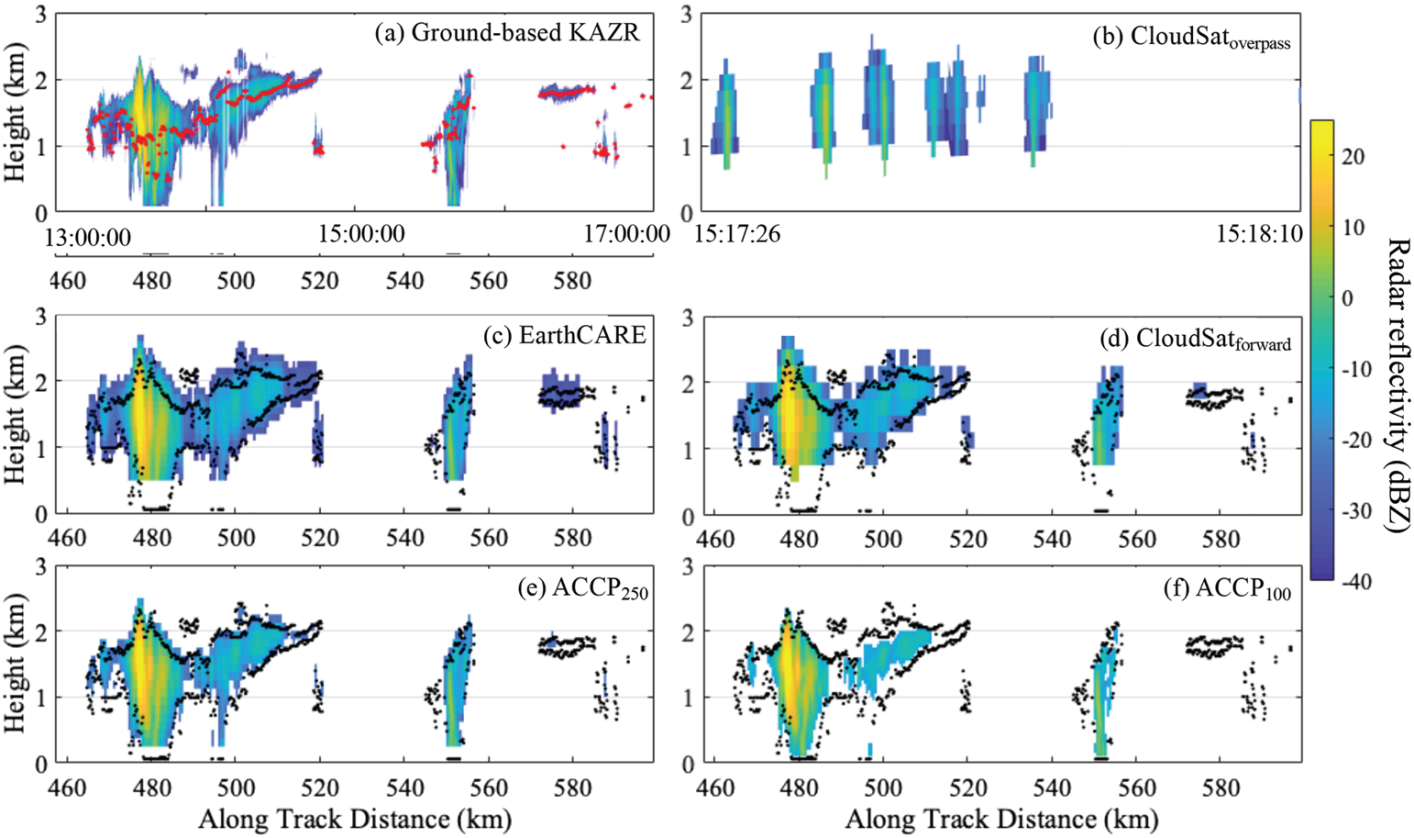
Example of observations collected on 7 December 2015 by Ka band ARM Zenith Radar (a) and by the CloudSat‐CPR (b) when it overpassed within 200 km of Atmospheric Raditation Measurement (ARM) Eastern North Atlantic (ENA) facility. Also displayed are simulations based on the KAZR scene for radar configurations of the (c) EarthCARE‐CPR, (d) CloudSat‐CPR, (e) ACCP‐CPR with a 250 m long pulse, and (f) ACCP‐CPR with a 100 m long pulse. For reference the lidar‐detected lowest liquid cloud base height (red dots) and the KAZR cloud top and cloud/virga base (black dots) are overlaid on these figures.

Illustrating some of these challenges is an example of low‐level cloud observations collected on 7 December 2015 by the CloudSat‐CPR when it passed within 200 km of the Atmospheric Radiation Measurement (ARM) Ka band ARM Zenith Radar (KAZR) located at the Eastern North Atlantic (ENA) facility on the island of Graciosa (Figure 15).

**Observation Gap IV: Clouds and precipitation shallower than 1 km as well as those forming below 1 km and/or in orographically complex regions pose a detection challenge for existing and planned spaceborne radar systems**.

### 3.5 Convective Transport

Deep convection has a profound influence on Earth's climate system for several reasons. Updraft plumes in deep convective clouds (hot towers;  Riehl & Malkus, 1958) are the principal pathway by which heat, moisture, mass and trace gases are transported in the tropics into the upper atmosphere. Observations of the occurrence and magnitude of vertical transport in deep convection over the tropical oceans are simply not available and sparsely available over land (Cotton et al., 1995; Kumar et al., 2016, 2015). Monthly to seasonal prediction of weather is heavily influenced by the role of deep convection on important modes of variability, such as the Madden‐Julian Oscillation, El Niño/Southern Oscillation, and tropical waves. Convection is therefore central to prediction of severe weather and prediction on subseasonal and seasonal time scales (Bechtold et al., 2008).

**Figure 16:**
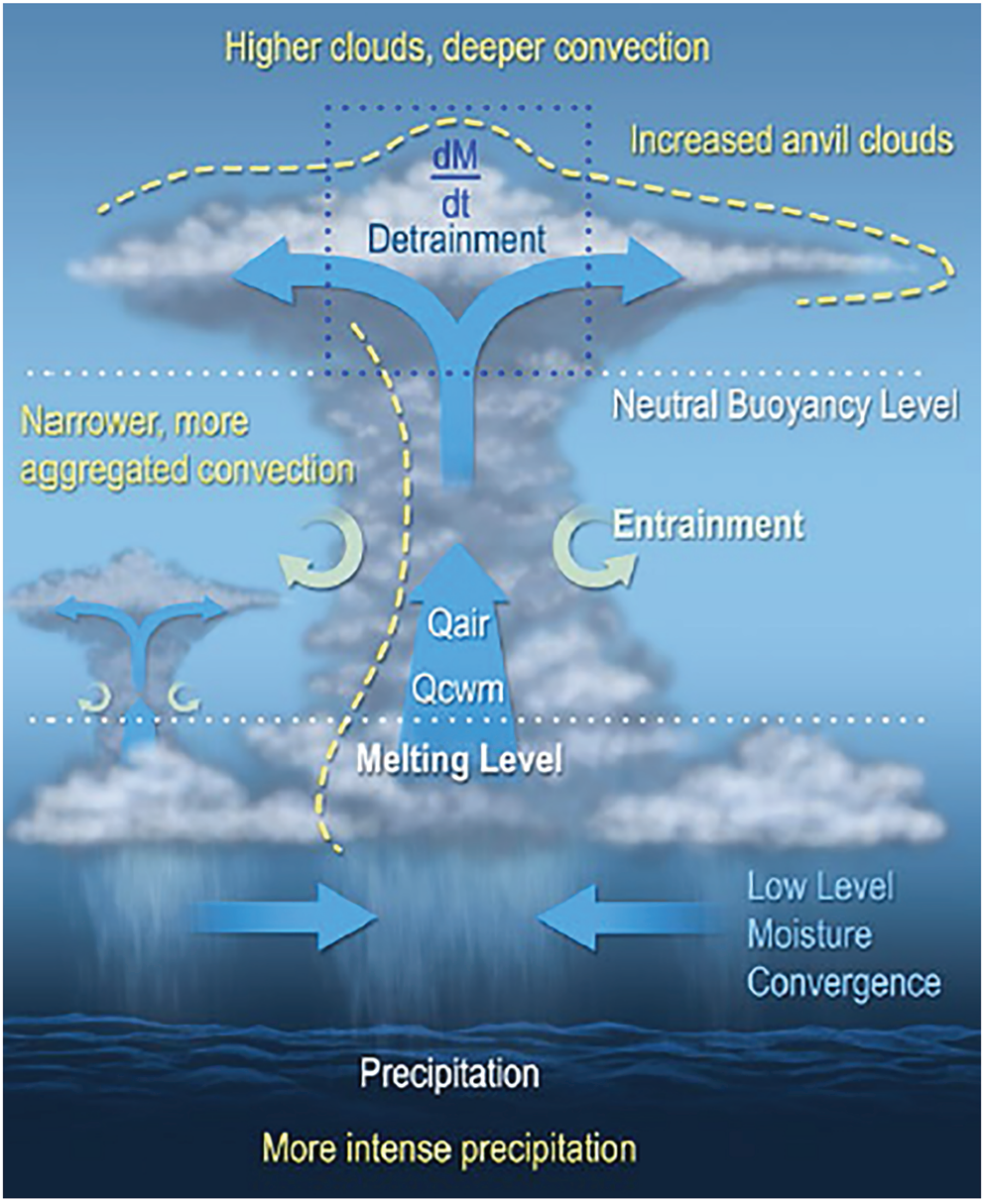
A conceptual view of the transports of air and condensed water mass (*C* *W* *M*) by deep convection. Observations of vertical profiles of air and condensed water mass fluxes (*Q*_*air*_ and *Q*_*CWM*_, respectively) and of the temporal evolution of these quantities (*d* *M*/*d* *t*) could give unique and direct insight into convective transport (courtesy of G. Stephens and D‐Train team).

The sign and nature of changes to convective storms in a warming climate are also difficult to predict (see yellow notes in Figure 16). While moisture convergence is expected to increase at about 7% per degree of warming following the Clausius‐Clapeyron law, storms are likely to become deeper and narrower and toproduce heavier precipitation at a rate which is still debated (Mauritsen & Stevens, 2015). It also remains unclear whether or not the increased transports will result in more moistening of the high troposphere and more cirrus clouds or will be compensated by heavier precipitation (D‐Train Science Team, 2016).

The Panel on “Weather and Air Quality: Minutes to Subseasonal” of the NASA Decadal Survey has identified Convection and Heavy Precipitation as central theme. Specifically “Why do convective storms, heavy precipitation, and clouds occur exactly when and where they do?” has been listed as one of their four most important Science and Application Questions. The panel also suggests that “to improve the prediction of convective processes, observations of the various physical mechanisms within the clouds and local environment that act to produce precipitation are needed. This includes the cloud microphysical properties and the vertical motions within convective storms that are associated with heavy precipitation. These process‐oriented measurements should enable new insights to inform the next generation of cloud and precipitation models for weather forecasting. It is imperative that these measurements constrain and define these processes that are critical for more accurate weather forecasts and predictions of the water cycle.”

**Figure 17:**
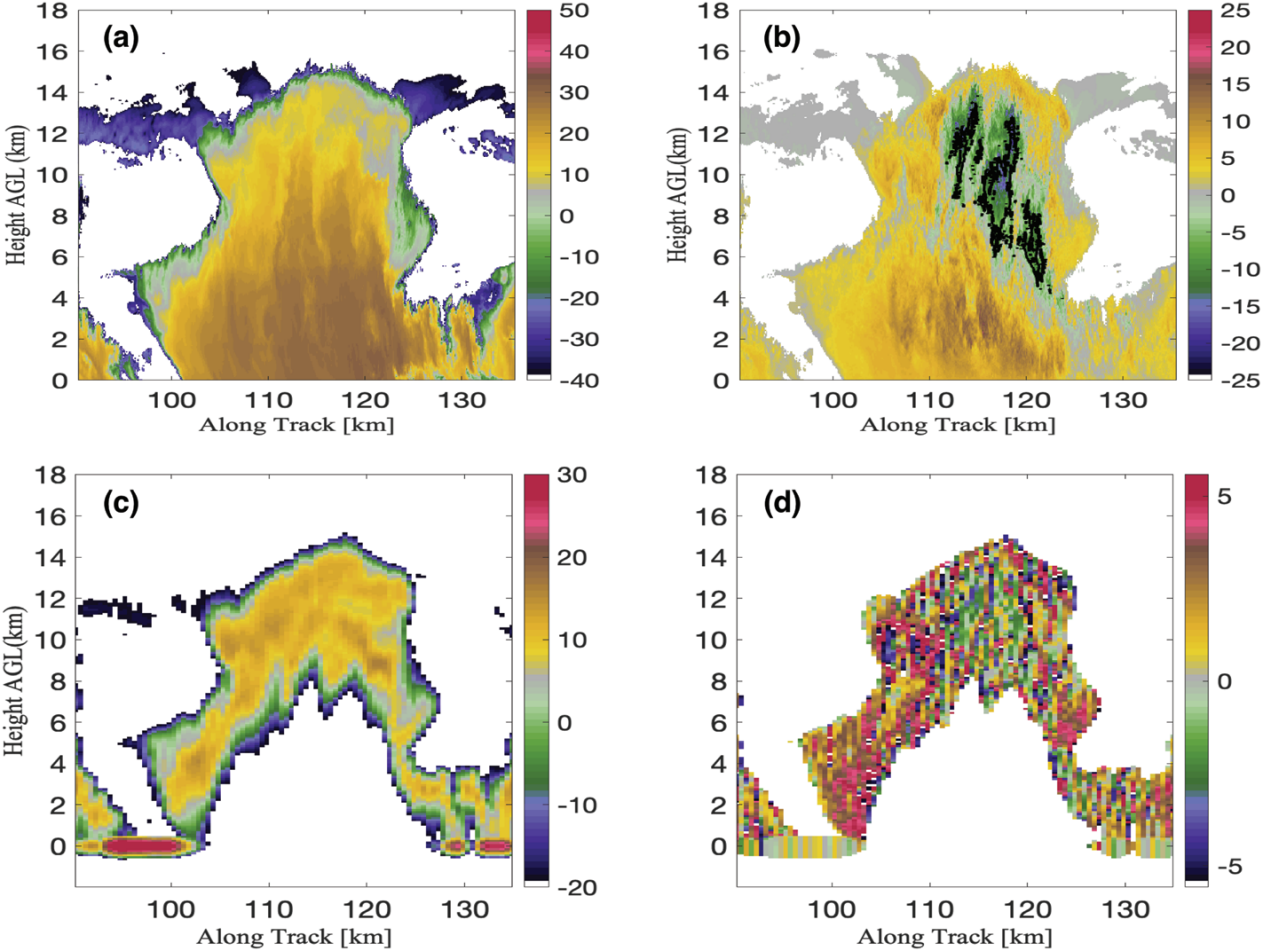
A simulated oceanic deep convective cloud using the System for Atmospheric Modeling (SAM) model (Khairoutdinov & Randall, 2003) at a 50 m horizontal and vertical resolution. The SAM model simulation was initialized using the Idealized Global Atmospheric Research Program's Atlantic Tropical Experiment (GATE) simulations of convection over the tropical Atlantic setup (Xu & Randall, 2001). The four panels indicate (a) nonattenuated 94 GHz radar reflectivity factor, (b) nonattenuated 94 GHz mean Doppler velocity, (c) attenuated 94 GHz radar reflectivity factor, and (d) simulated Doppler velocity from the EarthCARE CPR.

Arguably, spaceborne radar observations are the only way to sample deep convective clouds over the vast tropical oceans. However, estimating the vertical air motion (±20 – 30 m s^−1^) with an accuracy of 2 m s^−1^ from a platform that moves at about 7,600 m s^−1^ is a daunting task (Battaglia et al., 2013; Kollias et al., 2014, 2018; Sy et al., 2014; Tanelli et al., 2002). When launched (currently scheduled for 2022), the EarthCARE CPR will be the first Dopplerized spaceborne radar (Illingworth et al., 2015). Due to the instrument configuration and limitations, the EarthCARE CPR Doppler velocity measurements are expected to be very challenging (see section 4.6 for details). This is especially true in deep convective clouds due to significant hydrometeor attenuation, MS, NUBF conditions and velocity aliasing (Figure 17). A detailed evaluation of the expected performance of the EarthCARE CPR in deep convection is provided by Kollias et al. (2018). The CPR is expected to adequately measure Doppler velocity in weak convection (moderate ice/graupel amount and updraft magnitude less than 6–7 m s^−1^). In stronger convection, as for the case depicted in Figure 17, the CPR is expected to have limited penetration to the upper one third of the convective core (Figure 17c) and the Doppler velocity measurements will be practically unusable due to the low Nyquist Doppler velocity of the CPR (5–6 m s^−1^) and the uncertainty of the Doppler velocity measurements (about 1–2 m s^−1^) that will make the retrieval of the original Doppler velocity field (Figure 17b) from the observed (Figure 17d) very challenging.

Thus, there is a remaining gap in observing the vertical velocity in convective systems. The gap is not limited to over the oceans but also occurs over land. Recent studies (Oue et al., 2019) have demonstrated that the use of multi‐Doppler radar techniques to retrieve the vertical air motion in deep convection suffer from large uncertainties above 5–6 km altitude due to the sparse radar sampling in these levels and the slow sampling time (5–6 min) that is not rapid enough to sample transient, fast‐evolving updraft and downdraft structures.

**Observation Gap V: There are currently no wide‐scale observations from any of the existing Earth‐orbiting satellites to test model representations of deep convective transports. Observations of vertical motion and of joint measurements of vertical velocity, water content, and microphysics are needed for the evaluation of processes associated to vertical latent heat distribution and convective mass flux.**

### 3.6 In‐Cloud Water Vapor Sounding

Accurate knowledge of the atmospheric water vapor distribution is a critical ingredient in both climate and numerical weather models, as its radiative forcing and thermodynamic effects help determine the Earth's energy balance and drive atmospheric dynamics across many scales (Held & Soden, 2000; Stevens et al., 2017; Wulfmeyer et al., 2015). Unfortunately, existing spaceborne humidity sensors, including passive microwave (e.g., the Advanced Microwave Scanning Radiometer, or AMSR) and infrared (e.g., the Atmospheric Infrared Sounder, or AIRS) systems, are incapable of producing reliable humidity estimates with high spatial resolution inside of clouds and precipitation, as microwave sounders resolve only a few (≤3) vertical levels and are biased by precipitation, and infrared humidity retrievals are limited to areas with clear sky or optically thin clouds. Furthermore, coverage from passive microwave sounders is typically limited to ice‐free ocean surfaces due to uncertainties in land surface emissivities. While Global Navigation Satellite System (GNSS) radio occultation techniques are less susceptible to biases from clouds and precipitation, they have coarse horizontal resolution (∼⃒100 km) and thus cannot capture the horizontal water vapor gradients that help drive weather phenomena. These limitations are recognized by the NWP community, with the World Meteorological Organization repeatedly highlighting that “a critical atmospheric variable that is not adequately measured by current or planned systems is vertically resolved humidity in cloudy areas” (Anderson, 2018).

While the ideal humidity measurement is one that captures its three‐dimensional variability with high horizontal and vertical resolution, the vertically integrated, or total column water vapor (TCWV) has been shown to be a particularly useful quantity tied to cloud and precipitation formation (Bretherton et al., 2004). Operational weather models (Anderson, 2018) and reanalysis studies (Dee et al., 2011) assimilate TCWV from microwave imager observations, including both direct assimilation of retrieved TCWV (Gérard & Saunders, 1999) and indirect assimilation by comparing modeled versus measured radiances. However, as discussed above, passive TCWV sampling is typically limited to nonprecipitating, ice‐free ocean regions, though in the case of ERA‐Interim precipitating regions over ocean are not excluded (Dee et al., 2011). To extend the analysis into regions with precipitation, the effect of rainfall on measured radiances must be modeled given condensation and convection parametrizations and assumptions about the hydrometeor distributions, making the inferred TCWV in these regions susceptible to biases.

**Figure 18:**
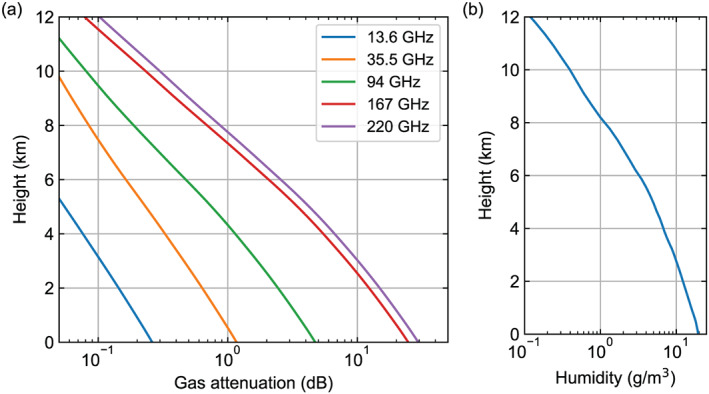
(a) Integrated attenuation from the top of the atmosphere in a moist tropical setting for various cloud and precipitation radar frequencies. Gas extinction coefficients are calculated using the Microwave Limb Sounder (MLS) millimeter‐wave propagation model (Read et al., 2004). (b) Vertical humidity profile used to calculate integrated attenuation.

In the context of multifrequency radar observations of clouds and precipitation, inaccurate knowledge of the water vapor distribution along the radar beam's line of sight presents an important limitation to quantification of the microphysical properties, especially for W and G band systems. Figure 18a shows the integrated two‐way, top‐down attenuation due to atmospheric gases of various precipitation and cloud radar frequencies in a moist tropical atmosphere. Below 8 km for multifrequency systems that include G band, the DFR attenuation effect (see Equation A8) stemming from gas absorption exceeds 1 dB even for *window frequencies*, increasing to much higher values of 20–30 dB in the boundary layer. Even for W band the lower tropospheric attenuation effect approaches 5 dB, which has clear consequences for spaceborne observations of liquid clouds and precipitation. Because the integrated attenuation is very nearly linear in the absolute humidity, and because humidity is highly variable in both space and time, it is critical to have reliable estimates of water vapor profiles along the beam path in order to mitigate biases in the attenuation effect term for multifrequency retrievals.

**Figure 19:**
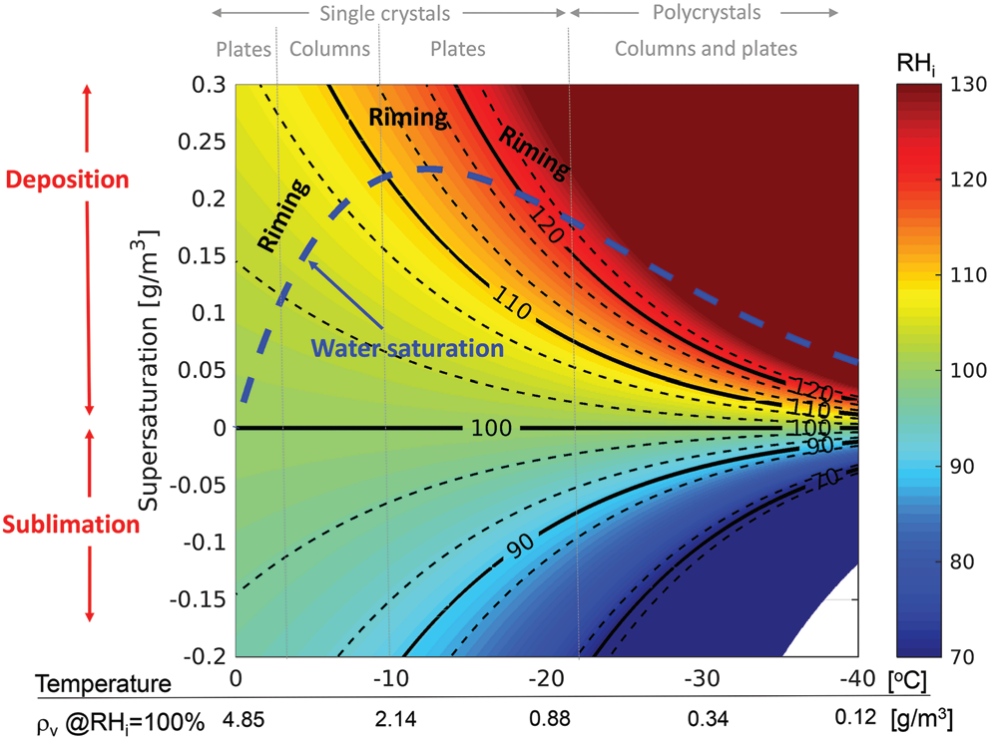
Temperature in °C versus supersaturation in g/m^3^ with highlighted regions where riming, deposition, and sublimation processes are active. The color maps the relative humidity with respect to ice, *R* *H*_*i*_. The dashed blue line indicates the supersaturation of supercooled water relative to ice. Black lines correspond to different levels of *R* *H*_*i*_ as indicated by the labels. The dashed lines surrounding each continuous line correspond to a ±3% change in *R* *H*_*i*_. Some of the dominant ice crystal habits as suggested by Bailey and Hallett (2009) for different environmental conditions are indicated at the top of the figure. Adapted from Battaglia and Kollias (2019) ©European Geosciences Union. Used with permission.

In addition to its importance for correcting DFRs for gas attenuation prior to evaluation of various scattering models, knowledge of the ambient water vapor field within clouds is of interest for microphysical process studies. For instance, there are three major processes that govern the growth of ice particles: vapor deposition/sublimation, aggregation, and riming. As shown in Figure 19, knowledge of relative humidity and temperature within ice clouds could help to discriminate the types of crystals present and degree of riming occurring. This could narrow down the selection of ice habits populating a given part of the cloud and help identify regions where particular microphysical processes are likely to occur. Such information would greatly reduce uncertainty in the multifrequency retrievals because, without it, the particular habit type and amount of riming are fairly unconstrained model parameters. Additionally, identification of the dominant process active in a segment of cloud is critical to improving model parameterizations (e.g.,  Wilkinson et al., 2010).

**Observation Gap VI: Highly resolved observations of vertical profiles of water vapor in cloudy conditions.**

## 4 Future Outlook

### 4.1 Advances in Technology

**Figure 20:**
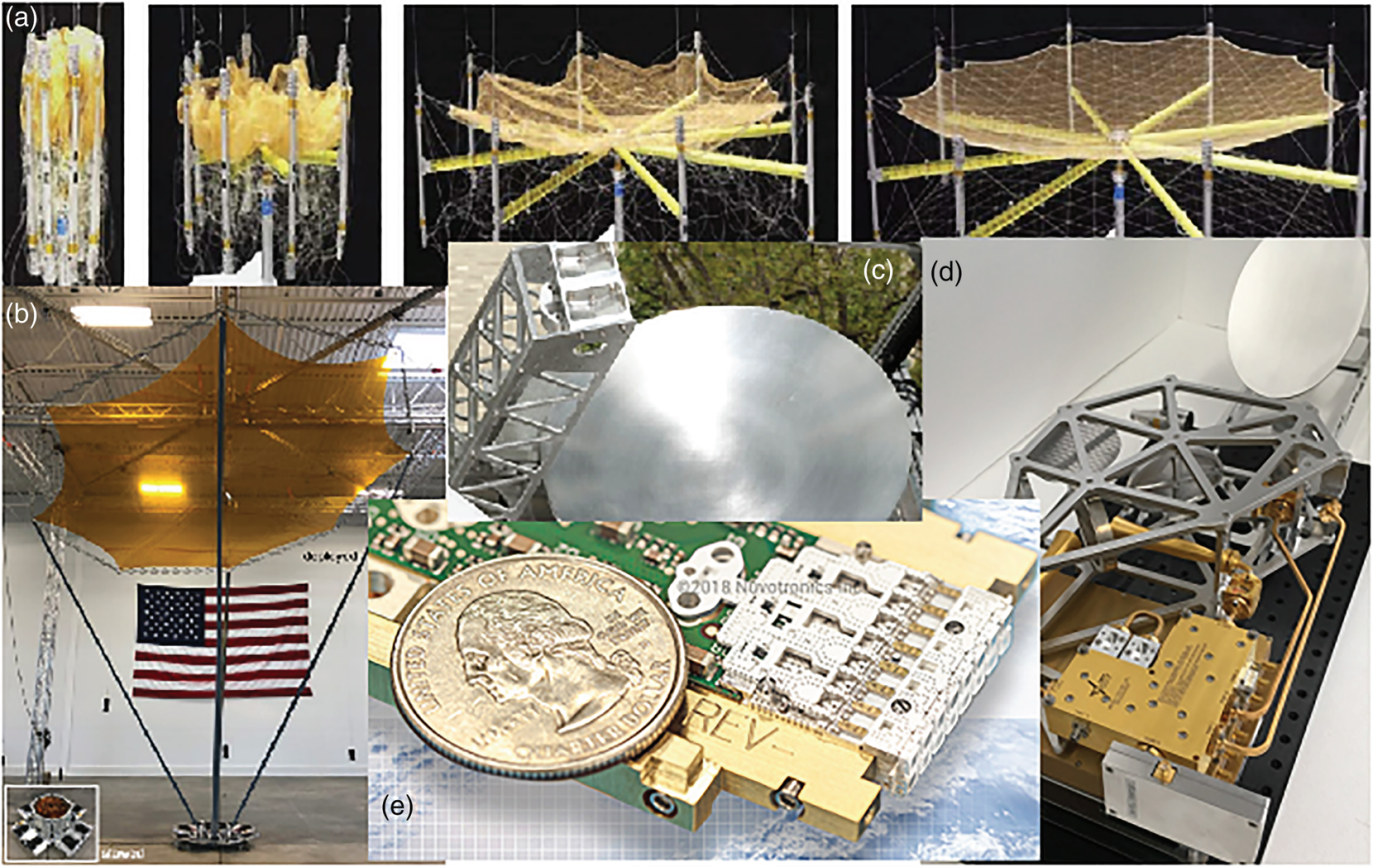
Examples of recent technology developments enabling cloud and precipitation radar concepts. (a) Mesh deployable antennas at Ka band and lower frequencies (https://85f2c62a-b345-48a7-8394-fe93e1395d10.filesusr.com/ugd/c5273f_0081c8a108f5424683ac6fd36d0025fe.pdf). (b) Membrane deployable antenna at X band (Cooley et al., 2019). (c) The G band VIPR (Vapor In‐cloud Profiling Radar) antenna system. (d) The GAISR W band FMCW radar (Cooper et al., 2020). (e) W band electronically scanning line feed array for cloud and precipitation (https://www.nuvotronics.com/antenna_array.php).

Significant radar advancements relevant to the NASA Aerosols Cloud and Ecosystem (ACE, 2007) and Aerosols Cloud Convection and Precipitation (ACCP, 2017) Earth Science Decadal Survey observing system concepts have been achieved in the last two decades. These advancements were enabled by various NASA technology investments and airborne field campaign activities and leveraged substantially on the rapid general progression of digital and small platform technologies. Overall, these mission concepts provided the necessary focus for technological advances specifically targeting the observation of clouds, convection and precipitation. Remarkable advances in component and subsystem technologies that are directly relevant to spaceborne cloud and precipitation radars have occurred in the last decade and continue to progress. At the core of new capabilities such as RainCube shown in Figure 9, ubiquitous advancements in digital technology (almost entirely enabled by needs in commercial electronics) are front and center to all current radar concepts since they enable digital waveform generation and signal processing, low sidelobe pulse compression and compact and low power radar architectures. Another area that has seen critical improvements is that of lightweight deployable antennas (e.g., mesh and membrane) and ultra compact antennas (e.g., metasurface and reflectarray), at centimeter and millimeter waves (see comprehensive reviews in Chahat, Decrossas, et al., 2019; Chahat, Sauder, et al. 2019, and; Rahmat‐Samii et al., 2019). Similarly, advancements in solid state power amplifiers and low noise amplifiers at millimeter and submillimeter wave (on GaAs, GaN, InP, and SiGe substrates), as well as power combination and compact vacuum electron devices, have reached or are reaching levels of performance that make them directly applicable to state of the art radar concepts. These technologies continue to improve and enable the development of spaceborne radars with either a large aperture antenna for a conventional satellite platform or with a compact deployable antenna for CubeSat/SmallSat application. Figure 20 provides a noncomprehensive visual summary of products of recent technology innovation directly relevant to spaceborne Cloud and Precipitation Radars; see Peral et al. (2018) for a review on this topic.

Since the launches of TRMM's Ku band PR in 1997 and CloudSat's W band CPR in 2004, two main avenues of advancement have been pursued to meet the stated or perceived needs of the science community: improved performance and reduced SWaP (Size, Weight and Power) /data rate, thus ultimately mission cost. Along the first, the DPR was developed by pairing slotted waveguide Ku band and Ka band electronic scanning, launched in 2014 and has been successfully operating in orbit since then. Also, the Cloud Profiling Radar (Illingworth et al., 2015) under development for the EarthCARE mission adopts technologies similar to CloudSat's CPR but augmented to provide the first ever cloud Doppler measurements from space.

Similarly, as soon as the ACE mission concept began being developed (following the 2007 Earth Science Decadal Survey), four specific additional needs with respect to the CloudSat baseline became the primary drivers of new radar concepts: combination of W band with one or more lower frequencies, introduction of at least a limited scanning capability, introduction of Doppler capability, and achievement of higher quality data sets (i.e., smaller horizontal and vertical resolution, improved radar sensitivity and reduction of the vertical extent of surface clutter contamination). The first possible solution to meet such needs was the Radar for the ACE mission (ACERAD) technology development where the CloudSat‐class technology was augmented to obtain additional Ka band measurements over a limited swath of 30 km. The key technology developments implemented to enable this concept were the Dragonian antenna design (to allow Ka band scanning), the Dual‐Frequency Dual‐Polarization Quasi‐Optical transmission line, the Ka/W band frequency selective surface, and the signal generation and processing strategy (Durden et al., 2016). The primary limitations of this technology are in the marginal potential for further miniaturization (because the space‐qualified Vacuum Electron Device high power amplifiers of this class, and because of the simple waveforms adopted which impose large antenna sizes). In a broad analogy to the TRMM/PR and GPM/DPR precipitation radars produced by JAXA/NICT in the first decade of this millennium, these are mature, proven and reliable radar technologies, which, however, require significant allocations in SWaP and are therefore difficult to scale‐up without significantly impacting other mission costs.

In order to enable instrument performance closer to the scientific needs expressed during the definition of ACE, two additional instrument concepts were defined: Wide‐swath Shared‐aperture Cloud Radar (WiSCR) and Three‐band (Ku/Ka/W band) Cloud and Precipitation Radar (3CPR). Both include use of active electronically scanning linear arrays (AESLA) illuminating a singly‐curved parabolic reflector (SCPR) to increase the radar cross‐track scanning capabilities without incurring the challenges and costs associated with large 2‐D active arrays. Both concepts adopt advanced signal generation and processing schemes to achieve the desired radar sensitivities, resolutions and Doppler accuracies. In WiSCR (Hand et al., 2013; Racette et al., 2011) an AESLA for Ka band is combined to a W/Ka band reflectarray main reflector to enable use of CloudSat heritage technology at W band. The reflectarray technology enables collocated beams for all frequency bands with capability to support either fixed nadir or scanning W band beams, and wide swath at Ka band. The technological development of 3CPR concept (Sadowy et al., 2016) hinges upon proven Ku and Ka band AESLA technologies and matures an innovative W band active feed array technology to enable scanning at all frequencies (see Figure 20e). The key to this technology lies in an interleaved pattern of transmit and receive radiative surfaces that circumvents the need for any T/R switches, and on the modular development which facilitates design and implementation of AESLA of arbitrary length by mating them alongside in the scanning plane. Also following the goal of augmenting the capabilities, radar concept development efforts in Japan have focused on satisfying the JAXA's “Grand Plan for Satellite Observation on Water Cycle,” including needs of JAXA's Global Satellite Map of Precipitation (GSMaP). The first approach is to upgrade GPM/DPR. Even with existing technology, it is possible to expand the swath width (i.e., ∼⃒350 km) by about 1.4 times than GPM/DPR and gain the sensitivity (i.e., ∼⃒0 dBZ) by about 63 times (18 dB) than GPM/DPR. On the Doppler velocity measurement, a larger antenna must be considered and is being studied within the context of the KuPR‐2 concept to contribute to ACCP.

The advancements described above focused on delivering additional capabilities while managing an increase in SWaP. The last decade has also seen a number of efforts aiming at delivering spaceborne cloud and precipitation radars that primarily reduce the SWaP while managing the expected performance within bounds that are still of significance to the science community. This line of development is largely enabled by the significant improvements in access‐to‐space (be it via an accommodation on the International Space Station or on a small‐platform) and state‐of‐the‐art digital technologies. These include the design of radars composed where possible of commercial off‐the‐shelf electronic parts, and advanced waveforms. Among these, one radar that transitioned from early concept to launch and successful mission completion in less than 7 years is the Ka band RainCube, launched in 2018 and still operational at the time of writing (Peral et al., 2019, and section 2.4) RainCube enables mission concepts (Stephens et al., 2020) involving a number of small platforms in Low Earth Orbit with small downward looking radars, complementing a similar constellation of small microwave radiometers in a fashion similar to GPM. These small platforms can be arranged in trains (Stephens et al., 2020) along one orbital plane (to capture the short time scale evolution) and/or on different orbital planes (to improve the sampling of the diurnal cycle). RainCube demonstrated (a) an ultracompact back end architecture (which includes the digital system and the up/down conversion units, performing real time ultralow range sidelobe pulse compression, with direct modulation and demodulation between baseband and Ka band) which could be inherited by any future cloud and precipitation radars (at any wavelength), hence reducing the size, weight and power of the digital subsystem and up/down conversion assemblies); (b) the specific waveform and filtering for pulse compression (designed keeping in mind the ACE radar requirement of confining the ground clutter to only 500 m above the surface); and (c) one first version of an ultracompact lightweight deployable 0.5 m Ka band antenna for radar applications (see Figure 9). Another significant achievement of this technology demonstration lies in the fact that RainCube adopted commercial off‐the‐shelf parts in most of its subsystems, favoring selective use of radiation hardened parts only in particularly critical subsystems, and redundancy approaches in the firmware.

A portion of the technology developed for RainCube (that is, the back‐end architecture) has already been integrated in the Multi‐Application Small‐satellite Tri‐band Radar whose airborne prototype is currently under development. In essence, this instrument concept unifies the 3CPR front end technologies described earlier (i.e., Ku, Ka, and W band Active Line Array Feeds and singly curved parabolic reflector) with the RainCube signal architecture to deliver an instrument that can address both the cloud and precipitation measurements as well as innovative altimetric measurements focusing on sea ice freeboard and the thickness of the snowpack above it, snowpack over ground, or, if installed in a spinning scanning platform, scatterometric measurements for ocean surface winds. Because of the miniaturized nature of each subsystem, and the modular scalability of the Active Line Array Feeds, this instrument concept can be scaled to antenna sizes ranging from 0.3 to 3 m, making it suitable for a variety of accommodations according to specific instrument performance and SWaP allocations (ranging from 6U cubesats, to buses capable to accommodate instruments of a few 100 kg mass and requiring in the order of 1,000 W), and including any or all of the three bands.

A second direct and natural descendant of RainCube, under development at the time of writing, aims at merging the Ka band channel (augmented with a small swath capability) with similar nonscanning W band and G band channels. It also integrates the capability to adopt either a single antenna or a pair of antennas (the second one being a lightweight deployable mesh of the type shown, for example, in Figure 20a) to obtain Doppler measurements via the Displaced Phase Center Antenna approach (Durden et al., 2007; Tanelli et al., 2016) with a target performance of 0.5 m s^−1^ accuracy and +5 and ‐20 dBZ radar sensitivity at Ka and W band, respectively. More generally, the RainCube architecture enables a number of possibilities for small platforms, at a number of frequency bands spanning from X to G band. Several other compact radar concepts have recently been formulated or are under early development stages. They target primarily the evolving needs of the ACCP observing system concept. Among them, some target lower frequencies (such as X or Ku band) for improved penetration capability in deep convection and heavy precipitation and to leverage on mature electronics and large deployable antenna technologies. The general viability of this approach is well represented by examples such as the DARPA's Radio Frequency Risk Reduction Deployment Demonstration (R3D2) satellite which was launched with a less than 2 year development time using commercial parts and low cost (see Figure 20b and Cooley et al., 2019) and which is being leveraged upon for a precipitation Doppler radar concept with a 5 m deployable antenna targeting a 1 m s^−1^ Doppler accuracy and 5 dBZ sensitivity. Along similar lines, a small radar constellation concept is being developed in Japan to address the needs of GSMaP: It consists of small size TRMM/PR‐like precipitation radar satellites to provide 6‐hourly precipitation map over tropical region.

One of the ultimate goals of the precipitation radar would be the precipitation observation from geostationary orbit. Such vantage point grants two important benefits: continuous observation over a large region of Earth (in the low to midlatitudes) with resampling times in the order of minutes and small platform velocity (relative to Earth's surface) to facilitate Doppler measurements. Because of the significant distance from Earth, the required technological advancement is the large size (about 30 m in diameter for Ka band) antenna which has been subject of technology development efforts in the first decade of the millennium (e.g.,  Lewis et al., 2011), and may be possible in near future.

### 4.2 A Multifrequency Approach to Target the Science Gaps

**Figure 21:**
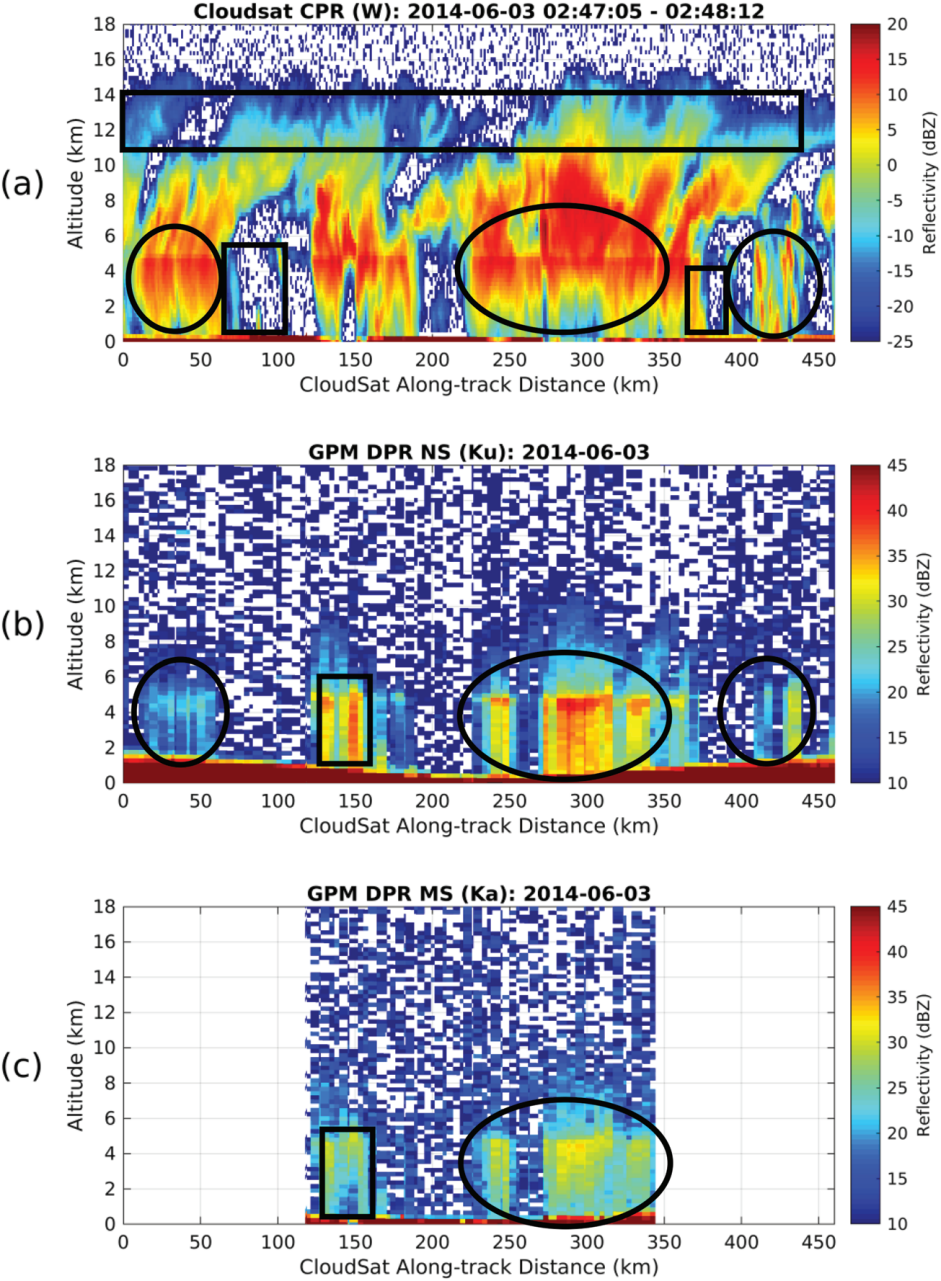
Collocated CloudSat and GPM reflectivity data with CloudSat Cloud Profiling Radar W band (a), GPM Normal Scan Ku band (b), and GPM Matched Scan Ka band data (c) for a precipitating system over the tropical Pacific Ocean (latitude: 4.6°N to 8.7°N, longitude: 163.4°E to 164.3°E) in the summer of 2014. The black rectangles (circles) identify regions where the different radar systems are complementary (synergistic). There is a ∼⃒5 min gap between the two satellite observations.

The potential of multifrequency spaceborne radar observations is clearly illustrated in Figure 21, which shows a multifrequency observation of the same precipitating system with three frequencies ranging from 13 to 94 GHz. The case is extracted from the combined CloudSat/GPM data product, which provides data from coincident CloudSat and GPM (see section 2.2) satellite overpasses, with GPM data being mapped onto the CloudSat satellite track.

Two aspects of the multifrequency approach are apparent.

**Figure 22:**
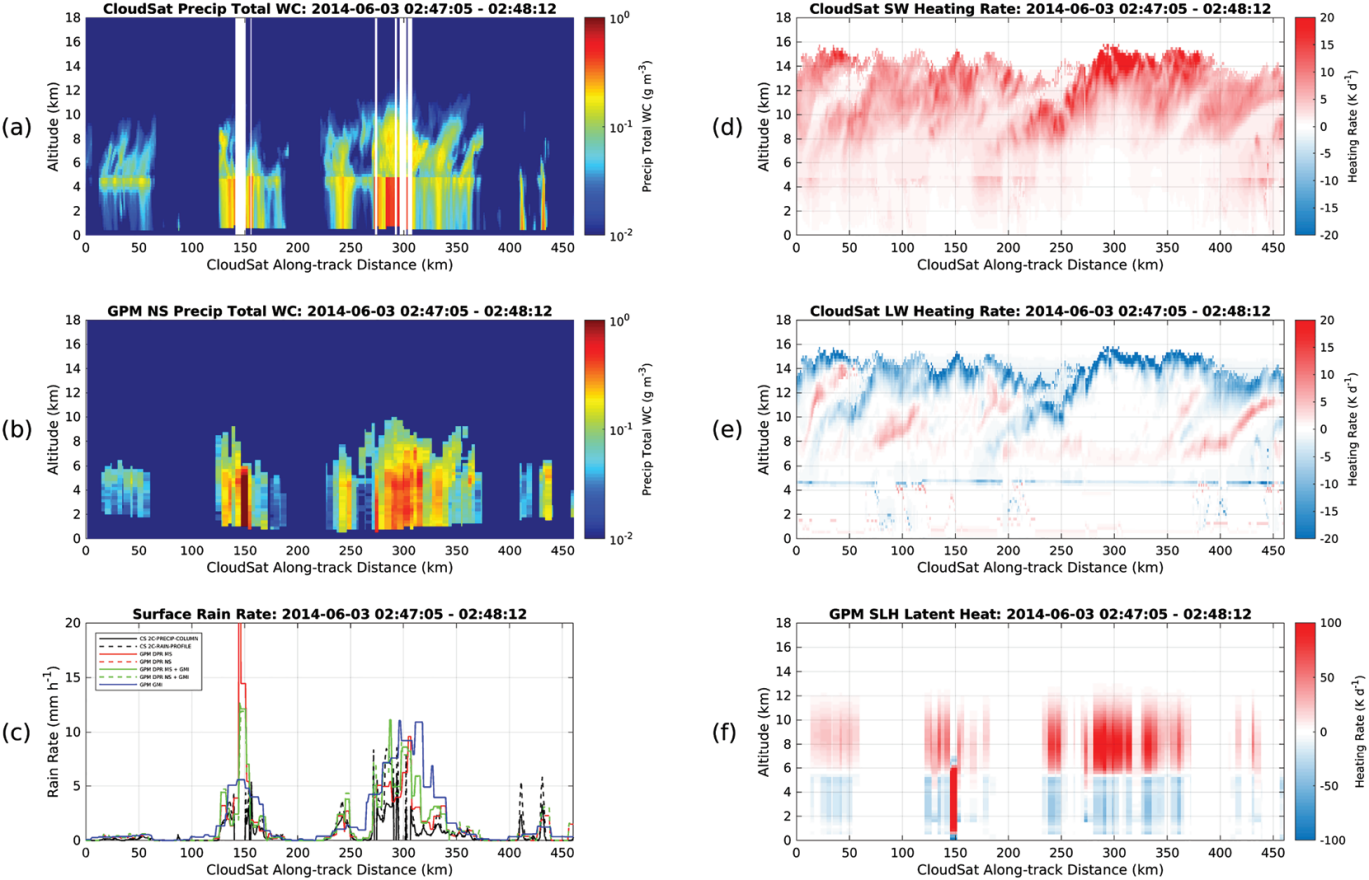
Collocated CloudSat and GPM water content and heating rate data. (a) Merged ice water content and precipitation liquid water content from the 2C‐RAIN‐PROFILE CloudSat product; (b) GPM normal‐scan liquid water content; (c) surface rain rates obtained using CloudSat 2C‐PRECIP COLUMN (black solid line), CloudSat 2C‐RAIN‐PROFILE (black dashed line), GPM DPR matched scan from 2A.GPM.DPR (red solid line), GPM DPR normal scan from 2A.GPM.DPR (red dashed line), GPM DPR matched scan and radiometer from 2B.GPM.DPRGMI.CORRA (green solid line), GPM DPR normal scan and radiometer from 2B.GPM.DPRGMI.CORRA (green dashed line), and GPM radiometer only from 2A.GPM.GMI.GPROF (blue solid line). (d) CloudSat shortwave heating rate from the 2B‐FLXHR data product; (e) CloudSat longwave heating rate from the 2B‐FLXHR data product; and (f) GPM latent heat from the 2A.GPM.DPR.GPM‐SLH data product. The white vertical lines in panel (a) correspond to nonconvergence of the 2C‐RAIN‐PROFILE algorithm.

1. *Complementarity*. Different frequencies are generally tailored to different targets, with higher (lower) frequencies more suitable to observe clouds (precipitation) because of their better sensitivity (reduced attenuation). For instance the CloudSat W band radar is capable of detecting the vast majority the high clouds above 10 km and the regions of light precipitation (e.g., between 60 and 100 km) (black rectangles in the top panel) which are completely undetected by the Ku and Ka band radars. On the other hand, the Ku and Ka can profile precipitation in the most intense part of the precipitation (black rectangles in the center and bottom panels) where the W band is fully attenuated (e.g., at about 150 km). Cloud radar observations allow to characterize vertical profiles of the microphysical properties (e.g., ice and liquid water content illustrated in Figure 22a), from which the cloud radiative forcing can be inferred (e.g., cooling and heating rates in Figure 22d). Precipitation radar observations, on the other hand, are exploited to produce precipitation products (Figure 22b) and from these derive latent heat profiles mainly from prime principles and model‐based climatologies (Figure 22f). All these quantities are of key importance with respect to the energy budget (Figure 1).
2. *Synergy*. In the regions where all radar signals remain above their detection thresholds they can be exploited synergistically for retrieving cloud microphysical properties, thus reducing the uncertainties associated with single‐frequency retrievals. For instance the large‐ice and low‐medium precipitation regions are detected both by the GPM and the CloudSat radars (black circles in all panels) with the measured signals being the result of the complex interplay between non‐Rayleigh, attenuation and higher order effects (see discussion in Appendix A1). These differential signals in backscattering and attenuation provide additional information (beyond the sixth moment of the particle size distribution that is provided by the radar reflectivity under the Rayleigh assumption) that can be used to constrain the retrieval of key hydrometeor population properties (e.g., water content, characteristic size). It is clear that single sensor products are still burdened by large uncertainties; this can be recognized by comparing the liquid water contents shown in Figures 22a and 22b, which have been derived by the CloudSat and GPM algorithm, respectively. The level of uncertainties is reflected in the estimates of the surface precipitation (Figure 22c) with variability between different sensors frequently exceeding a factor of 2. Some of this uncertainty could be reduced in principle by deploying multiple spaceborne radars of different frequencies.

Unfortunately, within the current observing systems measurements such as those shown in Figure 21 are very rare, only being available during “coincident overpasses” of the CloudSat and GPM‐core satellites (typically four or five per day).

The technology review (section 4.1) suggests that the capabilities required to advance the use of multiwavelength radar measurements from space are readily available. The advancements can be done along two main directions:

1. Process‐oriented studies with nadir or narrow swath modes (of the order of 15–20 km in order to capture the 3‐D organization at the convective‐scale) with emphasis on the quality of the observations (i.e., sensitivity, resolution, and Doppler accuracy);
2. Precipitation/cloud “mapping” observations with wide swath scanning modes focused at reducing revisiting time and sampling errors and at capturing diurnal cycle variability (Tan et al., 2019; Watters & Battaglia, 2019). Intermediate swaths (30–40 km) are also recommended when using the radar observations to calibrate radiometer algorithms. Such observations will address the need to extend the observational record beginning with TRMM PR and continuing with GPM‐DPR.

Hereafter we focus on the first approach and discuss possible ways to tackle the scientific gaps highlighted in section 3 by exploiting the new technological advances. When considering multifrequency nadir modes, performances like those listed in Table 4 are now in reach. As a result, significant enhancement of detection levels with respect to current capabilities are expected especially at the lower frequencies (Ka and below).

**Table 4:** Summary of Approximate Specifications for Upcoming Spaceborne Radars (Partially Adapted From Leinonen et al., 2015)

Frequency (GHz)	Single pulse MDT (dBZ)	Footprint size (km)
9.6	19	5
13.6	13	4
35.5	0	1.5
94.4	−21	0.6
220	−21	0.3

Note. A flying altitude of 450 km and an antenna size of the order of 2.5 m have been assumed. Shaded in gray frequencies which are not currently employed in spaceborne radars. Note that the radar sensitivity after integration scales roughly with the square root of the number of pulses (e.g., when integrating over 500 m with a PRF of 6 kHz it improves by roughly 13 dB).

**Figure 23:**
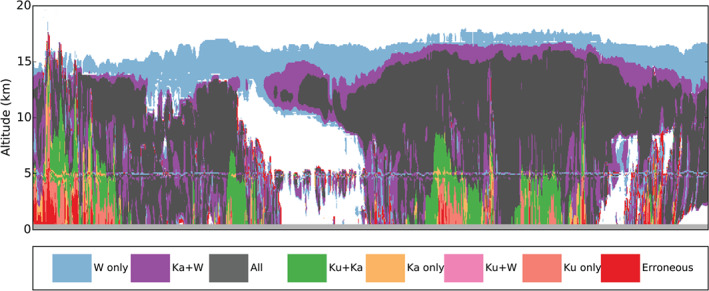
An overview of the radar band and multifrequency “availability” in the case study of a vertical cross section through a tropical maritime organized convection for a Ku‐Ka‐W system with specifics as in Table 4. Extracted from Leinonen et al. (2015) ©European Geosciences Union. Used with permission.

**Figure 24:**
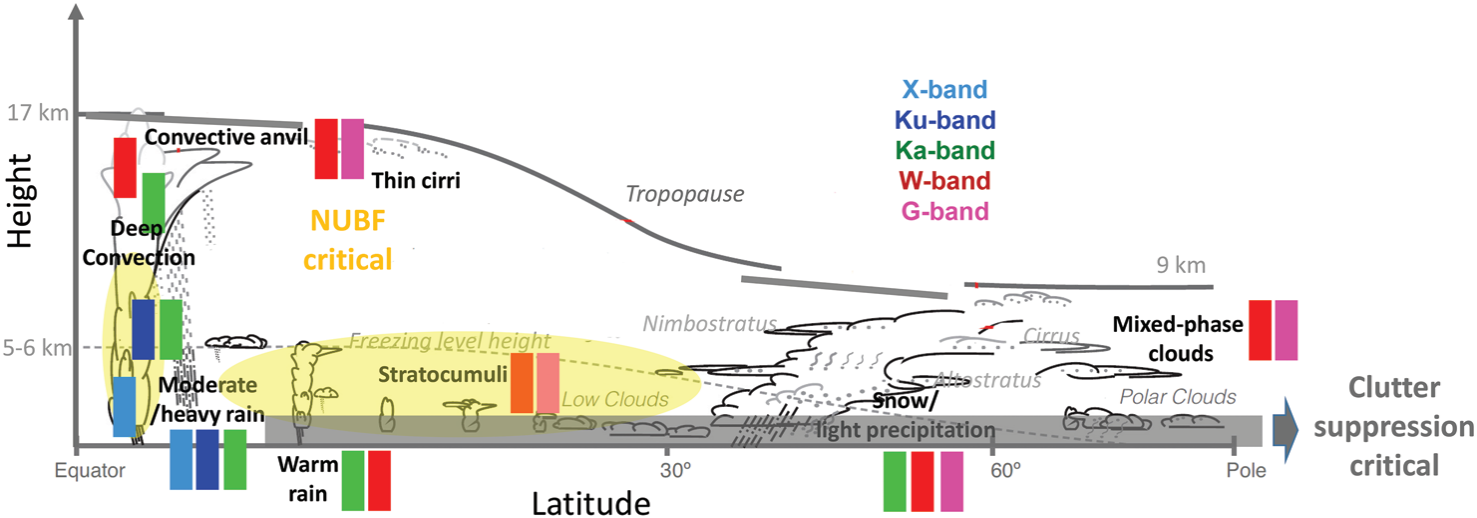
Cloud systems and science themes where a future multifrequency radar system can provide novel information. Areas shaded in yellow (gray) correspond to regions where nonuniform‐beam‐filling (clutter suppression) corrections maybe critical.

The benefit of such a sensitivity improvement for global cloud and precipitation observations has been neatly demonstrated for a Ku‐Ka‐W system by Leinonen et al. (2015), who examined which parts of Earth's cloud systems can be mapped by triple‐frequency measurements. Figure 23 shows that, for the case of a vertical cross section through tropical maritime organized convection, the signal from all three bands is typically available (dark gray color), except for the thin cirrus clouds and the heavy precipitating cell. Only few regions are mapped by single frequencies or are labeled as “erroneous” (i.e., the system has not adequate capabilities). When considering global statistics, triple‐ or dual‐frequency retrievals are available in roughly two thirds of the bins for the configuration listed in Table 4. Regions that are mapped by a single frequency (roughly one third) correspond to low reflectivities that can be detected only by the W band radar and are populated by small particles scattering in the Rayleigh regime; thus, no synergistic approach is in any case applicable to them.

The advances in technology have also paved the way toward the use of new frequencies (in the X and G band, shaded in gray in Table 4); with current technologies dual‐frequency studies in the region above 10, 0, −15, −30 dBZ for the X/Ku, Ku/Ka, Ka/W, and W/G, respectively, are realistic. Figure 24 summarizes the areas where different set of frequencies can be effective with specific focus at the scientific gaps identified in section 3. The lower frequencies are useful for larger particles and deep systems: Ku band is increasingly useful going toward the tropics while X band can characterize extreme events involving heavy rainfall and/or hail. Besides the GPM science goals the Ku‐Ka pair with enhanced sensitivity, as proposed for the GPM follow‐up, can better target light precipitation and snow. The Ka‐W pair is great for sensitivity, has huge potential in thick ice clouds and in light and moderate rain. Pairs including a radar in the G band represent a new frontier particularly for ice and mixed phase layer microphysical characterization, for water vapor profiling in ice and boundary layer clouds but also for the characterization of warm clouds.

### 4.3 Ice/Snow Microphysics and Ice Processes

**Figure 25:**
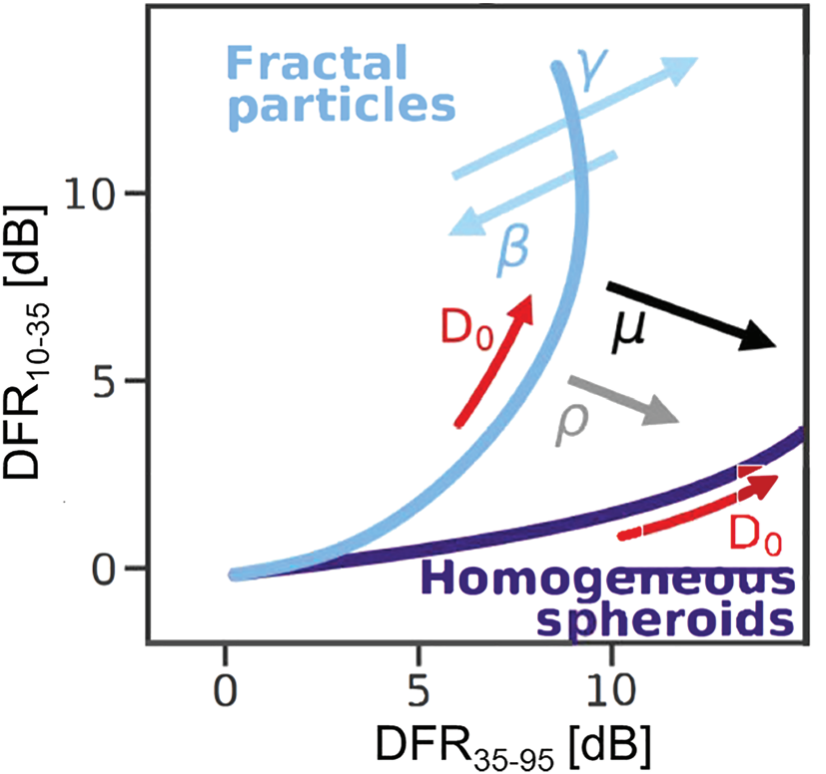
Impact of characteristic size, ice density, PSD shape parameter, and internal structure parameters (*γ* and *β*) to the triple‐frequency radar signature in the *D* *F* *R*_*Ka*−*W*_‐*D* *F* *R*_*X*−*Ka*_ plane for a population of ice particles. Extracted from Mason et al. (2019) ©European Geosciences Union. Used with permission.

Multifrequency radar observations are especially valuable in ice/snow cloud conditions since ice crystals are complex with large variability in microphysical properties (e.g., density, size, shape), making the interpretation of single‐frequency radar observations extremely challenging. Dual‐frequency first (Matrosov, 1998; Hogan et al., 2000; Liao et al., 2016) and later triple‐frequency (13, 35, and 94 GHz) methods have been proposed to characterize ice microphysics (Battaglia et al., 2020; Kneifel et al., 2011; Kneifel et al., 2015; Kulie et al., 2014; Leinonen et al., 2012; Leinonen & Moisseev, 2015; Stein et al., 2015). These methods rely on the fact that, in the “non‐Rayleigh” regime, the measured reflectivity changes (typically decreases) relative to the Rayleigh reference, because the incident wave backscattered from different parts of the ice particle interferes (typically in a destructive way) with itself. Kneifel et al. (2015) proposed to use the measurements of two DFR pairs (e.g., Ka‐W and X‐Ka) to deduce information about the characteristic size and the bulk density of the ice population, with low‐density unrimed aggregates or dendrites and high‐density spheroidal particles distinctively clustering in separate regions of the *DFR*_*Ka*−*W*_−*DFR*_*X*−*Ka*_ plane (see Figure 25). By performing a sensitivity study Mason et al. (2019) further discovered that, in addition to characteristic size and density of the PSD, the shape of the particle distribution (i.e., the *μ* parameter in the Γ‐function parametrization) and, secondarily, the internal structure of aggregate crystals are additional factors affecting the triple‐frequency radar signature of a population of ice particles. Therefore, even triple‐frequency observations may not be enough to disentangle all the complexity of ice particles: Specific ice particles can be unambiguously identified only in a minority of cases, for example, for fluffy aggregates when the characteristic hook signature is observed (Chase et al., 2018b; Kneifel et al., 2015) but most of the observations lay in the region where multiple solutions are possible (Chase et al., 2018b; Tridon et al., 2019). Nevertheless, Grecu et al. (2018) found that triple‐frequency retrievals are less sensitive to the “a priori” distribution of PSD parameters than dual‐frequency retrievals. Additional information from in situ measurements or Doppler information are found to further improve the solution of the problem (Mason et al., 2018; Mason et al., 2019). The retrieval is also further complicated by the uncertainty in correcting for ice and liquid water extinction at W band. The addition of a higher frequency in the G band can be certainly beneficial to better disentangle some of the residual microphysical uncertainties (Battaglia et al., 2014).

### 4.4 Liquid Precipitation

**Figure A3:**
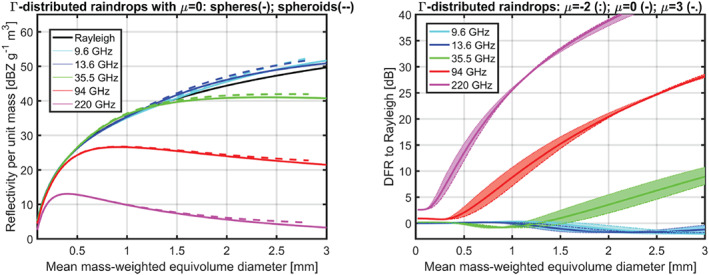
(left) Radar reflectivity coefficients for different frequencies (as indicated in the legend) for an exponential population of raindrops with different mean mass‐weighted equivolume diameters for a liquid water content of 1 g/m^3^. Scattering properties are computed for spheres by Mie theory (continuous lines) and for spheroids at vertical incidence by T‐matrix (dashed lines). (right) Same as in the left panel but for *D* *F* *R* with respect to Rayleigh. The shaded regions indicate values of *μ* between −2 (upper boundary) and 3 (lower boundary).

**Figure A6:**
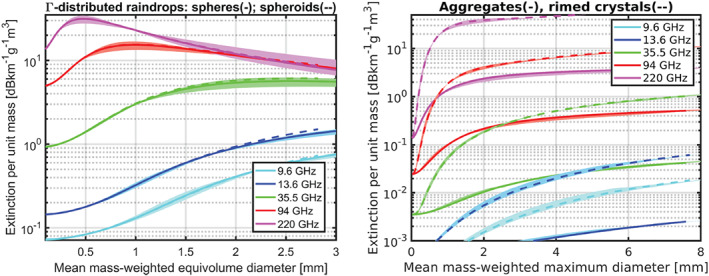
(left) Extinction for different frequencies (as indicated in the legend) for an exponential population of raindrops with different mean mass‐weighted equivolume diameters. Scattering properties are computed for spheres by Mie theory (continuous lines) and for spheroids at vertical incidence by T‐matrix (dashed lines). The shading corresponds to a variability in the shape parameter *μ* between −2 and 3. (right) Same as in the left panel but for ice crystals. The shading corresponds to a variability in the shape parameter *μ* between −1 and 3 with continuous lines for aggregates and dashed lines for heavily rimed crystals.

Similarly, in order to overcome the large uncertainties characteristic of single‐frequency radar microphysical retrievals, multiwavelength approach have been proposed for liquid precipitation as well. While the Ku‐Ka GPM pair has been extensively studied (Liao et al., 2014; Liao & Meneghini, 2019; Rose & Chandrasekar, 2006; Seto et al., 2013; Seto & Iguchi, 2015) other pairs and triplets of frequencies have received less attention. In general because of the dissimilar sensitivities that are achievable and of the distinct level of attenuations, different frequencies will be more useful for specific rain microphysics regimes. As already discussed in section 2.3 and demonstrated in Figure 21, millimeter‐wavelength radars, while designed for measuring properties of nonprecipitating clouds, are essential assets in light precipitation detection and characterization both from the ground (Giangrande et al., 2010; Matrosov, 2005; Matrosov et al., 2006) and from space (Berg et al., 2010; Haynes et al., 2009) thanks to their enhanced sensitivity (see Table 4) and non‐Rayleigh effects (see Figure A3 or Figure 6 in Battaglia, Mroz, Lang, et al., 2016). The range of millimeter radars is limited due to high levels of attenuation (see left panel of Figure A6) which can drive the radar‐received power from ranges beyond heavy rain layers into the noise level (e.g., Figure 21a, around 300 km). Vice versa attenuation may be used as a source of information for estimating rainfall rate (Matrosov et al., 2008).

**Figure 26:**
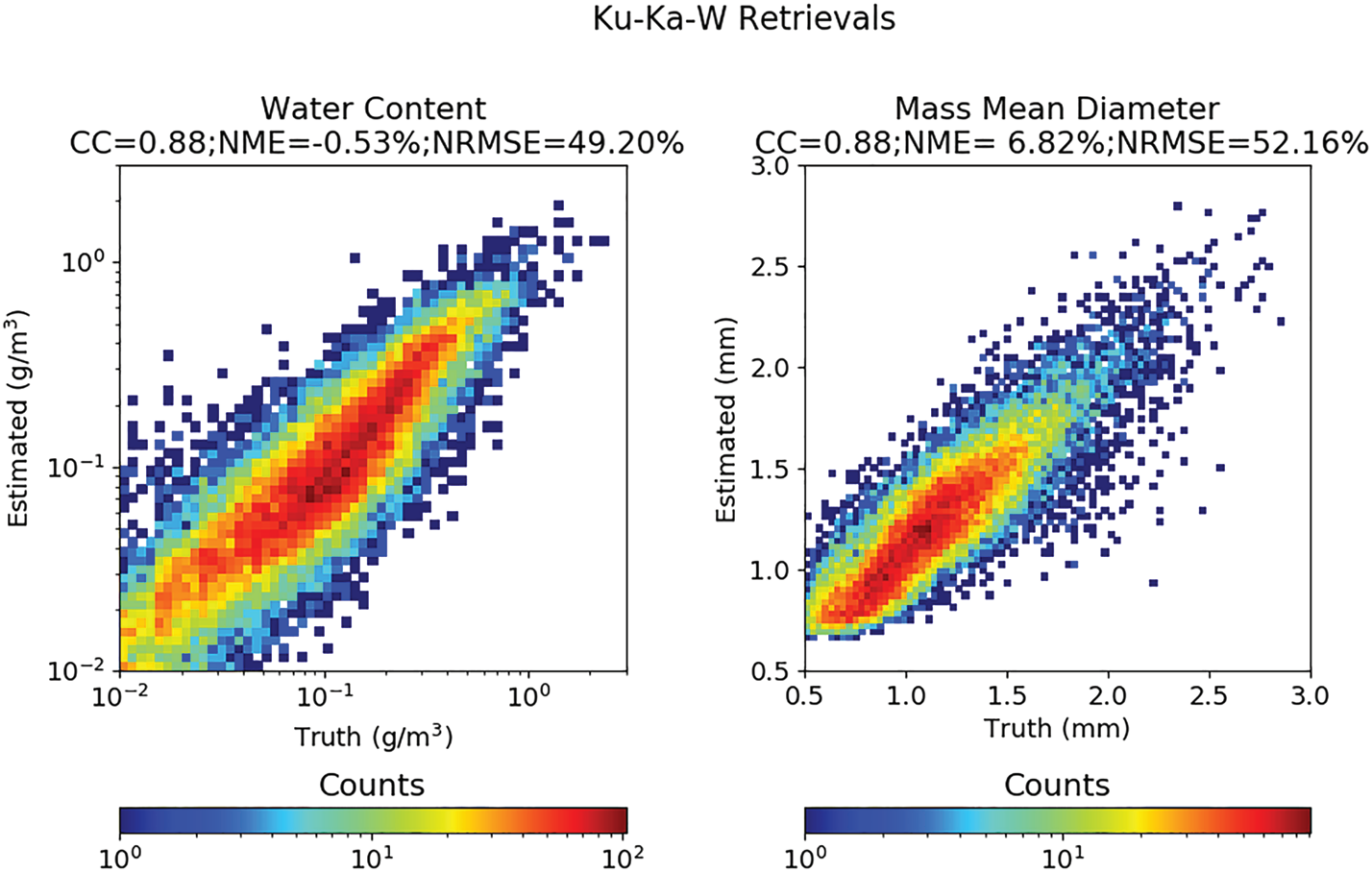
Estimated (left) water content and (right) mass mean diameter derived from computed Ku, Ka, and W band reflectivity observations versus the respective true water content and mean diameter used in the observations synthesis. The DSD data used in the computation of the “a priori” reflectivities are derived from DSD observation collected at NASA Wallops Facility, while those used in evaluations were collected during OLYMPEX.

To derive additional and more quantitative insight into the benefit of multiple frequency spaceborne radar observations in quantifying of properties of liquid, a simulation experiment similar to that of Grecu et al. (2018) was conducted. Specifically, DSDs collected at the NASA Wallops Flight Facility (Tokay et al., 2016) and during the OLYMPEX field experiment (Houze et al., 2017) were used to simulate radar reflectivity observations at Ku, Ka, and W band. A simple Lagrangian model, similar to that of (Adhikari & Nakamura, 2003), was used to convert the time‐dependent DSD observations into vertical precipitation structures. The model is based on the assumption that rain drops observed on the ground at a given time *t* are the same as the rain drops at height *v*_*T*_ × *dt* at time *t* − *dt*, where *v*_*T*_ is an average fall velocity and *dt* is a time interval on the order of minutes. Assuming *v*_*T*_ ≈ 8 m s^−1^, sets of six consecutive 1 min DSDs were thus converted into columns of precipitation with 500.0 m vertical resolution. A larger number of consecutive observations would have resulted in unrealistic vertical variability as DSDs tend to decorrelate fast, while a smaller number would have led to less conclusive evaluations. The vertical precipitation structures and their associated DSDs were then converted to triple‐frequency reflectivity observations. The attenuation at the top of the rain layer was considered zero, while the attenuation due to rain drops within the structures was accounted for. The nonparametric estimation methodology of Grecu et al. (2018) was used to investigate accuracy of retrieved rain water contents and mass weight mean diameter for various combination of radar wavelengths. The methodology relies on “a priori” database and an efficient search methodology that finds observations in the “a priori” database similar to the actual observations. The “a priori” database is derived from DSD observations collected at the NASA Wallops Flight Facility (Tokay et al., 2016), while the evaluation observations are derived from DSDs collected during OLYMPEX (Houze et al., 2017). The statistical scores considered in the evaluation are the same as in Grecu et al. (2018): the coefficient of correlation (CC), the normalized root‐mean‐square error (NRMSE), and the normalized mean error (NME). Results are summarized in Table 5. As apparent in the table, triple‐frequency, Ku‐Ka‐W, retrievals are superior to dual‐frequency retrievals. This is similar to the findings of Grecu et al. (2018) for the ice phase. Although the Ku‐W combination appears to perform almost as well as the triple‐frequency combinations, it should be noted, that the DSDs used in the study are predominantly characteristic of stratiform precipitation. In real applications, in deeper convective precipitation structures, the attenuation may be so severe that only the Ku band observations remain above the noise level. In such situations, as explained in section 4.2, a triple‐frequency Ku‐Ka‐W system is expected to perform significantly better than a Ku‐W system. Shown in Figure 26 is the joint distribution of retrieved and true water contents and mass mean diameters, which are consistent with the scores in Table 5.

**Table 5:** Statistical Scores Quantifying the Agreement Between Estimates and the True Values

		WC			*D*_*m*_
Observations	CC	NRMSE (%)	NME (%)	CC	NRMSE (%)	NME (%)
Ku‐Ka	0.78	64.31	−15.08	0.87	61.72	11.34
Ku‐W	0.85	54.46	−3.78	0.86	57.64	6.96
Ka‐W	0.82	68.08	12.08	0.78	72.48	10.06
Ku‐Ka‐W	0.88	49.22	−0.53	0.88	52.16	6.82

### 4.5 Boundary Layer Cloud and Precipitation Systems

A number of radar architecture modifications could improve our ability to detect boundary layer systems, especially modifications to the following:

1. The pulse length, which controls the range resolution and the vertical extent of the surface echo; in current systems, radar range resolution is such that shallow clouds only partially fill the radar beam and thus tend to go undetected and the vertical extent of the surface echo masks cloud bases and precipitation falling below 1.2 km;
2. The radar minimum detectable signal, which is currently too high to detect tenuous nonprecipitating clouds;
3. The radar horizontal footprint, which is currently too coarse to resolve feature inside individual cumulus.

Because thin and weakly reflective clouds are generally the main contributors to cloud cover in low‐level cloud regimes, Lamer et al. (2020) determined that improving radar minimum detectable signal is more important than reducing the vertical extent of the surface clutter for the purpose of improving cloud cover estimates. In their study, using a combination of high‐resolution high‐sensitivity ground‐based radar observations and instrument forward‐simulators, they determined that the 7 dB more sensitive EarthCARE‐CPR (Illingworth et al., 2015) should detect significantly (19.7%) more cloudy columns than the CloudSat‐CPR, only missing <9.0% of the simulated cloudy columns. Besides its enhanced sensitivity, the EarthCARE‐CPR is also planned to have a ground footprint half the size of the CloudSat‐CPR; comparison of forward simulations of the cloud system that formed at the Atmospheric Radiation Measurement (ARM) Eastern North Atlantic (ENA) facility on 7 December 2015 show how the EarthCARE‐CPR's architecture will improve our ability to detect tenuous, thin and broken boundary layer clouds as well as precipitation from space (compare Figures 15c and 15d).

On the other hand, statistical comparison of real CloudSat‐CPR and ground‐based observations suggest that the CloudSat‐CPR is able to capture the general vertical distribution of hydrometeor (i.e., hydrometeor fraction profile) above 750 m (Lamer et al., 2020). They attribute this success to (i) the CloudSat‐CPR's ability to detect the deeper more reflective clouds forming in these regimes, which seemingly have the largest impact on vertical profiles of cloud fraction but also to (ii) the fact that the CloudSat‐CPR does not experience important partial beam filling (and associated cloud stretching) issues because its sensitivity and pulse length are proportional to the intensity and size of the cloud systems it can characterize. Lamer et al. (2020) estimate that unlike the CloudSat‐CPR, the EarthCARE‐CPR will tend to overestimate hydrometeor fraction by up to 7% at all heights between 500 m and 3.0 km because the lower minimum detectable signal it will achieve (−35 dBZ) will also be collected using a long pulse (∼⃒1 km). Hydrometeor fraction overestimation will be caused both by the EarthCARE‐CPR being sensitive to cloud further away from their actual distance as well as to cloud layers shallower than its pulse length which will appear stretch (e.g., the cloud system found at 575 km along track in Figure 15c). This highlights the importance of adjusting radar minimum detectable signal and pulse length simultaneously in a way proportional to the relationship between cloud thickness and cloud mean reflectivity. Although synergy with lidars may help mitigate artificial cloud top stretching in the EarthCARE‐CPR's observations, alternative solution for multilayered systems and cloud base biases will need to be developed. But beyond biasing cloud morphology estimates, partial beam filling issues may affect the ability to accurately measure their true reflectivity (Burns et al., 2016). Such radar reflectivity biases would affect water mass retrievals performed using radar reflectivity measurement and future efforts should aim at quantifying this effect and should look into alternative retrieval techniques and/or radar configurations that could tackle this issue (Battaglia et al., 2020).

Besides its enhanced sensitivity, the EarthCARE‐CPR will also operate a pulse of which range weighting function is asymmetrical around its peak (personal communication with mission's engineering team): The leading edge of the pulse has a rapid cut off at a factor of 1 time the pulse length while the trailing edge has a longer taper extending off to 3 times the pulse length. Forward simulation results suggest that the new pulse shape design would successfully allow for the detection of a larger fraction around 750 m above ground that being said, even with all its radar architecture modifications, the EarthCARE‐CPR would not be able to detect rain to the point of being able to determine if it will reach the surface or evaporate (compare Figures 15a and 15d). Overcoming the remaining detection limitation would require further reducing the vertical extent of the surface echo. While this could be achieved by reducing the radar pulse length, this would also incur a loss in radar sensitivity. Comparing forward simulations of the EarthCARE (Figure 15c), ACCP_250_ (Figure 15e), and ACCP_100_ (Figure 15f) Cloud Precipitation Radars allows us to weight the gain and penalty incurred from shortening the range resolution from 500, to 250, and to 100 m. Reducing the radar pulse length, while overall reducing the fraction of detected hydrometeors, improves the characterization (both in terms of echo top and echo base location) of those echoes which are detected.

Given this trade‐off, it appears that the optimum choice of radar architecture should be dictated by the research objective. When it comes to nonprecipitating cloud cover, the more sensitive EarthCARE‐CPR configuration best matches observations collected by the ground‐based KAZR; when it comes to the characterization of cloud top height, the precision achieved by the 250 m range resolution ACCP‐CPR configuration produces echo top height statistics most comparable to the ground‐based KAZR; when it comes to documenting virga base height and surface precipitation the 100 m range resolution ACCP‐CPR configuration produces virga base height statistics most comparable to the ground‐based KAZR. The alternative of deploying spaceborne radars capable of operating with interlaced operation modes is also worth considering (Kollias et al., 2007). For example, a radar capable of generating both a highly sensitive long‐pulse mode and a less sensitive but clutter limiting short‐pulse mode would likely provide a more comprehensive characterization of the boundary layer by detecting both low‐reflectivity clouds and low‐altitude rain.

### 4.6 Convective Transport

**Figure 27:**
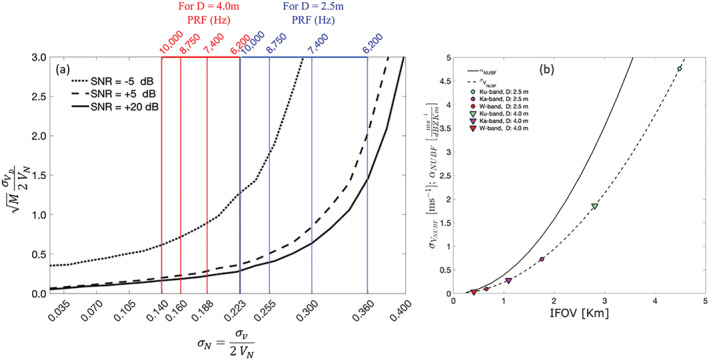
(a) Normalized Doppler velocity uncertainty ( $\sigma_{V_{D}}$) as a function of normalized spectral width (*σ*_*N*_) for three different SNR values. The blue lines indicate the *σ*_*N*_ for a spaceborne radar with an antenna diameter of 2.5 m and for different PRF values, and the red lines indicate the *σ*_*N*_ for a spaceborne radar with an antenna diameter of 4.0 m and for different PRF values. The corresponding Doppler velocity uncertainties ( $\sigma_{V_{D}}$) are shown in Table 6. (b) The NUBF Doppler velocity bias correction coefficient *α*_*NUBF*_ (solid line) and the Doppler velocity uncertainty $\sigma_{V_{NUBF}}$ introduced to the estimated Doppler velocity due to uncertainty in the *α*_*NUBF*_ value.

The importance of knowing the vertical transports of water vapor and condensate by atmospheric moist convection cannot be overstated (Stephens et al., 2020). As discussed in section 3.5, the estimation of vertical air motion in deep convection from the only planned spaceborne radar mission with Doppler capability (EarthCARE) will be challenging. The speed of the spaceborne platform (V_*sat*_ ≃ 7,600 m s^−1^ at an altitude of 400 km) is the main source of uncertainty in the Doppler velocity measurements (Kollias et al., 2014). The platform motion generates apparent Doppler velocity motion to the targets within the radar resolution volume, thus increasing the variance of the Doppler velocity, as expressed by the Doppler spectrum width, *σ*_*v*_. The impact of higher Doppler spectral width on the quality of the Doppler velocity measurements is shown in Figure 27a. The parameters that are critical for the performance (uncertainty) of the Doppler velocity estimator are the normalized spectral width (*σ*_*N*_ = *σ*_*v*_/(2*V*_*N*_)), the signal‐to‐noise ratio (SNR) and the number *M* of processed samples per estimate (Figure 27a). Most ground‐based radars operate at small normalized spectrum width values (*σ*_*N*_ < 0.1), and thus, the uncertainty in their Doppler velocity estimates is negligible. In spaceborne radars, *σ*_*N*_ is independent of the radar wavelength and is given by

(1) $$\sigma_{N}=C\frac{V_{sat}}{PRF\;D}$$

where *C* is a nondimensional constant, *V*_*sat*_ is the platform motion in m s^−1^, PRF is the Pulse Repetition Frequency in Hz and *D* is the antenna size (m). The *σ*_*N*_ values for two different antenna sizes (2.5 and 4.0 m) and for different PRF values are shown in Figure 27a and the corresponding Doppler velocity uncertainties are reported in Table 6.

**Table 6:** Standard Deviation of the Doppler Velocity (m s^−1^) Measured by a Spaceborne Doppler Radar for Different Combinations of PRF, Antenna Size, and Radar Frequency (Rows 3–6)

		Antenna diameter: 2.5 m			Antenna diameter: 4.0 m		
		Ku band	Ka band	W band	Ku band	Ka band	W band
Pulse	6,200	3.65	2.28	1.39	0.72	0.45	0.27
Repetition	7,400	1.45	0.91	0.55	0.57	0.35	0.21
Frequency	8,750	0.89	0.55	0.33	0.50	0.31	0.19
(PRF, Hz)	10,000	0.71	0.44	0.27	0.47	0.29	0.18
IFOV (km)		4.5	1.75	0.7	2.8	1.1	0.4
NUBF error (ms^−1^)		4.76	0.72	0.1	1.85	0.28	0.04

*Note*. Row 7 indicates the integrated field of view (IFOV) in the troposphere for each radar system, and Row 8 indicates an estimate of the error introduced by the NUBF velocity bias correction.

The EarthCARE CPR operates with a PRF between 6,200 and 7,400 Hz and has a 2.5 m antenna. There is significant reduction in the uncertainty (from 1.39 to 0.55 m s^−1^) between these two PRFs. It is important to state that these PRF values are the maximum allowed for a spacecraft operating at an altitude of 400 km if we want to avoid having more than one pulse in the tropospheric data window (20 and 12 km heights, respectively). Using higher PRF values is technologically possible but will require more than one operating PRF to remove the second trip echoes. The use of higher PRF values and of multi‐operating modes will drive up the power requirements for such a radar system. The use of a larger antenna (4.0 m) indicates that we can achieve low Doppler velocity uncertainties without the need to increase the PRF above 7,400 Hz.

Cloud and precipitation inhomogeneity within the radar beam sampling volume (NUBF) is another source of biases on mean Doppler velocity estimates from spaceborne Doppler radars. The NUBF effect can introduce biases that can reach several meters per second, especially at the cloud and precipitation field edges, and in areas with large variability in reflectivity and velocity (Sy et al., 2014; Tanelli et al., 2002). The NUBF Doppler velocity bias expressed in m s^−1^/(dB km^−1^) corresponding to a linear, along track radar reflectivity gradient is given by (Sy et al., 2014)

(2) $$\alpha_{NUBF}=\frac{V_{sat}}{H_{sat}}\;\frac{ln(10)}{40\sqrt{2\pi ln(2)}}\;{IFOV}^{2}$$

where *H*_*sat*_ is the altitude of the satellite and *IFOV* is the Instantaneous Field of View of the spaceborne radar in the troposphere. Values of *IFOV* for different radar frequencies and antenna sizes are shown in Table 6 and Figure 27b. Multiplied by the along‐track radar reflectivity gradient, the slope *α*_*NUBF*_ provides an estimate of the NUBF Doppler velocity bias. In real data, we can compensate for this bias using a theoretical estimate of the slope *α*_*NUBF*_ and an estimate of the along‐track radar reflectivity gradient. Using a 20% uncertainty in the estimation of the slope *α*_*NUBF*_ and a typical 3 dB km^−1^ along‐track radar reflectivity gradient (Kollias et al., 2014), an estimate of the uncertainty introduced by the NUBF Doppler velocity bias correction is estimated (dotted line, Figure 27b). For the same antenna size and satellite altitude, the IFOV linearly depends on the radar wavelength; thus, lower frequency radars will have considerably higher uncertainty in their NUBF Doppler velocity correction (last row, Table 6). Thus, although the use of lower radar frequencies allows deeper penetration in deep convection, this comes at the expense of larger IFOVs and considerably higher NUBF‐introduced uncertainty in Doppler velocity estimates (∝*IFOV* ^2^).

The aforementioned analysis is relevant to future radar concepts that plan to use a single antenna Doppler radar system to acquire Doppler velocity measurements from space. At Ka band and lower frequencies that offer more penetration to convective clouds, this translates to the need to have a fairly large antenna (4–5 m diameter) and a very high PRF that will violate the range‐Doppler dilemma resulting to second trip echoes within the troposphere. Large solid surfaces have the advantage that can be a shared resource for more than one frequency (i.e., WiSCR and 3CPR radar concepts discussed in section 4.1); however, their deployment is challenging especially in multisensor missions that share the same satellite bus. Nevertheless, the potential to deploy a large deployable antenna at low radar frequencies (Ku or X band) using a SmallSat for Doppler measurements is within our technological capabilities in the near future.

On the other hand, the need to increase the PRF while maintaining our ability to probe the entire troposphere without range artifacts can be addressed using either polarization diversity (PD;  Battaglia et al., 2013; Battaglia & Kollias, 2015; Illingworth et al., 2018) or copolar interleave pulse transmitting schemes (Kollias et al., 2007). PD was introduced first by Doviak and Sirmans (1973) on ground‐based weather radar to overcome multitrip echoes and was later used by Pazmany et al. (1999) on a ground‐based 94 GHz Doppler radar to study tornadoes. It requires a more complicated transmit scheme and a dual‐channel receiver for receiving both the copolar and cross‐polar radar signals. The implementation of the PD scheme in a spaceborne Doppler radar includes several technological challenges and more complex transmit and scheme and receiver signal processing but can improve the Doppler estimates for a single antenna spaceborne radar system.

The copolar interleave pulse transmitting scheme uses short duration (burst) trains of pulses with different pulse length and PRF schemes. A low PRF train of pulses can be used to map the hydrometeor locations in the troposphere while 2 or more high PRF trains of pulses can be used to provide higher quality Doppler but with second trip echoes in particular vertical layers in the atmosphere. In the end, data from all the different trains of pulses are combined to generate an artifact‐free mosaic of hydrometeor location and Doppler velocities in the troposphere. In addition to differences in their PRF, the different bursts of pulses can have different pulse lengths, thus addressing some of the shortcomings in detecting hydrometeors close to Earth's surface.

Despite their added complexity, these techniques (PD and interleave pulse schemes) are technologically possible in future missions. One additional drawback that needs to be considered is the power requirement for such a high duty cycle transmitting schemes. Combining these schemes with a low‐power system that uses chirp could help to alleviate some of the concerns regarding the power requirements.

**Figure 28:**
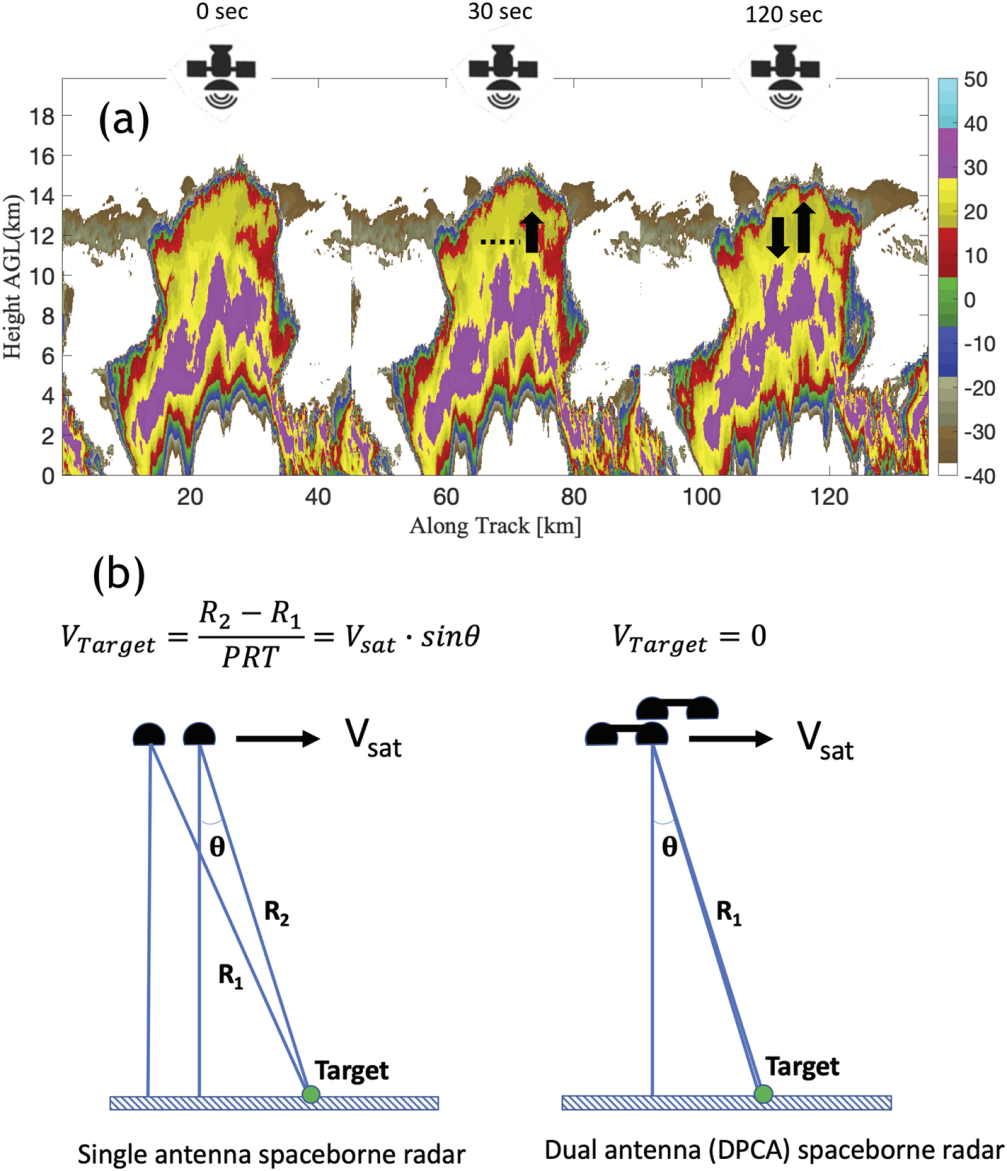
(a) A temporal sequence of three time snapshots 0, 30, and 120 s apart of the same deep convective cloud described in Figure 17, as seen by a CubeSat Ka band radar with a 1.6 m antenna. The arrows indicate the relative motion of the radar reflectivity echoes as depicted using echo correlation techniques. (b) One way of implementing the DPCA technique, see text for details. In the second panel the radar is displaced vertically for clarity.

Over the last few years, two different technological approaches to address the need for spaceborne Doppler velocity measurements in deep convection have been developed and are briefly mentioned here (Figure 28).

1. The first concept is based on the recent successful spaceborne demonstration of a miniaturized, low‐cost CubeSat precipitation radar (RainCube;  Peral et al., 2019). The low cost of such a radar, together with the availability of small satellite platforms to carry it, now make it feasible to consider employing a more distributed approach to observe important atmospheric processes that relate to precipitation (Stephens et al., 2020). A mission concept, referred to as D(dynamical)‐train, comprising a train of three satellites 30, 90, and 120 s apart (Figure 28a) is described by Stephens, van den Heever, et al. (2020). In a nutshell, the time‐spaced radar echoes of the same deep convective cloud can be used to estimate the vertical transport of radar reflectivity and subsequently, the vertical transport in deep convective clouds. As depicted in Figure 28a, the same deep convective cloud is sampled at three different time instances by three non‐Dopplerized Ka band radars. Using time‐correlation techniques we can infer the vertical transport (ascend, descend or no movement) of the different radar reflectivity structures observed by the mini radar constellation at different time intervals. These observations allow then to retrieve the air and condensed water fluxes (see Figure 16). Because deep convection is ubiquitous in the tropics, the focus of a convection‐focused mission should be in such region and should also properly sample the diurnal cycle.
2. The second concept is the Displaced Phased Antenna Concept (DPCA), where the idea is to make the antenna appear stationary even though the platform is moving (Figure 28b; Durden et al., 2007). The estimation of Doppler velocity requires a pair of measurements from two pulses spaced by Pulse Repetition Time (PRT, sec). In a single antenna spaceborne radar configuration, the distance between a stationary target (motionless green circle in Figure 28b) and the radar changes from the first pulse (*R*_1_) to the second pulse (*R*_2_) due to the platform motion (*V*_*sat*_). Thus, the target has an apparent Doppler velocity *V*_*target*_≠0 that broadens (decorrelates) the radar signal and leads to increased uncertainty. In a possible DPCA dual‐antenna spaceborne radar configuration, the first pulse is transmitted by the forward antenna when at distance *R*_1_ from the target and the second pulse is transmitted from the trailing antenna when at the same location where the first antenna transmitted the first pulse. In this case, the second pulse travels the same distance as the first pulse (which requires matching the PRT with the product of the platform velocity and the antenna baseline); thus, there is no apparent Doppler velocity. This is one of the ways of implementing the DPCA technique and can lead to high quality Doppler measurements from a fast moving platform because it practically eliminates the platform motion effect and, as a result, the uncertainties discussed in Figure 27. In addition, the DPCA technique can work at low PRF values, thereby minimizing the power requirement of the radar concept. The only drawback of the DPCA technique is that the presence of two antennas limits the size of the antenna that can be used and thus results in larger IFOV values (about 2.5 km for a 1.6 m antenna at Ka band).

### 4.7 Water Vapor Measurements in Cloudy Areas: Differential Absorption Radar

Recently, a new water vapor measurement technique has emerged led by efforts at JPL to fill the important observational gap of measuring humidity from space in the presence of clouds and precipitation. This technique, called differential absorption radar (DAR), is inspired by the well‐established differential absorption lidar (DIAL) measurement method (Browell et al., 1979, 1998), and combines the precise ranging from radar with the strong frequency dependence of gas attenuation near an H_2_O rotational absorption line to perform active sounding of humidity profiles and column water vapor. Because the radar measurement signal‐to‐noise increases with increasing cloud content, the DAR method is highly complementary to DIAL, which cannot perform the differential absorption measurement inside of clouds due to the confounding effects of multiple scattering. From a TCWV standpoint, DAR is an attractive potential spaceborne method because it uses the strong returns from the surface to perform the column water vapor measurement over all surface types, in cloudy and clear skies, and at all times of day.

As with multifrequency radars for cloud microphysics studies, the fundamental DAR measurement is a dual‐frequency ratio, but with closely spaced frequency channels *f*_1_ and *f*_2_ = *f*_1_ + *Δf*, where *Δf*/*f*_1_ ≪ 1. In this case, it is the gas attenuation effect that is exploited to retrieve humidity profiles inside of clouds and precipitation, while the signals arising from differences in effective reflectivity factors and hydrometeor attenuation effects are ideally small due to the close frequency spacing. Under the assumption of negligible frequency dependence of cloud backscatter and attenuation, one can show that

(3) $$\frac{\partial }{\partial r}\;DFR=2\left[\kappa_{v}(f_{2},r)-\kappa_{v}(f_{1},r)\right]\;\rho_{v}(r),$$

where *κ*_*v*_ is the mass extinction cross section for water vapor in units of dB·km^−1^ g^−1^ m^3^ and *ρ*_*v*_ is the absolute humidity profile along the radar beam. Equation 3 provides for a simple retrieval scheme where measured reflectivities at two frequencies for two ranges *r*_1_ and *r*_2_ are used to estimate the average humidity between the two ranges. For TCWV retrievals using a nadir‐pointing DAR, the known radar calibration factors for the two frequencies *f*_1_ and *f*_2_ play the role of the near‐range measurement at *r*_1_. For the case of in‐cloud humidity profiling, it is important to note that because the DAR measurement involves ratios of radar power measurements at different frequencies *and* ranges, no system calibration is necessary.

While ground‐based and airborne DIAL systems have provided high‐quality humidity profiling measurements in cloud‐free atmospheres for many decades, it is only recently that such a technique could be applied to cloud radars, because the first available H_2_O absorption line for performing the analog measurement is at 183 GHz. Though DAR systems with tones within the lower‐frequency weak H_2_O line at 22 GHz have been proposed (Meneghini et al., 2005) to study the link between water vapor and rainfall, a radar operating near this line would have a weak sensitivity to water vapor and be insensitive to clouds. Thus, in order to develop a water vapor DAR instrument, it is also necessary to develop the first modern G band cloud radar. It is interesting to note that there were G band cloud measurements attempted in the 1980s using vacuum tube electronic sources (Mead et al., 1989; Nemarich et al., n.d.Wallace, 1988), but these had limited sensitivity and narrow tuning bandwidths, precluding them from being used for DAR. Therefore, in addition to humidity profiling capabilities, the G band scattering measurements from a water vapor DAR can be combined with scattering at Ka and W band for novel studies of ice and snow microphysical properties (Battaglia et al., 2014).

Early instrument simulation studies suggested the utility of a spaceborne G band DAR for providing valuable humidity measurements in cloudy areas. These included investigations of DAR in the context of shallow convection and planetary boundary layer humidity (Lebsock et al., 2015) as well as frontal, stratiform clouds and precipitation using CloudSat‐generated microphysical products (Millán et al., 2016). These notional studies were followed by an instrument development effort by the JPL group, leveraging significant technological development at JPL in Schottky‐diode, frequency‐multiplication‐based sources in the millimeter, submillimeter, and terahertz frequency ranges (Siles et al., 2018), as well as frequency‐modulated, continuous‐wave (FMCW) radar advancements for low‐power, millimeter and submillimeter wave solid‐state transmitters (Cooper et al., 2011; Cooper et al., 2017). The JPL project, called the Vapor In‐cloud Profiling Radar (VIPR), demonstrated the DAR measurement for the first time while operating between 183 and 193 GHz (Cooper et al., 2018). In addition to being the first DAR, VIPR is the first solid‐state cloud radar above W band, showing promise for future systems throughout the G band for multifrequency studies.

**Figure 29:**
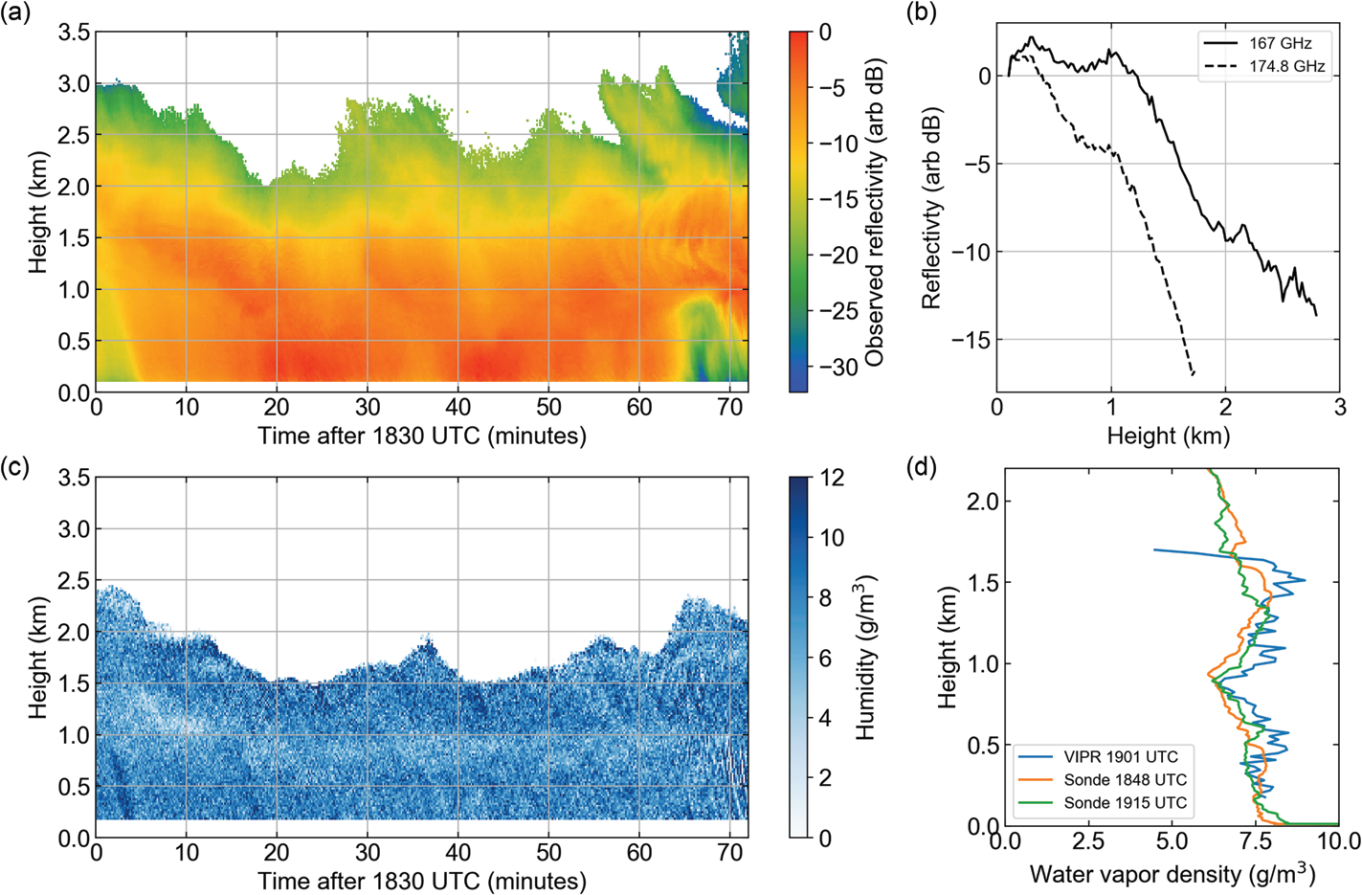
Radiosonde‐validated measurements from the VIPR water vapor DAR at Scripps Institute of Oceanography. (a) Uncalibrated, G band (167 GHz) radar reflectivity. (b) Comparison of reflectivity profiles at 167 and 174.8 GHz with 5 s temporal resolution, where each trace is normalized to its corresponding value at 100 m. The excess absorption from water vapor at 174.8 GHz is clearly apparent with increasing range. (c) Retrieved humidity map for the same scene measured in (a). (d) Comparison of two radiosonde humidity profiles with that from DAR with 5 min of averaging.

Due to severe transmission restrictions from international regulations in this band, the VIPR system was modified to operate in the less‐restrictive 167–174.8 GHz band, making it most sensitive to the moist planetaryboundary layer because of the decreased value of *κ*_*v*_(*f*_2_)−*κ*_*v*_(*f*_1_). Subsequently, the first in‐cloud humidity profile retrievals with uncertainty estimation were presented (Roy et al., 2018). More recently, DAR humidity profiles and column water vapor retrieved from VIPR reflectivity measurements were validated using coincident radiosonde measurements from the Department of Energy's Atmospheric Radiation Measurement Southern Great Plains (ARM‐SGP) site (Roy et al., 2020). This work employed an improved retrieval algorithm based on a regularized least squares framework and demonstrated the accuracy of the water vapor DAR method from the surface up to 10 km in height, with a typical in‐cloud humidity root‐mean‐square error (RMSE) of 0.5 g/m^3^ and column water vapor RMSE of 1.2 mm. An example of ground‐based DAR humidity measurements from VIPR are shown in Figure 29 using data from a separate validation experiment performed at the Scripp's Institute of Oceanography. The measurement scene consisted of low clouds and weak drizzle. Figure 29a displays the uncalibrated radar reflectivity at 167 GHz, and the lower panel the water vapor field retrieved using the 167/174.8 GHz differential reflectivity and the algorithm described in Roy et al. (2018). During this measurement window, two radiosondes were launched approximately 30 min apart, and revealed a temporally static vertical humidity profile. The comparison of in situ humidity with a 5 min average of DAR‐retrieved humidity values is shown in Figure 29d. As an application to multifrequency radar retrievals, one could imagine using the water vapor field in Figure 29c to correct the G band reflectivity measurements for gas attenuation before combing with other radar channels in a microphysical retrieval.

Following the initial success of the VIPR instrument demonstration, a recent study investigated using DAR for humidity sounding in ice clouds to provide microphysical process information (Battaglia & Kollias, 2019), like that associated with the saturation phase diagram in Figure 19. This work pointed out the importance of adding frequency channels over a larger transmission bandwidth in order to mitigate the biases resulting from frequency dependence of the hydrometeor scattering terms in the dual‐frequency ratio, which can be well approximated by linear functions of frequency over this band. However, a challenge in this and other respects for all future G band DAR systems will be the restrictions that limit the availability of transmit frequency locations. For instance, in order to be sensitive to the low absolute humidities inside of ice clouds, a G band DAR must operate near the peak of the 183 GHz line, which is a strongly protected band in terms of radar transmission due to the abundance of spaceborne passive sensors that occupy this region. An alternative solution to the problem of DAR humidity sounding in regions of low absolute humidity is to access the submillimeter‐wave absorption lines at 325 and 380 GHz, which are high enough in frequency that current regulations do not restrict radar transmission.

While the ground‐based results realized thus far highlight the unparalleled capabilities of DAR to remotely sense in‐cloud humidity, there is much work to be done before realizing a spaceborne DAR. The VIPR system began airborne testing in the fall of 2019, constituting an important milestone of demonstrating the DAR method from a nadir‐viewing platform, as well as using the surface reflection to measure total column water vapor. An important next step for airborne DAR in the context of the recent Decadal Survey and PBL measurement technology incubation is synergistic deployment alongside other humidity sounding platforms, especially clear‐air humidity profilers like NASA Langley's High Altitude Lidar Observatory (HALO) water vapor DIAL (Nehrir et al., 2017). Though the existing G band instrument technology is sufficient for sensitive cloud detection throughout the troposphere from a high‐altitude aircraft, significant technology development is required in order to realize a viable spaceborne sensor, specifically in the realm of high‐output‐power solid‐state transmitters. Potential avenues of investigation include massive power combining of numerous low‐power (order 100 mW) G band sources, which could be either GaAs‐based frequency multipliers or monolithic microwave integrated circuit (MMIC) power amplifiers. This power combining could feasibly be realized in a waveguide‐based fashion or with many individual feeds using a phased‐array technique.

### 4.8 Conclusions and Final Recommendations

Spaceborne radars offer unique information on the Earth's hydrological cycle, the vertical distribution of clouds and precipitation and the 3‐D structure of storms. Considering the ongoing discussions about the future of spaceborne radar measurements across several space agencies, the remaining measurement gaps from spaceborne radars have been identified in this review. Briefly, these measurements gaps are (i) accurate quantification of precipitation (particularly extreme precipitation in the tropics and light/solid precipitation over the midlatitude and high‐latitude regions) and of precipitation microphysics, (ii) detection of shallow clouds, (iii) Doppler in convective clouds, and (iv) in‐cloud water vapor sounding measurements. Mapping the diurnal convective clouds cycle is also a considerable gap. This is primarily a sampling issue that can be addressed only with the use of low‐cost constellation of CubeSats and SmallSats at different orbital planes. Thus, it is beyond the scope of this review that is primarily concern with improvements in the sensor level that can address the aforementioned gaps.

Progress in radar technologies facilitate novel research avenues thanks to improved radar performances (enhanced sensitivities, finer vertical and horizontal resolutions, better Doppler accuracies), unprecedented capabilities (e.g., electronic scanning at W band, systems with frequencies in the G band) and most cost effective solutions that can revolutionize the field of space‐based observations via the use of constellations of Cube/Small satellites. Here we suggest some critical directions that need to be expanded in the upcoming years.

#### 4.8.1 Monitoring and Operational Applications

Spaceborne radars represent the backbone of the global observing system for monitoring clouds and precipitation and their evolution in a changing climate. In this respect their role should become even more pivotal particularly in relation to three aspects.

1. *Unique mapping capabilities*. Because of their cloud‐penetrating range‐resolved profiling capabilities radars remain the only instruments capable of providing the vertical distributions of clouds and precipitation. The scientific community strives for a continuous long‐time records of such measurements. (GCOS Steering Committee, 2017) has drawn attention to the lack of a millimeter‐wave cloud profiling radar after EarthCARE and recommended that space agencies prepare a follow‐on mission to provide data continuity after EarthCARE. The same applies to a TRMM/GPM‐like follow‐up. It is highly desirable that, in a near future, cloud and precipitation radars may become an integral component of the operational Global Observing System, thus providing long‐term monitoring of clouds and precipitation and their change in a warming climate.
2. *Novel observations for data assimilation*. Recent work has paved the ground for Weather Centers to assimilate radar observations of the cloud and precipitation vertical structure. Preliminary studies have shown that such observations help to improve the initial conditions of global weather forecasts by providing consistent thermodynamic and dynamic cloud profiles; this leads to a reduction in the forecast errors, particularly for temperature, wind and precipitation (Janiskova & Fielding, 2018) and for typhoon track position (Okamoto et al., 2016). Doppler conically scanning radar concepts, tailored at providing global measurements of in‐cloud winds for further improving the accuracy and effectiveness of severe weather forecasts, have been recently investigated in ESA studies as well (Battaglia et al., 2018; Illingworth et al., 2018).
3. *“Calibrators” for radiometer algorithms*. Radars are now increasingly used as reference for passive instruments. For instance the GPM‐DPR is used to develop the “a priori” precipitation radiation database used by the Goddard Profiling algorithm (Kummerow et al., 2015) for the whole constellation of GPM microwave radiometers; similarly the CloudSat CPR has been used to calibrate microwave radiometer algorithms for snow (Rysman et al., 2018), for warm rain (Eastman et al., 2019), for ice (Pfreundschuh et al., 2019), or to identify biases in liquid water paths between visible and microwave passive algorithms (Lebsock & Su, 2014). Ideally, radars should therefore fly in tandem with radiometers, with their swath fully covering the radiometer footprints, thus providing very high quality reference products apt for building training data sets to radiometer statistical algorithms. The more physically‐based radar observations combined with the improved sampling of radiometer constellations should lead to better essential climate variable climatologies.

#### 4.8.2 Process‐Oriented Studies

Spaceborne radar measurements are critical in order to improve our understanding of water cycle related processes and inform and validate the next generation of global cloud resolving models (Satoh et al., 2019). Several solutions have been proposed in this review to address some of the most relevant scientific gaps. It is, however, clear that in order to succeed, the next generation of spaceborne radars must make full exploitation of the recent technology advancements and significantly push sensitivity, multifrequency, Doppler accuracy, vertical and horizontal resolutions compared to the current state‐of‐the‐art. Because of the expected high cost involved in building and launching such systems, a concerted effort by the different Space Agencies (as already demonstrated within the TRMM/GPM and EarthCARE missions) is likely the best pathway forward, with radars of different types and other instruments germane to these process studies (lidars and multispectral imagers) flying in constellation formation (like successfully demonstrated by the A‐Train example Stephens et al., 2018b).

#### 4.8.3 Refinement of Multifrequency Radar Microphysics Retrievals

Because of their crucial role as “calibrators,” radar products must be thoroughly assessed via a full characterization of their uncertainties. Since multifrequency observations and retrievals are still in their infancy, progress is expected in several directions

**Figure 30:**
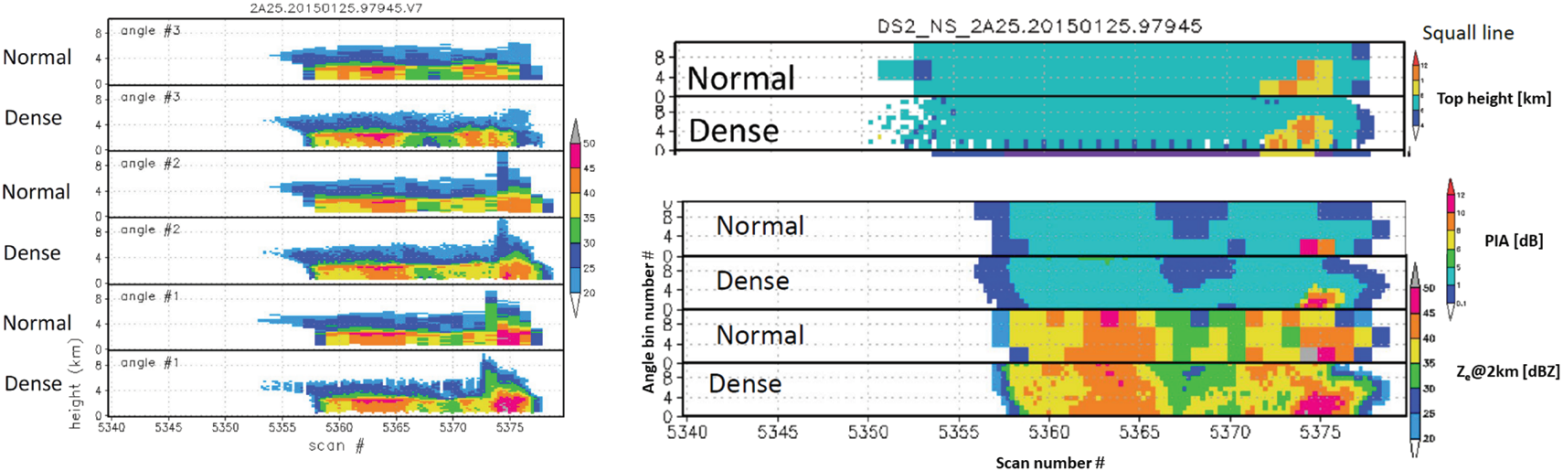
(left) Vertical reflectivity profiles for a squall line sampled at different angles for the normal and in oversampling mode during the TRMM end‐of‐mission experiment. (right) Improvement in the top height (compare top two panels), PIA (center panels), and reflectivity cut at 2 km height (bottom panels) by using oversampling. Courtesy of N. Takahashi.

**Figure 31:**
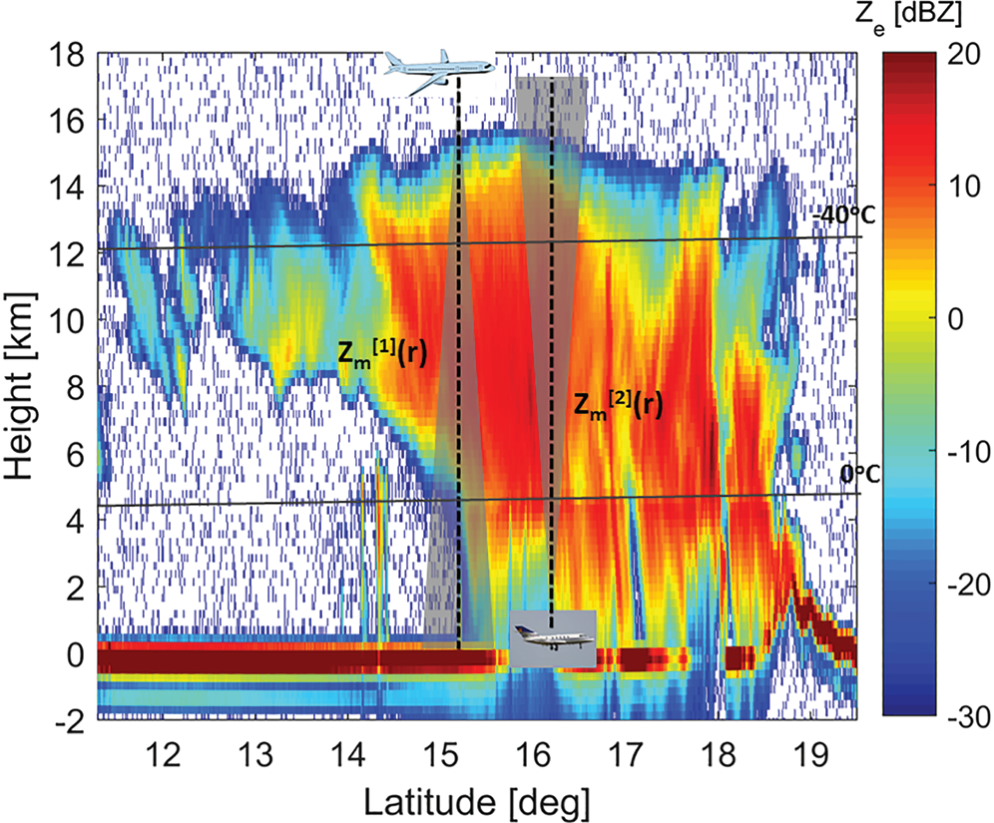
Proposed aircraft field campaign configuration to characterize extinction profiles. The rain scene is a CloudSat image of Tropical Storm Ernesto (26 August 2006).

1. Suborbital research programs and validation activities provide an unparalleled trove of information and remain paramount for assessing retrieval performances. There is a specific need of high quality dual‐ and triple‐wavelength radar observations (e.g., matched volume, high accuracy in alignment, high SNR) from airborne (e.g.,  Battaglia et al., 2016; Chase et al., 2018a; Heymsfield et al., 2018) and ground‐based (e.g.,  Kneifel et al., 2015; Stein et al., 2015) platforms coordinated with high‐quality in situ validation. In particular, for multifrequency studies, it is mandatory to focus the validation onto the “non‐Rayleigh” regions of the cloud (e.g., in rain or in presence of ice particles exceeding 1 mm, like typically found in the aggregation areas close to the melting level, or regions with heavily rimed crystals/graupel) with in situ instruments that are capable of sampling the whole spectrum of particles sizes (in ice up to several centimeters) and that can confidently report total mass of ice exceeding 0.5 g/m^3^. Such in situ measurements could also contribute in refining a‐priori assumptions (e.g., how ice *D*_*m*_ and *IWC* at a given temperature are related to reflectivities and how they covary), which, as recently highlighted by Protat et al. (2019a) for rain, may be regime/latitude dependent. In situ measurements below the freezing level remain essential to validate full‐column retrievals.
2. Scattering models are still sources of large uncertainties especially at frequencies above the Ka band. The availability of a limited number of ice models for different degrees of riming prevent continuous retrievals of ice properties. Future work should aim at the development and use of an ice model providing seamless description of ice properties and scattering cross sections as function of the degree of riming similarly to what was proposed by Leinonen et al. (2018) and Mason et al. (2018). Criteria for identifying predominant scattering regimes (ice density behavior) should be also driven by the spatial structure of multifrequency observables (including Doppler).
3. MS and NUBF effects can be better quantified and flagged if additional/more sophisticated measurements are planned. MS occurrence is difficult to quantify from *Z* alone. Linear depolarization ratios is a very good indicator of how much of the signal is due to multiply scattered radiation because MS strongly depolarize the signal, thus sharply increasing single‐scattering radar linear depolarization. This has been demonstrated both theoretically (Battaglia et al., 2008) and from airborne measurements (Battaglia et al., 2007). Incidentally linear depolarization is also useful for phase discrimination (Kumagai et al., 1993). This measurement therefore should be included in future systems targeting deep convection where MS is known to play an important role. For NUBF, oversampling the radar field of view is certainly beneficial to identify NUBF‐affected profiles as demonstrated by the use of the GPM‐DPR Ka high‐sensitivity scan mode (Mroz et al., 2018). As discussed in section 4.6along‐track oversampling will be critical for obtaining unbiased Doppler estimates. Moreover radar field‐of‐view oversampling can indeed provide higher resolution details via deconvolution. Some tests performed with the scanning TRMM PR have been promising (see Figure 30). This could be particularly crucial when antenna size must remain small due to budget/payload constraints for observations of very inhomogeneous scenes (e.g., patchy warm rain).
4. Testing the accuracy of attenuation corrections in retrieval algorithms is critical; this is particularly sensitive at W band and at frequencies above, where attenuation can be caused already by rimed crystals but can be relevant already at Ka in presence of melting hydrometeors, rain or graupel. For instance, Protat et al. (2019b) noted that the GPM‐DPR attenuation correction is based on attenuation‐reflectivity relationships for convective and stratiform precipitation which tend to systematically overestimate attenuation especially at midlatitude and high latitude when compared to statistical relationships based on in situ disdrometer data sets.
  Innovative approaches to measure extinction profiles must be adopted. For instance having two radars operated at the same frequency pointing at each other allows a direct measurement of the extinction profile as described by Nishikawa et al. (2016) for a ground‐based setup or as conceptually illustrated in Figure 31 for an airborne configuration. A proxy for the latter configuration can be realized by repeated aircraft passes through the same cloud at different altitudes as done in Protat, Rauniyar, et al. (2019). These measurements should be pursued particularly at W and G band.
5. Intercomparison studies between retrieval techniques developed by different research groups should be performed for golden cases (i.e., with in situ validation). This could help in better assessing the relevance of different a‐priori assumptions (e.g., the flux continuity across the melting layer), the validity of the scattering tables utilized in the retrieval, the uncertainties introduced by using different single scattering properties (see  Ekelund & Eriksson, 2019; Kuo et al., 2016) and the impact of the uncertainties in the calibration and cross calibrations of the radars. Also this will implicitly establish whether retrieval errors are properly estimated.
6. The effective benefits, potentials and limitations of triple‐ versus double‐ and single‐frequency retrievals has not yet been fully unfolded, for example, the improved accuracy of precipitation retrievals for triple‐frequency systems in different regimes (light/moderate/heavy rain, snow, …) must be properly quantified. Also the impact and added value of additional information like Doppler velocities and colocated brightness temperatures (like for CloudSat) and/or PIA must be better understood (e.g., Liao & Meneghini, 2019).

### 4.9 Closing Remarks

Current knowledge of clouds and precipitation has highly benefitted from past (TRMM) and present spaceborne based radar missions (GPM, CloudSat, and RainCube) but important gaps still remain for a thorough understanding of the water cycle and its evolution in a warming climate. Spaceborne radars are and will remain the pillars of the cloud and precipitation remote sensing observing system; however, apart from the long‐waited EarthCARE radar, the next generation of cloud and precipitation radar systems has not been defined yet and no such instrument/mission is currently in the funded pipeline of any of the National Space Agencies. The current preparatory studies for the NASA Aerosol, Cloud, Convection and Precipitation concept provide a unique opportunity for the whole cloud and precipitation science community to come together and contribute in identifying the key scientific questions, in defining their associated science requirements, in elaborating the technical and cost‐effective solutions to address them, and in seeking support by coordinating funding at the national and international level. With a grim future of climate emergency in sight, this review calls for renewed efforts in these directions.

## Figures and Tables

**Figure A1:**
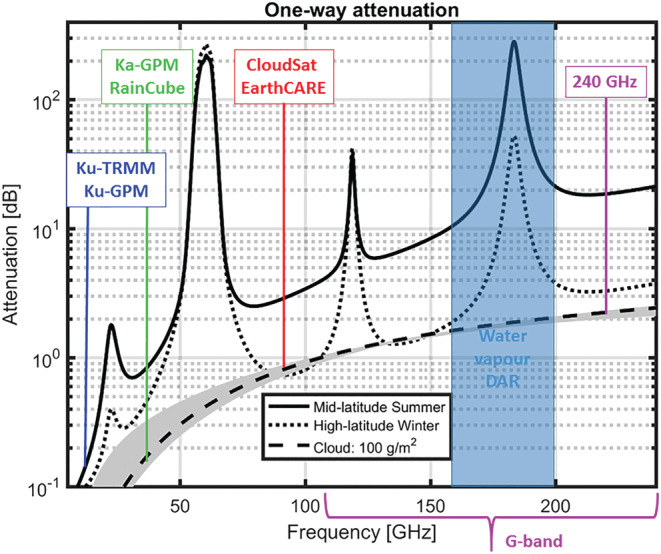
Gas attenuation in the range of frequencies between 5 and 250 GHz for two types of atmospheres (a very moist, midlatitude summer atmosphere and a very dry, high‐latitude winter atmosphere). Attenuation due to cloud water at 10°C for a total of 100 g/m^2^ is plotted in dashed line with the gray shading corresponding to the variability when moving temperature from −35°C to +30°C. Radar frequencies are generally selected in the window regions, that is, away from the water vapor and the oxygen absorption bands, apart for the differential absorption radar (DAR) with frequency located in the 183 GHz water vapor absorption band (blue shaded region). Allocation of current and planned spaceborne radar systems is shown as well.

**Figure A2:**
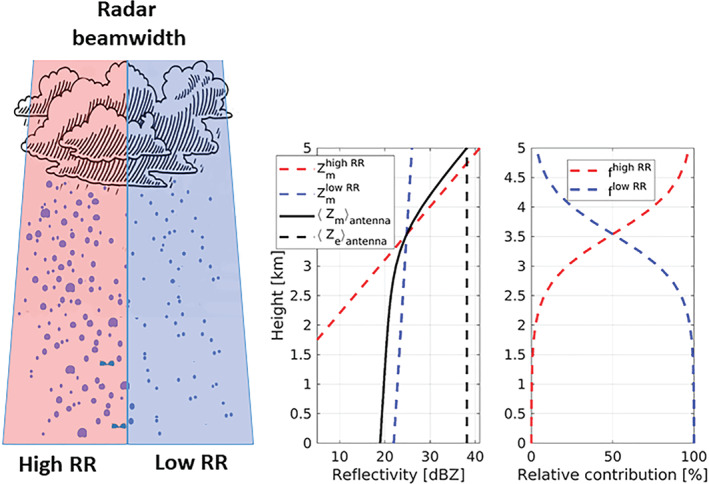
Effect of NUBF on the reflectivity profile for a radar beam half‐filled with a high rain rate and half‐filled with a low rain rate.

**Figure A4:**
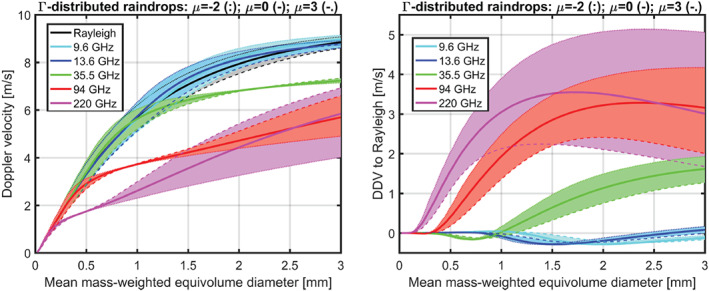
(left) Vertical Doppler velocities in the absence of vertical wind for different frequencies (as indicated in the legend) for an exponential population of raindrops with different mean mass‐weighted equivolume diameters. The shading corresponds to the variability in the shape parameter, *μ*, between −2 and 3. (right) Same as in the left panel but for the differential Doppler velocity (DDV) with respect to Rayleigh.

**Figure A5:**
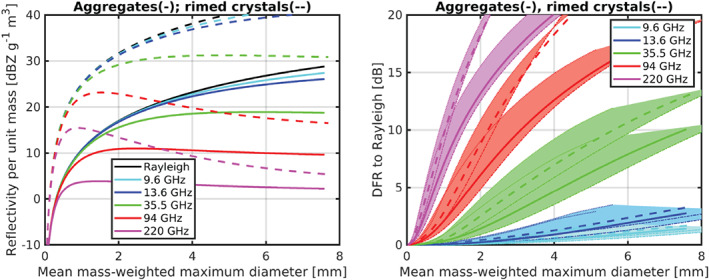
(left) Radar reflectivity coefficients for different frequencies (as indicated in the legend) for an exponential population of ice crystals with different mean mass‐weighted maximum diameters for an equivalent liquid water content of 1 g/m^3^. Scattering properties are computed at vertical incidence according to the self‐similar Rayleigh‐Gans approximation for the A1 model (continuous lines) and the A6 model (dashed lines) from Leinonen and Szyrmer (2015). The two models correspond to fluffy aggregates and heavily rimed crystals, respectively. (right) Same as in the left panel but for *D* *F* *R* with respect to the Rayleigh reference. The shading corresponds to a variability in the shape parameter *μ* between −2 and 3.

## Data Availability

Numerous satellite data sets are discussed in this review; they are all freely available (at https://pmm.nasa.gov/TRMM, https://pmm.nasa.gov/data-access/downloads/gpm, http://www.cloudsat.cira.colostate.edu/data-367products).

## Appendix A A Primer in Multiwavelength Doppler Radar Remote sensing

### A.1

Cloud and precipitation radars measure the range‐resolved power backscattered by clouds and precipitation present in the troposphere, *P*_*m*_(*r*). Via the radar equation for distributed targets (Meneghini & Kozu, 1990; Rauber & Nesbitt, 2018) (*C*_*radar*_ is a radar constant characteristic of each radar system) the measured (hereafter subscript *m* stands for measured) powered at range *r*:

(A1) $$P_{m}(r)=C_{radar}\frac{Z_{m}(r)}{r^{2}}$$

can be easily translated into range‐resolved profiles of reflectivity factors, *Z*_*m*_(*r*), corresponding to the radar volume located at range *r* and populated by distributed targets with a particle size distribution (PSD), *N*(*D*), expressed in particle per unit volume per unit length. The PSD expresses the concentration of particles as a function of the maximum particle size, *D*. From the PSD different microphysical quantities like

- The concentration
  $$N_{c}=\int_{0}^{+\infty }N(D)\;dD;$$
- The water content
  $$WC=\int_{0}^{+\infty }m(D)\;N(D)\;dD;$$
- The mass‐weighted size
  $$D_{m}=\frac{\int_{0}^{+\infty }D\;m(D)\;N(D)\;dD}{WC},$$

where *m*(*D*) is the mass of the particle, can be computed. Even though the complete forms of these integrals are often used in first approximation for analytical derivations, it is important to recognize that *N*(*D*) assumes a zero value beyond a maximum particle size which is subject of research by itself.

When neglecting second‐order effects (see section A0.1), measured and effective reflectivities are related by the following equation:

(A2) $$\underset{\text{dBZ}}{\underbrace{Z_{m}(f,r)}}=\underset{\text{dBZ}}{\underbrace{Z_{e}(f,r)}}-\underset{\text{Two‐way attenuation in dB}}{\underbrace{2\int_{0}^{r}k_{e}(f,r)\;dr}}$$

where we have explicitly introduced the dependence on the frequency (*f*) because all the scattering properties (backscattering, absorption and extinction cross sections) of cloud and precipitation hydrometeors strongly depend on the frequency of the transmitted radar wave, with attenuation and scattering becoming more and more important with increasing frequency (Kollias et al., 2007; Lhermitte, 1990; Ulaby et al., 1986). Hereafter we use the following definition for *Z*_*e*_ (Hogan et al., 2006):

(A3) $$Z_{e}(r)=\frac{\lambda^{4}}{0.93\;\pi^{5}}\;\underset{\eta (r)}{\underbrace{\int_{0}^{+\infty }\sigma_{b}(D)\;N(D)\;dD}}$$

where *η* is the radar reflectivity defined as the integral of the backscattering cross‐section *σ*_*b*_(*D*) over the particle size distribution, *λ* is the radar wavelength and 0.93 is the radar dielectric factor ( $|K{|}^{2}={\left|\frac{n_{w}^{2}-1}{n_{w}^{2}+2}\right|}^{2}$, with *n*_*w*_ the refractive index of water) of water at low microwave frequencies and 10°C. With this definition small ice particles have the same *Z*_*e*_ (because the refractive index of ice does not change significantly with frequency and temperature), whereas the reflectivities of small water droplets changes with frequency and temperature according to the corresponding change in *n*_*w*_. Because of its large dynamic range, reflectivity is typically reported in logarithmic units (1 dBZ = 10 $\log_{10}$[1 mm^6^/m^3^]). Note that in Equation A2, the extinction coefficient is expressed also in logarithmic units (e.g., in dB/m).

The measured reflectivity factor, *Z*_*m*_(*r*), in Equation A2 results from two processes:

1. The intrinsic backscattering of the targets present in the backscattering volume at range *r*, described by *Z*_*e*_(*r*), the effective reflectivity factor;
2. The attenuation of the radar electromagnetic wave caused by gases and hydrometeors contained within the path between the radar transmitter/receiver and atmospheric targets. Because the electromagnetic wave travels two‐way forward and backward between the radar transmitter/receiver and the target the total attenuation corresponds to twice the optical thickness obtained by integrating from the radar position to the range *r* the profile of the extinction coefficient, defined as
  (A4) $$k_{e}(r)=\int_{0}^{+\infty }\sigma_{e}(D)\;N(D)\;dD$$
  where *σ*_*e*_(*D*) is the extinction cross section. In SI units, *k*_*e*_ is expressed in *m* ^−1^; when logarithmic units are used then *k*_*e*_ (dBm^−1^) = 4.343 *k*_*e*_ (m^−1^). Most of the attenuation is caused by precipitation, though some results from the presence of atmospheric gases and nonprecipitating clouds (this amount in general grows in frequency, from negligible at X band to several dB at W band).

To minimize the impact of the atmospheric gasses on the measurements current cloud and precipitation radar systems have frequencies allocated in the atmospheric window regions, as depicted in Figure A1, far away from the absorption features. The only exception is the differential absorption radar (DAR) systems (see discussion in section 4.7) with tones within the 183.3 GHz (or potentially in the 325 and 380 GHz) water vapor absorption lines. Note that gas attenuation is already significant at W band (reaching 3 dB for midlatitude) and it is even stronger at the 240 GHz, where for moist environment it may exceed 20 dB (though it can be substantially reduced to less than 3 dB at high latitude in the colder months).

#### A.1.1 Second‐Order Effects: MS and NUBF

Equation A2 is strictly valid if multiple scattering (MS) effects are negligible. Battaglia et al. (2010) thoroughly reviewed MS effects in cloud and precipitation radar observations with clear evidences of MS on spaceborne radars provided in Battaglia et al. (2015) and Battaglia, Mroz, Tanelli, et al. (2016). MS is generally effective in optically thick scattering clouds where the instrument footprint becomes comparable with the scattering mean free path; it is generally very detrimental because it undermines the radar ranging capabilities by partially reducing the effect of attenuation. It also affects the Doppler both biasing the mean and widening the spectral width (Battaglia & Tanelli, 2011). When MS becomes extreme it can produce striking features like pulse stretching (Battaglia & Simmer, 2008; Hogan & Battaglia, 2008) or complete decorrelation of the Doppler signal but generally its effect can be very subtle and difficult to quantify. Because MS tends to highly depolarize radiation accurate quantification of MS effects could benefit from simultaneous measurements of linear depolarization ratios, which require receiving both in the cross and in the copolar channels (e.g.,  Heymsfield et al., 2010; Wolde et al., 2019).

Equation A2 is also only applicable when considering a pencil beam. With a finite antenna beamwidth the backscattered signal received by the instrument is the result of the convolution of the normalized antenna pattern, *G*_*n*_, with the measured radar reflectivity (hereafter indicated with the symbol ⟨⟩_*antenna*_):

(A5) $${\left\langle Z_{m}(f,r)\right\rangle }_{antenna}=10\;\log_{10}\left[\iint_{\Omega }G_{n}^{2}(\hat{\Omega })Z_{e}(f,r,\hat{\Omega })[{\text{mm}}^{6}{/m}^{3}]e^{-2\int_{0}^{r}k_{e}(f,r,\hat{\Omega })\;dr}d\Omega \right]$$

where the square accounts for the two‐way antenna gain. Thus, if scattering properties present a strong variability within the antenna beam (i.e., in presence of NUBF), the link between the antenna‐weighted measured reflectivity ⟨*Z*_*m*_(*f*,*r*)⟩_*antenna*_ and the antenna‐weighted effective reflectivity ⟨*Z*_*e*_(*f*,*r*)⟩_*antenna*_ can become quite complex. If attenuation is negligible or *k*_*e*_ does not depend on $\hat{\Omega }$ (e.g., with dominant gas absorption) then

(A6) $${\left\langle Z_{m}(f,r)\right\rangle }_{antenna}=10\;\log_{10}\left[\iint_{\Omega }G_{n}^{2}(\hat{\Omega })Z_{e}(f,r,\hat{\Omega })[{\text{mm}}^{6}{/m}^{3}]d\Omega e^{-2\int_{0}^{r}k_{e}(f,r)\;dr}\right]$$

(A7) $$={\left\langle Z_{e}(f,r)\right\rangle }_{antenna}-2\int_{0}^{r}k_{e}(f,r)[\text{dB}/m]\;dr$$

so that the attenuation decouples from the effective reflectivity: Equation A7 resembles Equation A2; thus, inversion algorithms which seek first to derive ⟨*Z*_*e*_(*f*,*r*)⟩_*antenna*_ via attenuation correction can work similarly to pencil beam configurations. Otherwise, the effect of the convolution is not trivial because attenuation and reflectivity are entangled. This is demonstrated with a simple example in Figure A2, where we consider a 35 GHz radar sampling a 5 km thick rain cell: Half of the footprint is filled by a constant rain rate of 20 mm/hr (high rain rate), the other half by 1.5 mm/hr (low rain rate). The high (low) rain rate has a reflectivity of 41 dBZ (26 dBZ) and a one‐way attenuation of 5.5 dB/km (0.4 dB/km); thus, ⟨*Z*_*e*_(*f*,*r*)⟩_*antenna*_ = 38.1 dB: Practically, there is a reduction of a factor of 2 (3 dB) because $Z_{e}^{highRR}\gg Z_{e}^{lowRR}$. The measured reflectivity is shown by the black continuous curve: While at the top the measured reflectivity is driven by the high rain rate component, as a result of differential attenuation within the beam, the contribution of the low rain rate becomes increasingly important when penetrating into the rain cell. For the specific example at 3.5 km the two contributions become equal, while below 2 km most of the radar signal (>97.5%) is produced by the low rain rate (right panel). As a result, the measured reflectivity value tends to bring less and less information about the presence of the large rain rate when approaching the surface; the slope of the measured reflectivity itself also does not carry such information because it tends to flatten out since it is more and more related to the attenuation of the low rain rate component when considering ranges closer and closer to the surface.

Even when reflectivity is constant at a given range within the antenna beamwidth (like when estimating the total attenuation with surface reference technique;  Nakamura, 1991; Meneghini et al., 2000; Meneghini et al., 2015) the interpretation of Equation A5 is complicated by the fact that the extinction enters in a nonlinear way with ${\left\langle e^{-2\int_{0}^{r}k_{e}(f,r)\;dr}\right\rangle }_{antenna}$ being generally different from $e^{-2{\left\langle \int_{0}^{r}k_{e}(f,r)\;dr\right\rangle }_{antenna}}$. This tends to cause biases in the estimates of antenna weighted attenuation (Durden & Tanelli, 2008; Meneghini et al., 2015), that could in principle provide an excellent constraint for attenuation correction algorithms.

#### A.1.2 Dual‐Frequency Ratios

When comparing measurements of reflectivities from two radars operating at different frequencies *f*_1_ and *f*_2_ (*f*_1_ < *f*_2_), it is possible to consider the dual‐frequency ratios (DFR), defined as their difference in logarithmic units (equivalent to their ratio in linear units):

(A8) $$DFR(f_{1},f_{2},r)[\text{dB}]\equiv Z_{m}(f_{1},r)-Z_{m}(f_{2},r)=\overset{\text{non‐Rayleigh effect}}{\overbrace{Z_{e}(f_{1},r)-Z_{e}(f_{2},r)}}+\overset{\text{attenuation effect}}{\overbrace{2\int_{0}^{r}\left[k_{e}(f_{2},r)-k_{e}(f_{1},r)\right]\;dr}}$$

where we have highlighted the two possible contributions to the DFR:

- “Non‐Rayleigh effects,” that is, differences in the effective reflectivity factors of the targets which occur when the hydrometeor sizes become comparable to the radar wavelength (Bohren & Huffman, 1983; Lhermitte, 1990);
- “Attenuation effects,” that is, differences in the attenuation properties along the propagation path, with higher attenuations produced at higher frequencies (Lhermitte, 1990).

Non‐Rayleigh effects are resulting from intensive properties of the PSD (e.g., characteristic size, spread of PSD) whereas attenuation effects can be used to infer extensive quantities (e.g., concentrations, rain rates, equivalent water contents). Again, in Equation A8 the second‐order effects (section A0.1), which can cause anomalous *DFR*s as shown in Battaglia, Tanelli, et al. (2014) and Mroz et al. (2018), are neglected.

#### A.1.3 Non‐Rayleigh Effects for Raindrops

For raindrops in the Rayleigh regime, the backscattering cross sections are proportional to the sixth power of the diameter and to the fourth power of the frequency (Bohren & Huffman, 1983). In non‐Rayleigh scattering regime, that is, when the raindrop size becomes comparable or it is larger than the wavelength, the backscattering power oscillates with consecutive maxima and minima with increasing size (e.g., Lhermitte, 1990). This oscillatory behavior is smoothed out when the integration over the particle size distribution is performed (here the mean mass‐weighted equivolume diameter, *D*_*m*_, is used as a characteristic size of the DSD). Results (left panel of Figure A3) are produced both for spheres via Mie theory (Bohren & Huffman, 1983) and for perfectly oriented spheroids via the “T‐matrix method” (a computational method of light scattering by nonspherical particles also known as extended boundary technique method) (Mishchenko, 2000) with axial ratios parameterized according to Andsager et al. (1999) at vertical illumination. Different features can be noticed:

1. X and Ku band reflectivities steadily increase with characteristic size. PSDs characterized by *D*_*m*_ below about 1.5 and 1 mm, respectively, scatter radiation according to Rayleigh approximation, whereas for larger sizes X and Ku band reflectivities exceed Rayleigh backscattering (super‐Rayleigh behavior) but never by more than 2 dB.
2. Spheroids tend to produce slightly larger reflectivities than equivolume spheres as expected from the larger geometrical cross sections orthogonal to the incident radiation at vertical incidence.
3. Ka reflectivities tend to plateau for *D*_*m*_ exceeding 1.5 mm, whereas W and G band reflectivities steadily decrease for *D*_*m*_ greater than 0.95 and 0.4 mm, respectively.
4. The values of the DFRs relative to Rayleigh reference in correspondence to small *D*_*m*_s (“cloud level”) are different from 0 because of the differences in the |*K*|^2^ factor of water and to our *Z*_*e*_ convention.
5. Very large “DFR to Rayleigh” are produced at W and G band; with a typical *D*_*m*_=1 mm almost 10 dB and more than 25 dB are expected at W and G band, respectively.
6. Ka, W, and G band “DFR to Rayleigh” exceed 1 dB with respect to the cloud level at approximately 1.5, 0.5, 0.2 mm, respectively, and are then steadily increasing above such sizes. These figures clearly define the size boundaries where significant DFRs are attainable.

Radar Doppler velocities are backscattering‐weighted averaged line‐of‐sight velocities *v*_*LOS*_ of particles within the radar backscattering volume:

(A9) $$v_{D}=\frac{\int_{0}^{+\infty }v_{LOS}(D)\sigma_{b}(D)N(D)\;dD}{\int_{0}^{+\infty }\sigma_{b}(D)\;N(D)\;dD}$$

and are derived by the phase change between the transmitted and the backscattered EM wave. Doppler velocities measured at vertical incidence are a very good indicator of PSD characteristic size because raindrop terminal velocity is a monotonically increasing function of its size (Atlas et al., 1973). Also, Doppler velocities are not affected by attenuation as the change of phase of the incident and backscattered radiation is independent of the backscatter ratio; this makes them a potentially excellent observable for the characterization of intensive properties. Doppler velocities are generally increasing with *D*_*m*_ (Figure A4) but such increase is significantly reduced when moving from the Rayleigh/X/Ku region into the Ka and even more into the W and G band frequency range because of the increasingly non‐Rayleigh behavior of the backscattering cross sections weighting the velocities of drops in Equation A9. The increase of *v*_*D*_ with *D*_*m*_ can therefore be used to derive *D*_*m*_ but only under the assumption of negligible vertical wind. Otherwise, the effect of vertical wind cancels out when considering differential Doppler velocities (see Matrosov, 2017, and references therein), that is, when subtracting Doppler velocities at two frequencies. For differential Doppler velocities computed with respect to the Rayleigh reference, it is possible to see that signals exceeding 1 m s^−1^ are expected at 35.5, 94, and 220 GHz for *D*_*m*_ larger than 1.9, 0.5, and 0.3 mm. Once again, the potential of high frequencies in pushing the sizing capabilities to small particles is obvious.

#### A.1.4 Non‐Rayleigh Effects for Ice Crystals

Because of the variety of ice habits and shapes, the computation of scattering properties of ice crystals is much more complex than for raindrops (Ekelund & Eriksson, 2019; Kuo et al., 2016; Kneifel et al., 2020, and references therein); while at small sizes backscattering cross sections are proportional to the square of the mass of the crystals (Hogan et al., 2006), when approaching large sizes the mass distribution within the particle along the direction of the impinging radiation plays a key role in affecting the particles scattering properties (e.g.,  Hogan & Westbrook, 2014).

As an example of backscattering calculations for exponentially distributed ice crystals, the results for unrimed and strongly rimed snow aggregates modeled according to Leinonen and Szyrmer (2015) computed via the self‐similar Rayleigh‐Gans approximation (Hogan & Westbrook, 2014; Hogan et al., 2017; Leinonen et al., 2017) are plotted in Figure A5. The range of variability found for a specific size illustrates the inherent uncertainty of scattering properties caused by the ice microphysics, with heavily rimed crystals backscattering up to 10–15 dB more than fluffy aggregates for the same mass content.

Reflectivities are significantly reduced when increasing frequencies, with Ka band reflectivities reaching a plateau with *D*_*m*_ exceeding 4 mm and W and G band reflectivity decreasing for *D*_*m*_ larger than approximatively 1.5 and 1 mm, respectively, for fluffy aggregates. The DFR to Rayleigh are plotted in the right panel of Figure A5 and clearly highlights the following:

1. The dependence of DFR on ice microphysics (compared fluffy aggregates and heavily rimed ice crystals by contrasting thick continuous and dashed lines);
2. The impact of the width of the PSD on DFR (envelope of shaded areas), see also Mason et al. (2019);
3. The enhanced sensitivity of DFR to crystal size when increasing frequency;
4. The shift of the onset of non‐Rayleigh effects, signified by the *D*_*m*_ at which *DFR* exceeds a given threshold (e.g., 1 or 3 dB in Table A1) toward smaller and smaller values of *D*_*m*_ when moving toward larger and larger frequencies and denser and denser particles (see detailed values in Table A1).

**Table A1:** Onset of Non‐Rayleigh Effects for Ice Crystals

	*D*_*m*_ (mm) for Aggregates		*D*_*m*_ (mm) for Rimed Crystals	
Frequency (GHz)	*DFR* > 1 dB	*DFR* > 3 dB	*DFR* > 1 dB	*DFR* > 3 dB
9.6	6.1	–	5.4	–
13.6	4.2	–	3.5	7.1
35.5	1.7	3.0	1.3	2.2
94.4	0.7	1.2	0.6	1.0
220	0.3	0.6	0.2	0.4

#### A.1.5 Attenuation Effects for Raindrops and Ice Crystals

The clear advantage of higher frequencies in terms of large dynamic range in non‐Rayleigh effects is strongly mitigated by the increase of attenuation that can cause reflectivities to drop below radar sensitivity thresholds, with frequencies above 200 GHz generally severely affected. Water/ice particle extinction coefficients per unit mass are shown in the left/right panel of Figure A6. For both ice and rain, extinction of the radar signal due to attenuation increases monotonically with frequency. For large raindrops (*D*_*m*_>2 mm), extinction saturates at W band as a consequence of scatterers reaching the optical region and the geometric optics limit (Bohren & Huffman, 1983). On the other hand, while ice extinction monotonically increases with size, raindrops with mean mass‐weighted equivolume diameters of 1 and 0.45 mm maximize the attenuation coefficients at 94 and 220 GHz, respectively. The ice density significantly affects the attenuation properties, with heavily rimed crystals already producing nonnegligible attenuation at Ka band and fluffy aggregate attenuation generally negligible up to W band.

#### A.1.6 Retrieval Approaches

Non‐Rayleigh and attenuation effects are entangled in the observed *DFR*s and there are no simple methods to separate the two components. A way to separate the two components is via Doppler spectra as proposed by Tridon et al. (2013) by following a technique analogous to the high spectral resolution lidar technique. The technique has been applied to different ground‐based observations (Li & Moisseev, 2019; Tridon & Battaglia, 2015; Tridon et al., 2017). Since in spaceborne observations from LEO Doppler spectra tend to be broadened by the satellite velocity, the technique is challenging with current spaceborne observation concepts (see discussion in section 4.6). Simple dual‐frequency retrieval algorithms have been developed for regimes where one effect is predominant. For instance, in conditions where attenuation can be neglected, sizing of ice and snow crystals can be achieved (Matrosov, 1998; Liao & Meneghini, 2011; Wang et al., 2005) whereas differential attenuation methods for the estimation of the cloud, drizzle, or light rain liquid water content have been proposed for scenarios containing Rayleigh targets only (Ellis & Vivekanandan, 2011; Hogan et al., 2005) or attenuation‐based methods have been used to retrieve precipitation (Matrosov, 2005; Matrosov et al., 2008). Radar observations at three (or more) frequencies have the potential to be effective in retrieving microphysical properties for a larger gamut of cloud and precipitation regimes where the simultaneous presence of both attenuation and non‐Rayleigh effects can substantially complicate the inversion process. As a result, inversion processes mainly based on optimal estimation algorithms have been proposed and applied to a variety of scenarios (Battaglia et al., 2016; Grecu et al., 2004; Grecu & Olson, 2008; Mason et al., 2017, 2018; Tridon et al., 2019). In such schemes, additional vertically integrated information about the hydrometeor structure is highly desirable to further constrain and stabilize the inversion problem (Haynes et al., 2009; L'Ecuyer & Stephens, 2002). Examples of these are provided by radar path‐integrated attenuations (*PIA*), typically derived by the surface reference technique, and by radiometer brightness temperatures (*T*_*B*_s).

In the presence of ice particles for which attenuation is mainly caused by scattering and when footprints become comparable to the mean scattering length, the single scattering approximation (Equation A2) is not valid any more and MS effects become important. This further complicates the inversion problem, and reconstructing the state of the atmosphere in the presence of non‐Rayleigh, attenuation, and MS effects becomes extremely challenging. New retrieval frameworks based on optimal estimation techniques and fast forward models capable of accounting for attenuation and MS are now available (e.g., Battaglia et al., 2015; Battaglia, Mroz, Lang, et al., 2016; Delanoë & Hogan, 2010; Hogan & Battaglia, 2008; Lebsock & L'Ecuyer, 2011; Mason et al., 2017).
